# Harmony COVID-19: A ready-to-use kit, low-cost detector, and smartphone app for point-of-care SARS-CoV-2 RNA detection

**DOI:** 10.1126/sciadv.abj1281

**Published:** 2021-12-15

**Authors:** Nuttada Panpradist, Enos C. Kline, Robert G. Atkinson, Michael Roller, Qin Wang, Ian T. Hull, Jack H. Kotnik, Amy K. Oreskovic, Crissa Bennett, Daniel Leon, Victoria Lyon, Shane D. Gilligan-Steinberg, Peter D. Han, Paul K. Drain, Lea M. Starita, Matthew J. Thompson, Barry R. Lutz

**Affiliations:** 1 Department of Bioengineering, University of Washington, Seattle, WA, USA.; 2 Global Health for Women, Adolescents, and Children, School of Public Health, University of Washington, Seattle, WA, USA.; 3 Department of Family Medicine, University of Washington, Seattle, WA, USA.; 4 Department of Genome Sciences, University of Washington, Seattle, WA, USA.; 5 Brotman Baty Institute for Precision Medicine, Seattle, WA, USA.; 6 Departments of Global Health, Medicine, and Epidemiology, University of Washington, Seattle, WA, USA.

## Abstract

RNA amplification tests sensitively detect severe acute respiratory syndrome coronavirus 2 (SARS-CoV-2) infection, but their complexity and cost are prohibitive for expanding coronavirus disease 2019 (COVID-19) testing. We developed “Harmony COVID-19,” a point-of-care test using inexpensive consumables, ready-to-use reagents, and a simple device. Our ready-to-use, multiplexed reverse transcription, loop-mediated isothermal amplification (RT-LAMP) can detect down to 0.38 SARS-CoV-2 RNA copies/μl and can report in 17 min for high–viral load samples (5000 copies/μl). Harmony detected 97 or 83% of contrived samples with ≥0.5 viral particles/μl in nasal matrix or saliva, respectively. Evaluation in clinical nasal specimens (*n* = 101) showed 100% detection of RNA extracted from specimens with ≥0.5 SARS-CoV-2 RNA copies/μl, with 100% specificity in specimens positive for other respiratory pathogens. Extraction-free analysis (*n* = 29) had 95% success in specimens with ≥1 RNA copies/μl. Usability testing performed first time by health care workers showed 95% accuracy.

## Teaser

A unique four-plexed RT-LAMP test kit operated by health workers can provide faster, more sensitive results than laboratory tests.

## INTRODUCTION

In 2019, an outbreak in China of severe acute respiratory syndrome coronavirus 2 (SARS-CoV-2), the causative pathogen of coronavirus disease 2019 (COVID-19), rapidly became a global pandemic (*1*), and after a year, it has infected 100 million people and killed 2 million people (*2*). Multiple measures have been used to contain the spread of COVID-19. Governments imposed universal “stay-home” orders (*3*) to minimize transmission, which, in turn, has harmed mental health, social life, and the economy (*4*). Available vaccines do not completely prevent SARS-CoV-2 infections (*5*). Vaccinated individuals with breakthrough infections can transmit SARS-CoV-2 as much as unvaccinated individuals (*6*). Widespread COVID-19 testing and contact tracing is still a solution for sustained reopenings, allowing subsets of a community to resume work and begin to restore the economy. Moreover, testing is essential for the reopening of international borders. Several countries have enforced a “fit-to-fly” policy; international travelers must test negative for COVID-19 within 72 hours before boarding international flights (*7*). Fast and sensitive point-of-care (POC) testing could facilitate the safe return to functioning domestic and international economies. POC testing could allow testing to expand to geographic regions that have limited access to centralized laboratory facilities, enable essential businesses to regularly test employees, and allow easy and efficient community testing by public health officials and clinics. POC testing is critical in settings that require rapid turnaround time such as emergency urgent care facilities and points of introduction such as airports.

Here, we report the development of Harmony COVID-19—a complete moderate-throughput sample-to-result system for sensitive POC detection of SARS-CoV-2 RNA. Harmony simplifies testing with ready-to-use assay reagents, an easy-to-use dedicated smartphone interface, and an inexpensive isothermal heater/detector device to enable POC testing. The assay includes three redundant SARS-CoV-2 targets to avoid false negatives due to viral mutation and an internal amplification control (IAC) in each test to avoid false negatives in case of assay failure. The heater/detector detects two-color real-time fluorescence for the SARS-CoV-2 redundant targets and IAC, and results are automatically reported on the smartphone. We evaluated the system using two panels of specimens—a panel of extracted nasal specimens from individuals with respiratory symptoms and a panel of contrived specimens from the XPRIZE Rapid COVID Testing competition—and we conducted usability testing of Harmony by health care workers (HCWs). These evaluations have demonstrated that Harmony is a complete system for sample-to-result testing that is highly accurate in moderate complexity laboratories and clinic-based patient care settings.

## RESULTS

### Workflow and operation of Harmony COVID-19

**Fig. 1.**
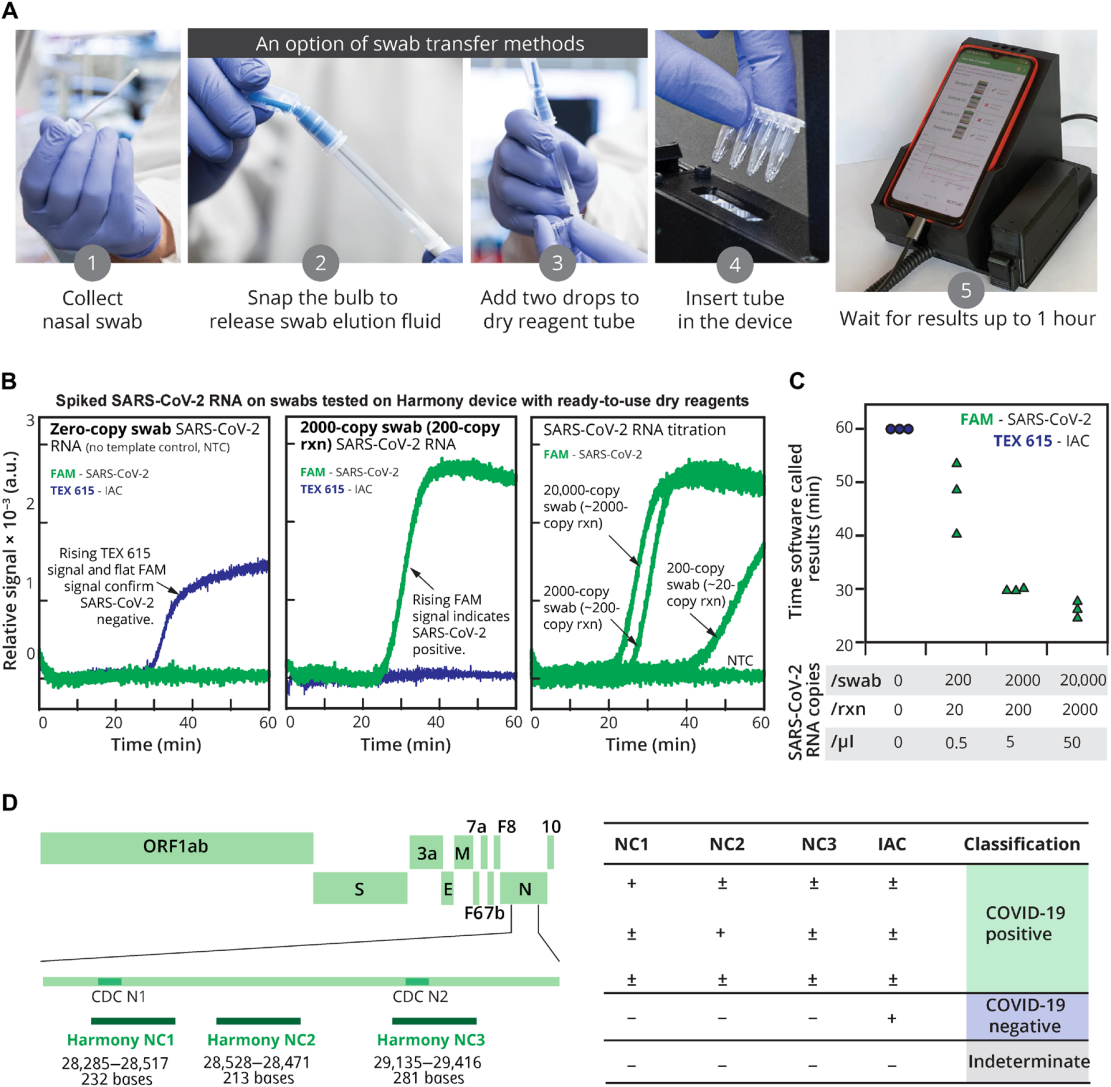
Harmony COVID-19 workflow, analytical performance, and interpretation. (**A**) User workflow. A nasal swab is collected and eluted in the rehydration buffer using either a unified sampler dispenser (shown) or a tube-and-bulb method (Fig. 8). The swab eluate is then transferred into the reaction tube to rehydrate lyophilized and preloaded RT-LAMP reagents. Users follow the instructions on the cell phone app to record the sample identity, insert the tube in the test slot, and close the device. Four samples can be analyzed in parallel. Photo credits: (1) to (4), Mark Stone at the University of Washington; and (5), Christopher Snyder at North Seattle College. (**B**) Examples of on-device analysis. Lyophilized reagents were rehydrated with the eluate from swabs spiked with 0, 200, 2000, or 20,000 copies of SARS-CoV-2 RNA, run on the device, and analyzed in real time. The samples with SARS-CoV-2 RNA at 2000, 200, and 20 copies per reaction (rxn) were reported as positive by software at 27, 30, and 41 min, respectively; after 60 min, the NTC reaction was classified as negative. a.u., arbitrary units. (**C**) Time to result. Individual detection time points (*n* = 3) are plotted. Variation in detection time increases as samples approach the detection limit. (**D**) Diagnostic algorithm. Harmony COVID-19 software calls samples as positive, negative, or indeterminate (IND) based on detection of the three regions in the nucleocapsid gene [Harmony NC1 overlapping with the CDC N1 region, Harmony NC2 (overlapping with CDC N3 region excluded from the current kit), and Harmony NC3 overlapping with the CDC N2 region] and the engineered IAC sequence.

The Harmony test kit and detector were engineered for low cost and simplicity of use to enable POC testing (Fig. 1A). The test involves taking a nasal swab, eluting the swab in a nontoxic buffer, and transferring the buffer to a reaction tube containing ready-to-use reagents. The reagent tube is inserted into the custom-designed heater/reader operated by a cell phone that provides instructions and displays the result. For samples with no target [Fig. 1B, left; no template control (NTC)], detection of the IAC confirms that the reaction was functional; the absence of both target and IAC amplification indicates a failed reaction. For samples with SARS-CoV-2 RNA (Fig. 1B, middle; 200 copies per reaction, corresponding to five copies/μl), the green signal indicates a positive test, regardless of whether IAC is detected. Harmony using dry reagents detects down to 200 copies of SARS-CoV-2 RNA on a swab (Fig. 1B, right; 20 copies per reaction, corresponding to 0.5 copies/μl). Real-time detection reports a positive sample as soon as the target signal appears, allowing detection in <30 min for samples ≥2000 copies per swab (Fig. 1C), with earlier results for higher viral load.

### Novel modifications of reverse transcription loop-mediated isothermal amplification core assay chemistry

The Harmony assay uses a unique variation of reverse transcription loop-mediated isothermal amplification (RT-LAMP) that allows fluorescence detection of multiple targets in the same reaction. The four-plexed assay amplifies three nonoverlapping genomic regions (Fig. 1D) of the SARS-CoV-2 nucleocapsid phosphoprotein (N gene) reported by a green fluorescence signal [6-carboxyfluorescein (FAM)] and an engineered IAC reported by a red fluorescence signal (TEX 615 or Texas Red—used interchangeably). A positive test result is reported when any of the three SARS-CoV-2 targets are present. Presence of IAC amplification is not necessary in a positive test. A negative test result is reported if SARS-CoV-2 RNA is undetected, and the IAC is detected. If neither target nor IAC is detected, then it indicates a test failure, and the result is reported as indeterminate (IND).

Harmony primers target the SARS-CoV-2 *N* gene. On the basis of the initial design process in early 2020, alignments of related coronaviruses [e.g., SARS-COV-1, Middle East respiratory syndrome (MERS), and bat coronaviruses] confirmed that the N gene was likely to be a well-conserved target (not the most conserved region) but was sufficiently divergent from related species to be organism specific. At the time the sequence diversity of the virus was very low; thus, further SARS-CoV-2–specific analysis of the N gene by sequence analysis was not possible. Therefore, primer design decisions were based on performance-driven parameters such as computational estimates of primer properties (e.g., homodimers, hairpin structures, Tm, and %GC). Our recent in silico analysis revealed that our primers still have high coverage across new variants (*8*). The specific overlap with the U.S. Centers for Disease Control and Prevention (CDC) primers (Fig. 1D) was semicoincidental and possibly a consequence of shared primer design requirements as suitable regions evaluated by analysis software, and the small size of the N gene [1275 base pairs (bp)] relative to the footprint of our LAMP assays (213 to 281 bp). The proximity of our LAMP amplification targets on the same contiguous DNA fragment may have an extra advantage to enable a synergistic initiation effect. Amplification events could occur closely enough such that they could bump each other. Other RT-LAMP technologies (table S1) that amplify different genes would not benefit from this effect. For the IAC amplification, we used a single LAMP loop primer to intentionally impair the IAC amplification (table S2) so that it would not outcompete SARS-CoV-2 amplification.

Harmony also uses novel universal probes containing entirely engineered sequences (*8*). The 5′ FAM or 5′ TEX 615 signal probes each have a 5′ sequence region hybridized to the complementary sequence region of its quencher probe that has 3′ Iowa Black FQ or 3′ BHQ-2, respectively. In the absence of target or IAC amplification, these quencher molecules remain proximal to their fluorophore pairs and absorb the fluorophore’s emission wavelengths. The signal probes each have a 3′ overhang with a sequence region identical to the 5′ overhang of the loop primer for target or IAC. In the presence of target or IAC amplification, quencher probes are displaced, enabling signal detection.

**Fig. 2.**
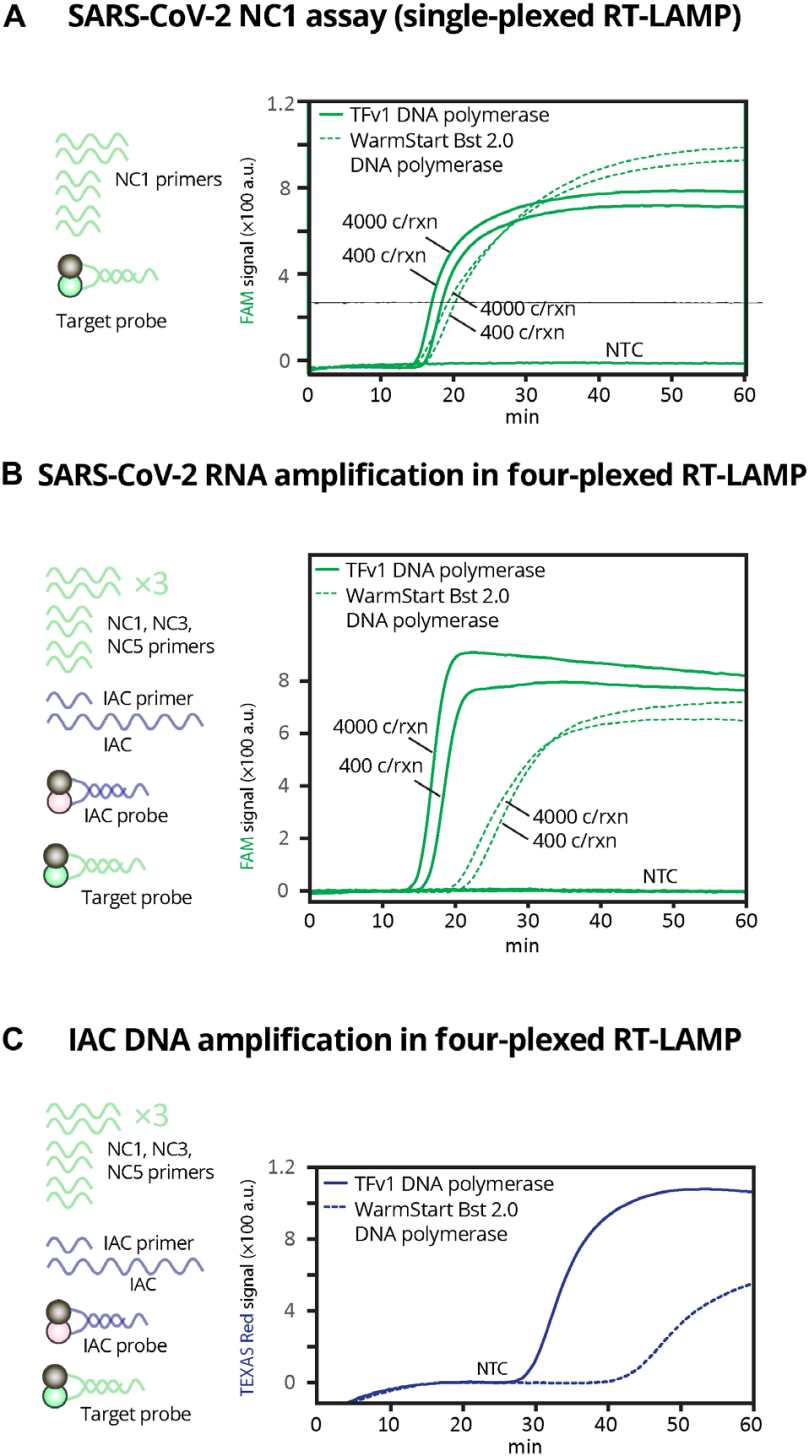
Comparison of RT-LAMP using TFv1 polymerase versus commercial Bst polymerase using conventional RT–quantitative polymerase chain reaction machine. (**A**) FAM signal of the RT-LAMP assay using fresh reagents (not lyophilized) containing only NC1 primer set (six primers total) and SARS-CoV-2 detection probe and quencher with 0, 400, or 4000 SARS-CoV-2 RNA copies per reaction (corresponding to 0, 100, or 1000 SARS-CoV-2 RNA copies/μl, respectively). (**B**) FAM signal and (**C**) IAC signal of the four-plexed RT-LAMP assay using fresh reagents containing all primers (18 SARS-CoV-2 primers and 1 IAC primer), SARS-CoV-2 detection probe and quencher, and IAC detection probe and quencher with 0, 400, or 4000 SARS-CoV-2 RNA copies per reaction (corresponding to 0, 100, or 1000 SARS-CoV-2 RNA copies/μl, respectively). For (C), only NTC is shown to indicate true negative. All reactions were incubated at 63.3°C for 1 hour and read every 13 s in a commercial thermal cycler.

To facilitate Harmony’s multiplexed RT-LAMP, we used newly developed, strand-displacing TFv1 chimeric DNA polymerase. Compared to the WarmStart Bst DNA polymerase 2.0 commonly used in RT-LAMP SARS-CoV-2 assays (table S1) (*9*, *10*), TFv1 chimeric DNA polymerase has a comparable amplification rate in a single-plex RT-LAMP (Fig. 2A) but a drastically faster amplification of SARS-CoV-2 (Fig. 2B) and IAC targets in our four-plexed RT-LAMP (Fig. 2C).

### Ready-to-use reagents

**Fig. 3.**
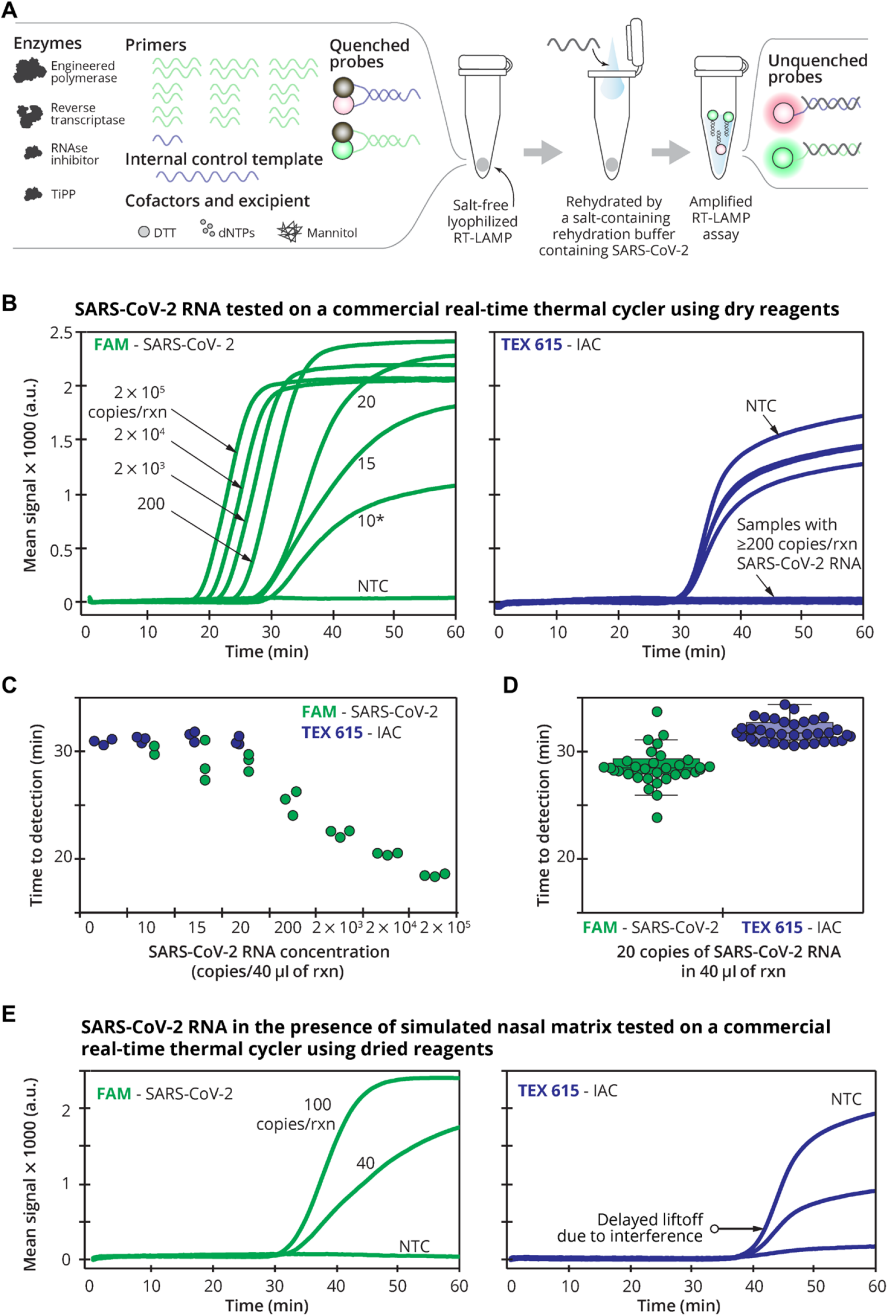
Lyophilized RT-LAMP and its analytical sensitivity. (**A**) To set up the assay, a sample in elution/rehydration buffer containing magnesium, ThermoPol buffer, and detergents is added to rehydrate the lyophilized RT-LAMP reagents in a single step. RNase, ribonuclease; TiPP, thermostable inorganic pyrophosphatase; DTT, dithiothreitol; dNTPs, deoxynucleotide triphosphates. (**B**) Real-time amplification signal of RT-LAMP reactions containing SARS-CoV-2 RNA (NTC), 10, 15, 20, 200, 2000, 2 × 10^4^, or 2 × 10^5^ copies/40 μl of reaction. Left: The average (*n* = 3) FAM signal detecting SARS-CoV-2. *Only two of the three replicates of 10 copies per reaction amplified so the average was from duplicate reactions. Right: TEX 615 signal detecting the IAC. The IAC was detected in the NTC (*n =* 3) and in the single 10 copies per reaction that did not detect SARS-CoV-2. (**C**) Time to detection of SARS-CoV-2 and IAC targets reported by the real-time thermal cycler. Individual data points are plotted. Note that only two of the three replicates of 10 copies per reaction amplified. IAC signals were properly detected in NTC (*n* = 3) and one of the three replicates of 10 copies per reaction undetected for SARS-CoV-2. (**D**) Evaluation of analytical sensitivity at 20 copies per reaction of SARS-CoV-2 RNA (*n* = 40), performed in two different runs (*n* = 20 each) with two different serially diluted RNA samples. Time to result of individual samples is plotted along with medians (middle lines of the boxes), interquartile ranges (edges of the boxes), ranges (the ends of whiskers), and outliers (data points outside the whiskers). (**E**) Impact of nasal matrix. SARS-CoV-2 RNA at 0, 40, or 100 copies per reaction (corresponding to 0, 1, or 2.5 copies/μl) was amplified in lyophilized RT-LAMP in the presence of simulated nasal matrix (*n* = 3). All data were measured every 13 s using FAM and TEX 615 channels by a real-time thermal cycler in 1-hour reactions at 63.3°C.

Our lyophilized RT-LAMP mixture contains primers, sequence-specific fluorescence probes, IAC DNA template, polymerases, supporting proteins, and cofactors (Fig. 3A) and is activated by adding the nasal swab eluate containing additional buffer, salts, and detergent. We initially screened excipient formulations that can preserve enzymatic activity and do not drastically interfere with the primer or probe behaviors. We selected trehalose and mannitol because they have been used as excipients for polymerase chain reaction (PCR) (*11*–*15*) and LAMP (*16*). We found that mannitol did not drastically affect the Harmony’s sensitivity compared to trehalose at the same concentration (fig. S1) and did not require adjusting the reaction temperature to maintain the assay speed. Possibly, this is due to the effect of trehalose on altering primer and probe melting temperatures (*17*).

Using a commercial real-time thermal cycler, lyophilized RT-LAMP detected three of three replicates of 15 RNA copies per reaction (0.38 copies/μl), and IAC was detected in all negative samples (Fig. 3B). RT-LAMP amplification of 2 × 10^5^ RNA copies per reaction using the dry reagents generated detectable signal in 17 min (Fig. 3C). We subsequently tested our in-house lyophilized reagents on a larger number of technical replicates of SARS-CoV-2 RNA at 20 copies per reaction (0.5 copies/μl) using multiple batches, and 90% [36 of 40, 95% confidence interval (CI): 76 to 97%] were amplified (Fig. 3D). Medians of time to detection for FAM and TEX 615 signals were 28.6 min (range, 28.0 to 29.4) and 31.7 min (range, 31.1 to 32.7), respectively. On the basis of these preliminary experiments, our limit of detection (LoD) is 15 to 20 copies per reaction (0.38 to 0.5 copies/μl) when analyzing purified, synthetic SARS-CoV-2 RNA. Next, we tested our lyophilized RT-LAMP reactions with simulated nasal matrix (salt, mucin, and human DNA) to mimic human nasal samples. We observed a 10-min delay in amplification of both SARS-CoV-2 RNA and IAC DNA (Fig. 3E), but all targets were detected correctly. Greater interference or other harm to the reaction will further delay IAC amplification; thus, the IAC serves as an indicator of the reaction’s function and can prevent reporting false negatives due to interference or damaged reagents. By setting a 60-min reaction time, the assay can suffer some delay while still correctly identifying positive samples. Specificity testing against closely related pathogens MERS and SARS-CoV-1 showed no cross-reactivity (fig. S2).

### Hardware: Housing and real-time fluorescence reader device

**Fig. 4.**
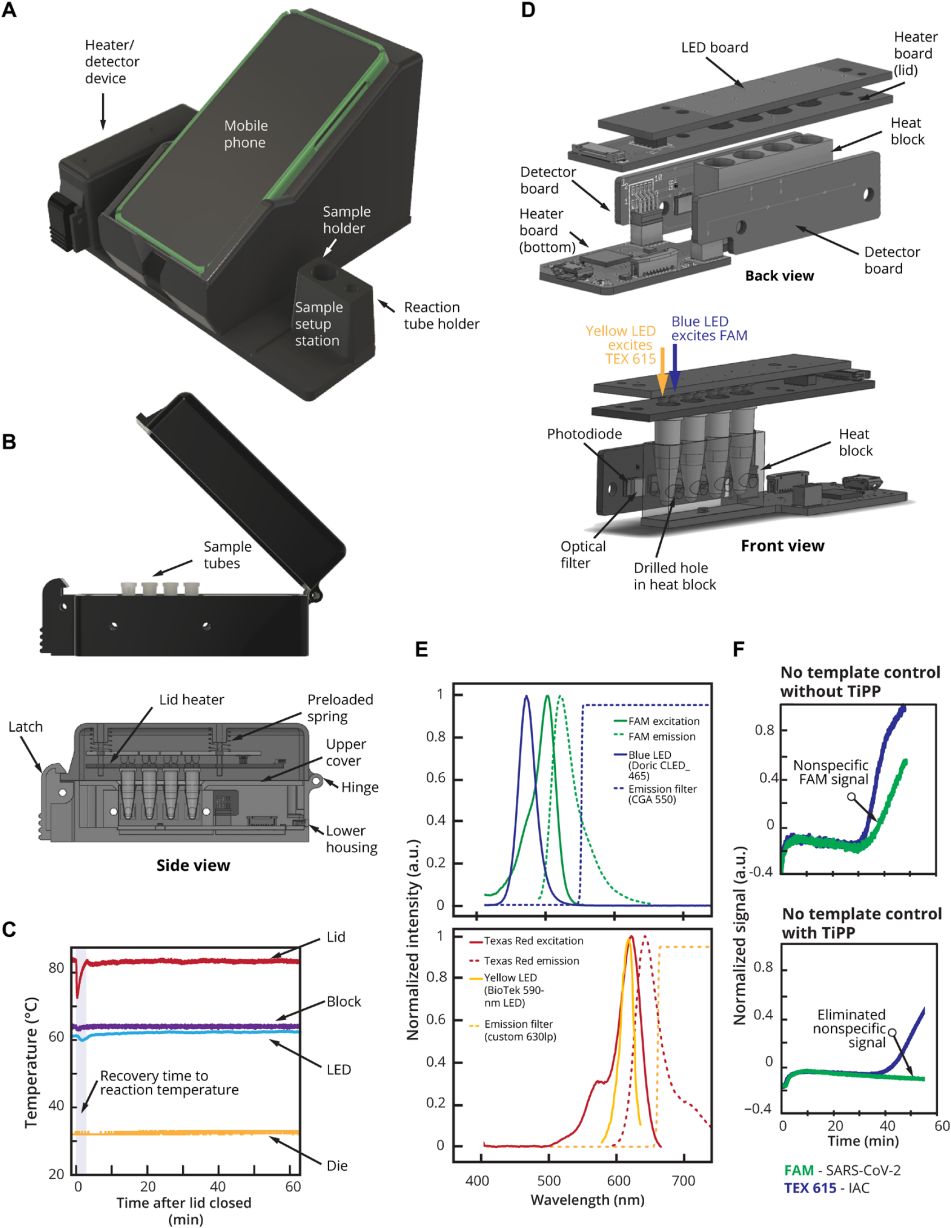
Overview of Harmony COVID-19 hardware. (**A**) Plastic case cradles the cell phone (removable and connected by a spring cable), mounts the heater/reader device, and houses a Charge-Plus USB-C connector hub (not shown). A sample station holds the sample and reaction tube during processing and is detachable to allow cleaning. Weight: housing, 266 g; heater/reader device, 104 g (total < 0.5 kg). Dimensions: housing, 205 mm by 167.5 mm by 125 mm; heater/reader device, 30 mm by 120 mm by 50 mm. (**B**) The heater/reader device contains an aluminum block with wells for four samples (0.2-ml reagent tubes) and is heated from the bottom by a resistive heater made from serpentine conductive traces on a printed circuit board. In the middle of the heater, a temperature sensor [Class B Platinum resistance temperature detector (RTD)] is used to measure the heat block temperature. Power to the heater is supplied by a metal-oxide-semiconductor field-effect transistor (MOSFET) and modulated by varying the pulse-width modulation (PWM) duty cycle of the MOSFET. (**C**) Temperature profile after the lid opening throughout a 60-min assay run. The microcontroller (die) is measured to ensure that the electronics are not overheated. (**D**) Lid heater, LED, and detector. A heater (85°C) on top of the tube prevents condensation on the tube lid. The lid heater is spring loaded to apply a preload force to the tubes to ensure good thermal contact and contains pass-through holes for excitation LEDs. (**E**) Spectral characteristics of the detection system. Blue and yellow LEDs provide excitation for FAM and TEX 615, respectively, and photodetectors on each side detect light passing through emission filters [spectral information from manufacturers (*41*–*43*)]. The LEDs are operated independently (only one LED is illuminated at a given time). (**F**) Effect of TiPP on RT-LAMP signal in the Harmony device. Normalized signal of negative control samples using the RT-LAMP formulation with and without TiPP (*n* = 1 each) at 64°C.

The Harmony system includes a heater/detector device controlled by a dedicated cell phone and plastic housing with a sample setup station (Fig. 4A). The heater/detector device (Fig. 4B) includes an aluminum heater block with four wells and a spring-loaded heated lid to prevent condensation. A proportional integral derivative feedback loop running on a microcontroller allows recovery of the temperature setpoint within 3 to 4 min after disturbance from opening the lid (Fig. 4C). Two light-emitting diodes (LEDs) (Fig. 4D) provide the excitation light by shining down through the top of each sample tube. Fluorescence emission passes through holes drilled on the side of the heater block. Each sample well has an emission filter/photodiode set on each side to detect two emission wavelengths (Fig. 4E). A blue LED (dominant wavelength of 470 nm) is used to excite FAM, and the emission photodiode sits behind a dielectric 550-nm-long pass filter. A yellow LED (dominant wavelength of 587 nm) is used to excite TEX 615, and a second emission photodiode sits behind a 630-nm-long pass dielectric filter.

### Thermostable inorganic pyrophosphatase enables real-time LAMP detection in the Harmony COVID-19 device

RT-LAMP generates inorganic pyrophosphates (PPi) that precipitate from the solution. The cloudiness created by precipitates has been used as a visual readout signal for LAMP (*18*), but here, the cloudiness scattered light and caused nonspecific signals in NTC samples (Fig. 4F, top) when tested on device. Thermostable inorganic pyrophosphatase (TiPP) can be added to the LAMP reaction to hydrolyze built-up pyrophosphate, and this combination has been used in other contexts to improve differentiation of LAMP products in melt analysis (*19*) and indirectly detect LAMP via phosphate ions hydrolyzed by the TiPP (*20*). TiPP was advertised for its use for the enhancement of DNA replication (*21*), mitigating pyrophosphorolysis during DNA synthesis for sequencing (*22*). In our context, we added TiPP in the RT-LAMP reaction to eliminate scattered signal from pyrophosphate to prevent nonspecific signal in negative reactions (Fig. 4F, bottom). This effect was not observed when the reaction was tested in the commercial thermal cycler (Bio-Rad, CFX96). This is potentially because the commercial thermal cycler uses band-pass filters for both excitation and emission wavelengths, while our device does not have excitation filters.

In addition to the physical effects, we have postulated that PPi accumulation may inhibit LAMP; as PPi forms, magnesium phosphate precipitate, Mg^2+^, will be sequestered from the reaction, potentially impairing polymerase activity and primer hybridization. TiPP addition reduces the turbidity that is indicative of magnesium phosphate precipitate, so it may limit the depletion of reaction components. This has implications for multiplexed LAMP reactions, where concurrent amplification of multiple targets is a desired outcome. This possibility or the specific capability of TiPP to “enhance” LAMP has not yet been evaluated.

### Multipurpose Android mobile app: Instruction, temperature control, and real-time analysis of results

**Fig. 5.**
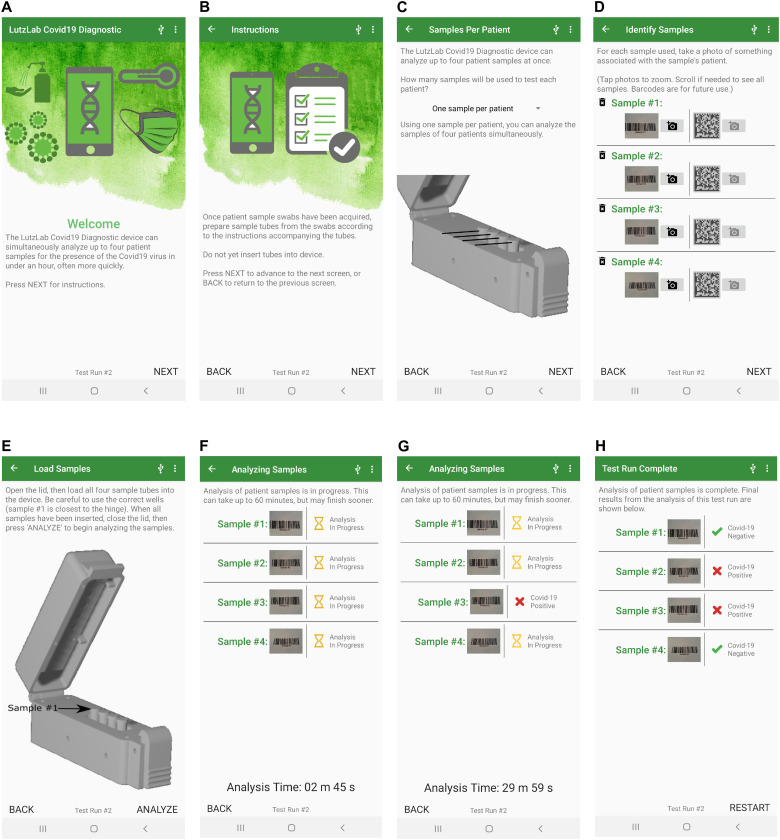
Cell phone user interface and result reporting. The Android Harmony application guides the user through steps to set up and run the test. (**A**) Intended use of the device. (**B**) Instructions for the user to prepare samples. (**C**) Selection of the number of samples. (**D**) Collection of sample identifiers by barcode, photo capture, or manual typing. (**E**) Instruction to insert the tube when the temperature of the device reaches the reaction temperature. (**F**) Real-time analysis during the test run. (**G**) Positive results are reported as soon as they are detected. (**H**) Negative and IND results are reported at the end of the run time.

The phone software provides interactive guidance for setting up the test. It prompts the user to input sample identification (ID) by scanning a barcode, capturing a photo, or typing a sample ID (Fig. 5, A to D). The software also carries out the closed-loop temperature control for the heater block and lid heater, and it displays the status of the heater block to prompt the user to insert the reaction tubes only after the heater reaches the reaction temperature (Fig. 5E). During the test run, the screen displays the test status (“analysis in progress”) and elapsed time, or it reports any detected errors (“failed run”) (Fig. 5F). The software performs real-time data analysis that detects fluorescence intensity rise above a dynamically and automatically computed background level. Signals from SARS-CoV-2 can appear early in the test, especially for high viral loads, and the real-time analysis allows reporting of positive tests immediately after they are detected (Fig. 5G). Negative results and IND results are reported at the end of the test run time (Fig. 5H). Software analysis is described in Materials and Methods.

### Evaluation Harmony COVID-19 system on contrived SARS-CoV-2 samples (XPRIZE panel) without extraction

**Fig. 6.**
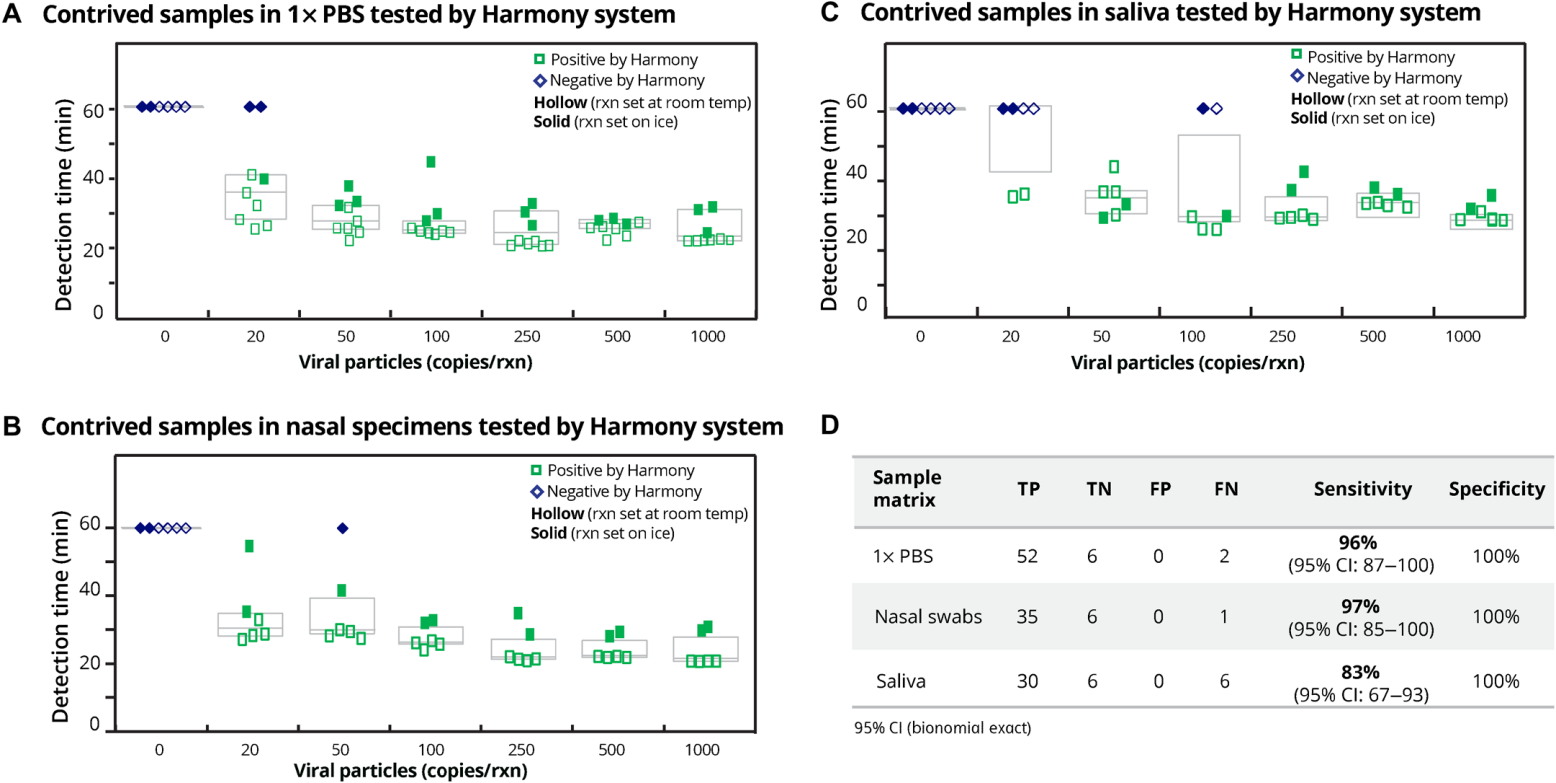
Analysis of XPRIZE contrived sample panel without RNA extraction. (**A**) Viral particles spiked in 1× PBS were tested in triplicate for each sample (*n* = 9 total for positive sample concentrations and *n* = 6 total for negative samples). (**B**) Viral particles spiked in human nasal matrix were tested in triplicate (*n* = 6 total for each concentration). (**C**) Viral particles spiked in saliva (*n* = 6 total for each concentration). (**D**) Summary table for the sensitivity and specificity of the Harmony COVID-19 system on the XPRIZE contrived samples. Concentrations of viral particles are the final concentrations in each 40-μl reaction. Threshold concentration for positive is at 20 viral particle copies per reaction (0.5 copies/μl). Contrived samples below the positive threshold were also tested (fig. S7). Real-time amplification curves of these data are in fig. S8. Reactions were set up either at room temperature (hollow) or on ice (solid).

A panel of contrived samples from the COVID-19 XPRIZE competition were tested on the Harmony system. For SARS-CoV-2 virus in 1× phosphate-buffered saline (PBS) (Fig. 6A), Harmony detected all positive samples containing ≥50 SARS-CoV-2 viral particles per reaction, and 96% of samples containing ≥20 viral particles per reaction. In human nasal matrix (Fig. 6B) and human saliva (Fig. 6C), Harmony detected 97 and 83%, respectively, of samples containing ≥20 viral particles per reaction. Harmony had zero false positives across all sample types. Unlike the case of simulated nasal matrix (Fig. 3E), we did not observe delayed detection time of IAC in human nasal or saliva samples. Detection times (means ± SD) for IAC amplification in negative samples were 32.9 ± 3.8 min for PBS, 35.8 ± 4.8 min for human nasal matrix, and 34.3 ± 11 min for human saliva, respectively (*P* > 0.05; *t* test, two-tailed). It is worth noting that in this experiment, RT-LAMP reactions were either set up at room temperature (intended protocol for our assay, hollow symbols) or on ice [protocol used by most emergency use authorization (EUA) RT-LAMP assays as summarized in table S1, solid symbols]. Even with a small sample size, we observed relatively delayed reaction and more false-negative results when the reaction was set up on ice, especially those near the LoD (i.e., 20 copies per reaction).

### Evaluation of clinical specimens using the Harmony COVID-19 system

We first tested the assay for reactivity with SARS-CoV-2 and cross-reactivity with other respiratory pathogens using RNA extracted from infected patient specimens. The retrospective clinical samples had been collected in viral transport medium (VTM), which is not the intended sample type for Harmony, because a POC test uses fresh samples and does not require sample transport. Subsequently, we tested a smaller panel enriched with positive specimens to assess the feasibility of detecting SARS-CoV-2 RNA in VTM specimens without extraction.

**Fig. 7.**
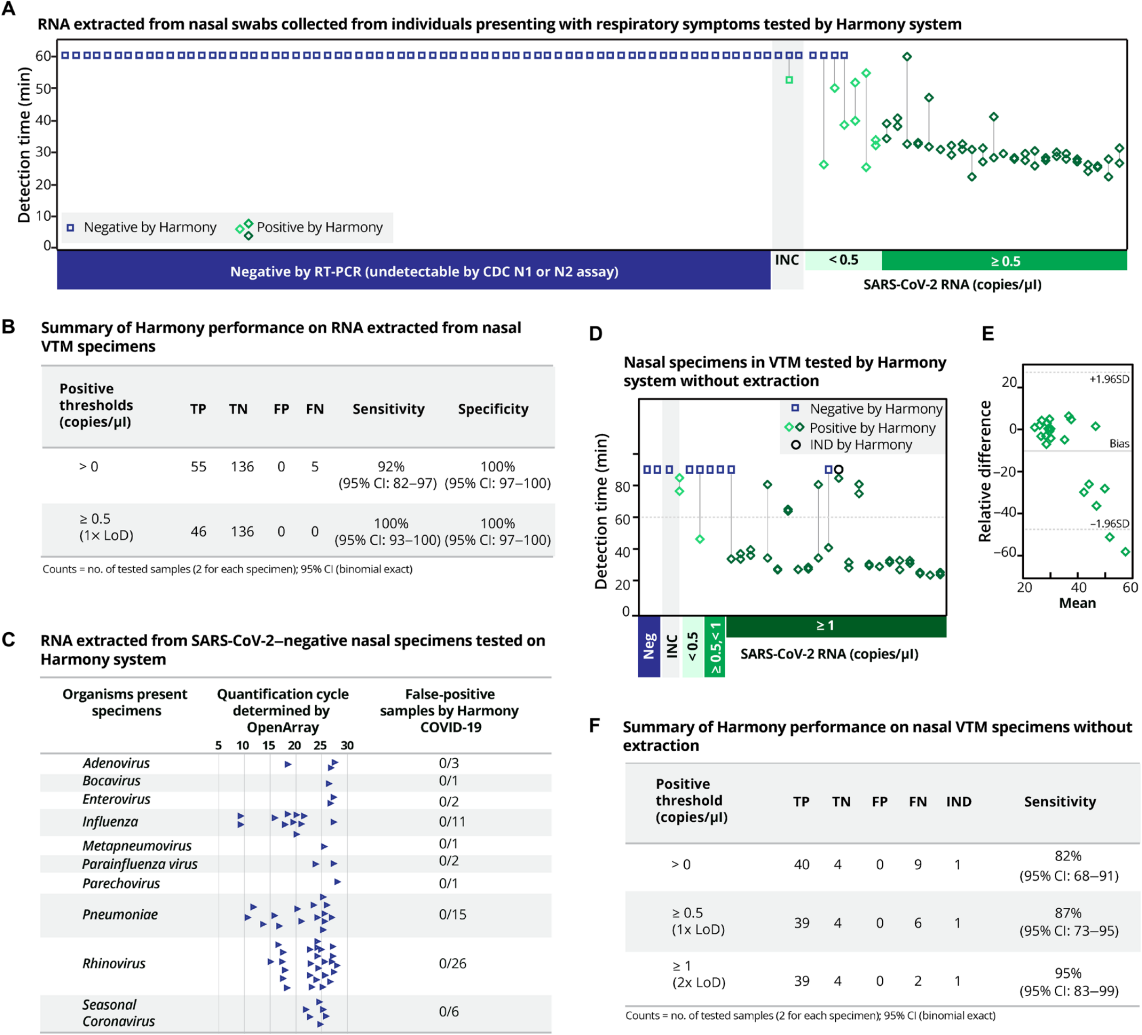
Harmony COVID-19 performance on clinical specimens. (**A**) Detection time by Harmony software for the analysis of RNA extracted from human nasal specimens from 101 patients presenting respiratory symptoms. Stored specimens were retested by RT-qPCR using N1 and N2 CDC primers; results matched previous results in 30 previous positive specimens (positive for N1 and N2) and 68 previous negative specimens, with three previous positive specimens classified as inconclusive (INC) because they were negative for either CDC N1 or N2 by RT-qPCR. Samples are ranked from left to right by increasing SARS-CoV-2 concentrations quantified by RT-qPCR. Extracted RNA from specimens was analyzed by Harmony (10-μl RNA in 40-μl dry RT-LAMP, *n* = 2). The classification results by Harmony were compared to RT-qPCR using CDC primers and probes (5-μl RNA in 20-μl RT-qPCR). (**B**) Organisms in clinical nasal specimens were quantified using OpenArray, as described (*35*). High-quantification cycle values indicate low concentrations. All these specimens were correctly identified as SARS-CoV-2 negative by Harmony. TP, true positive; TN, true negative; FP, false positive; FN, false negative. (**C**) Summary table for the sensitivity and specificity of the Harmony using extracted RNA from clinical specimens (RT-qPCR INC samples excluded). (**D**) Detection time by Harmony software for the analysis on 27 VTM specimens previously tested in (A). Without the RNA extraction step, 10 μl of VTM was added in the final 40-μl RT-LAMP reaction (*n =* 2). Dotted line indicates the detection time at 60 min. (**E**) Bland-Altman plot of the detection time of SARS-CoV-2–positive specimens of the direct VTM group compared to the extracted RNA group. Dotted lines indicate the acceptable bound of the difference of the detection time (means ± 1.96SD). (**F**) Summary table for the sensitivity of the Harmony using VTM specimens without extraction.

Clinical VTM specimens were collected from individuals positive for SARS-CoV-2 (*n* = 33) or for other pathogenic respiratory infections (*n* = 68) and were retested by RT–quantitative PCR (qPCR) using CDC primer and probe sequences (*23*) to measure the SARS-CoV-2 viral load and provide the reference test result. Using RT-qPCR with CDC primers/probes, 68 specimens were confirmed to be SARS-CoV-2 negative (Fig. 7A, left), 30 specimens were confirmed to be SARS-CoV-2 positive (Fig. 7A, right), and 3 previously positive specimens were only detected by one RT-qPCR assay (either N1 or N2) and classified as inconclusive (INC; Fig. 7A, middle).

Specimens were tested in duplicate on Harmony using the ready-to-use reagents (Fig. 7A), amplification curves are arranged from low to high virus concentration as quantified by RT-qPCR assay (figs. S9 and S10). Of the 30 RT-qPCR–positive specimens, Harmony detected 26 specimens in both technical replicates and 3 specimens in one of the two replicates. In the three RT-qPCR INC results, one sample was detected by Harmony in one of the two replicates. Excluding the RT-qPCR INC specimens and applying a stringent requirement for both Harmony replicates to be detected to report a positive test, Harmony achieved 96% accuracy (94 of 98, 95% CI: 88 to 98%), 87% sensitivity (26 of 30, 95% CI: 69 to 96%), and 100% specificity (68 of 68, 95% CI: 94 to 100%). If replicates are treated independently (Fig. 7B), then Harmony detected 55 of the 60 positive specimens (92% sensitivity, 95% CI: 82 to 97%). Harmony detected all positive specimens with ≥20 copies per reaction (0.5 copies/μl). The clinical panel (Fig. 7A) included 68 specimens that were SARS-CoV-2 negative but contained other respiratory pathogens including influenza, rhinovirus, and seasonal coronavirus (Fig. 7C), as determined using OpenArray (a laboratory PCR test with microarray detection) (*24*). Harmony correctly identified all SARS-CoV-2–negative specimens (Fig. 7A, left). Figure 7B summarizes the performance of Harmony on detecting extracted RNA from clinical VTM specimens.

Next, we used Harmony to detect SARS-CoV-2 in VTM specimens without RNA extraction. We expected the presence of microbial agents in the VTM to delay amplification, and thus, we ran the reaction for additional 30 min. Compared to RT-qPCR, Harmony with 90-min run time detected 18 of 24 positive specimens in both replicates and 3 of 24 in one of the replicates. Considering each replicate independently, Harmony detected 78% (32 of 41) and 95% (39 of 41) SARS-CoV-2 RNA–positive specimens within 60- and 90-min detection time, respectively. Detection time (means ± SD) of IAC in the negative samples with direct VTM amplification (43.9 ± 6.4 min) was significantly slower than the detection time with extracted RNA (38.5 ± 1.6 min; *P* = 0.03, *t* test, two-tailed). Pair-wise comparison among positive SARS-CoV-2 specimens (Fig. 7E) showed two specimens with significantly different detection time. Although VTM is not the intended sample type, Harmony detected 95% of SARS-CoV-2 VTM specimens that contained at least one copy per microliter (Fig. 7F).

### Feasibility testing: Sample-to-result system tested by HCWs and laboratory personnel

To complete the system, we developed a test kit that included all components needed for running a test, including the swab, elution buffer, a fluid transfer device, and a ready-to-use reagent tube. Swabs were preloaded with SARS-CoV-2 DNA to serve as a control sample to evaluate performance by HCWs and laboratory personnel (LPs).

**Fig. 8.**
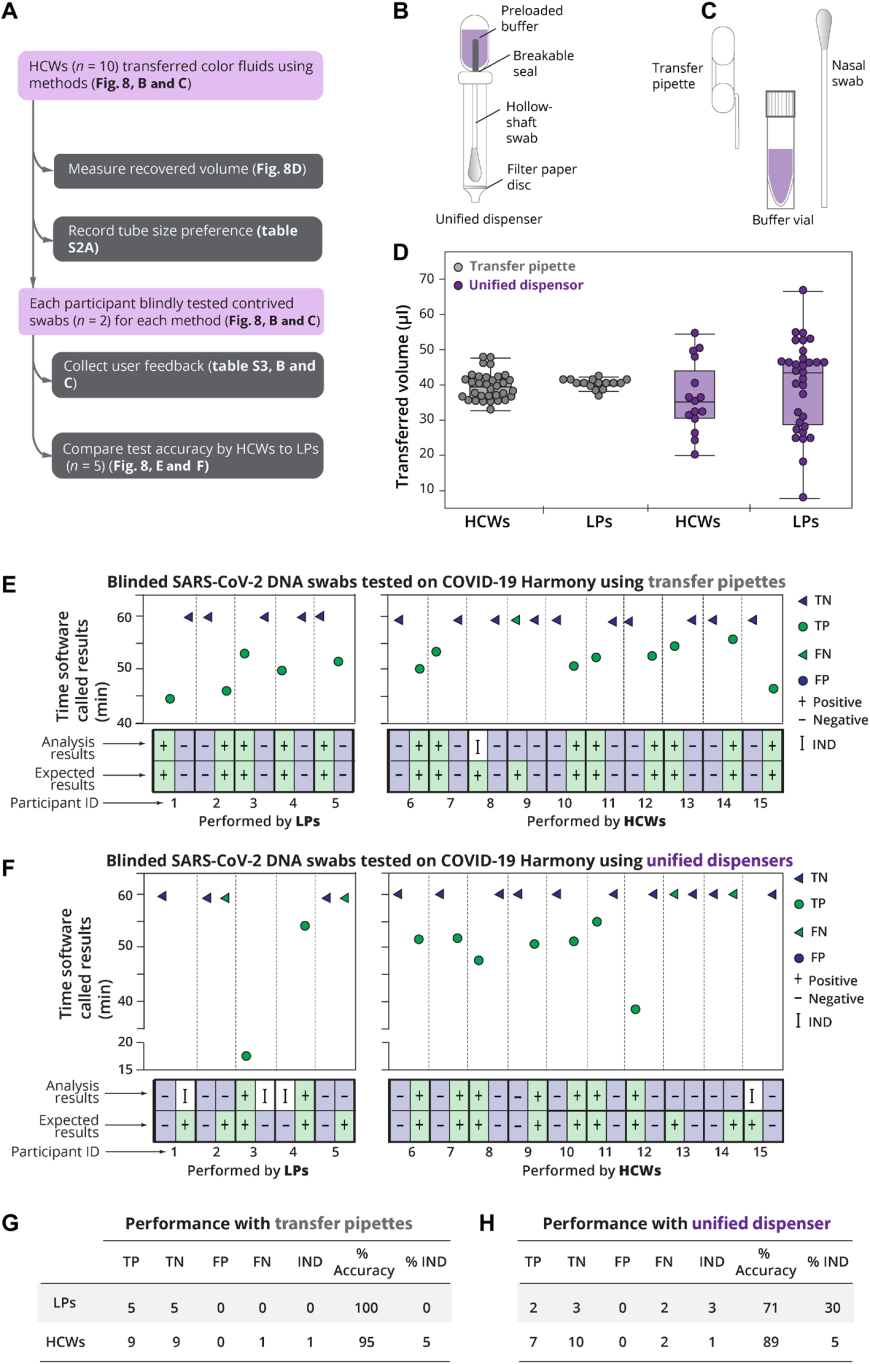
Usability study of Harmony COVID-19 workflow including two sample transfer methods at the POC. (**A**) Study design. (**B**) Illustration of the first sample transfer method option using a unified dispenser, which integrates a swab, buffer container, and a dropper in a single device. The contained buffer is released to elute the swab sample by cracking a conduit, the user agitates the fluid by shaking the tube, and a dropper tip allows the use to transfer two drops of fluid to the reaction tube by squeezing the body of the device. (**C**) Illustration of the second sample transfer method including a volumetric transfer pipette (40 μl), a nasal swab, and a preloaded buffer vial. Movie S1 demonstrates this workflow. Briefly, swabs are manually rubbed on the side of the tube for 30 s, soaked for 1 min, and removed. The pipette is then used to transfer the swab eluate to rehydrate the lyophilized RT-LAMP reaction tube. Movie S2 demonstrates this workflow. (**D**) Volume transferred by each method performed by HCWs or LPs. Individual volume measurements are plotted along with medians (middle lines of the boxes), interquartile ranges (edges of the boxes), and ranges (the ends of whiskers). (**E** and **F**) Assay performance on blinded contrived swabs (one negative swab and one positive swab with SARS-CoV-2 DNA at 1000 copies per swab, corresponding to 100 copies per reaction) by LPs (*n =* 5) and untrained HCWs (*n* = 10) using transfer pipettes (E) or unified dispensers (F). The usability experiment written protocol is available on protocol.io (https://dx.doi.org/10.17504/protocols.io.bkvskw6e) and as a visual demonstration in movies S1 and S2. (**G**) Performance summary for the transfer pipette method. (**H**) Performance summary for the unified dispenser method.

In the first phase of user testing (Fig. 8A), we compared the accuracy and reproducibility of two methods for transferring the eluate to the reaction tube when operated by LPs (*n =* 5) and HCWs (such as nurses, medical students, and dental students; *n =* 10). The first method (Fig. 8B and shown previously in Fig. 1) used a unified sampler dispenser. The second method (Fig. 8C) used a transfer pipette to transfer a fixed volume of fluid to the reaction tube. Regardless of the baseline skill of operators, transfer pipettes yielded more reproducible recovered volumes than the unified dispenser (*F* test, *P* < 0.001). Recovered volumes of the two methods were not significantly different between HCWs and LPs (Student’s *t* test, two-sided, *P* = 0.90; Fig. 8D). User feedback on the transfer methods and preferences on tube sizes are summarized in table S3. Next, HCWs and LPs executed the full Harmony workflow on contrived SARS-CoV-2 DNA swab samples (Fig. 8). Using the transfer pipette workflow (Fig. 8E), the test accuracy was 100% (10 of 10) for LPs and 95% (19 of 20) for HCWs. Unexpectedly, we found that the HCWs performed better than the LPs when using the unified dispenser system (Fig. 8F) when considering both accuracy and IND rates. HCWs had significantly lower (*Z* test, two-tailed, *P* < 0.05) IND results (1 of 20) than LPs (3 of 10) when using the unified dispenser system. Excluding IND results, the HCWs had 89% accuracy, higher than LPs with 71% accuracy (*Z* test, two-tailed, *P* < 0.05), when using the unified dispenser system.

## DISCUSSION

This work presents a combination of multidisciplinary engineering (molecular, mechanical, electrical, software, and human interface design) to develop a complete system for SARS-CoV-2 detection at the POC. The system is inexpensive and simple yet has features comparable to laboratory-based tests (Table 1A). The assay has high sensitivity and specificity in clinical and contrived specimens, and HCWs successfully performed the workflow with high accuracy.

**Table 1.** Feature comparison between the Harmony COVID-19 system and other assays.

**(A) Comparison to the U.S. CDC assay (*23*). Full cost calculations for reagents and devices are enclosed in tables S4 and S5.**					
**Item**	**Standard RT-PCR assay (based on the U.S. CDC** **protocol) (*23*)**		**Harmony COVID-19**		
SARS-CoV-2 primer detection	Separate reactions for N1 and N2		Multitarget redundancy amplifying three regions of the N gene in a single reaction tube		
Process control	Human RP gene		Engineered internal control		
LoD	1 to 3.2 copies/μl from extracted RNA		0.5 copies/μl (20 copies/40 μl of reaction) from synthetic RNA; 1 copy/μl (40 copies/40 μl of reaction) from extracted RNA from VTM; 2.5 copies/μl (100 copies/40 μl of reaction) from contrived nasal samples		
Consumable cost	$12.94 per sample ($6 extraction kit and		$8.00 per sample		
	$6.94 for three RT-PCR Taq Path/IDT probes)				
Easy-to-use reagents	No		Yes		
Batch size	24 specimens		Not required, run up to four samples		
Equipment cost	> $5000 qPCR machine		$247 customized heater/reader		
Time for sample to result	260 min		21 to 91 min		
	Hands-on: 120 min		Hands-on: 1 min for direct amplification		
	Wait time: 120 min		Wait time: 20 to 45 min for positive samples, but 90 min is required to confirm true negative		
	Interpretation: 20 min				
(**B**) Comparison of Harmony COVID-19 to other isothermal assays for SARS-CoV-2.					
Published assays	Amplification chemistry	Multitarget redundancy in a single tube	Reported LoD	User-friendly device with ready-to-use reagents and a closed system	Demonstration of use by HCWs
Harmony COVID-19 (this study)	RT-LAMP	Yes	0.5 to 2.5 copies/μl	Yes	Yes
Alekseenko *et al.* (*44*)	RT-LAMP	No	10 copies/μl	No	No
Rabe and Cepko (*45*)	RT-LAMP	No	50 copies/μl	No	No
Wei *et al.* (*46*)	RT-LAMP	No	2.5 copies/μl	No	No
Dao Thi *et al.* (*9*)	RT-LAMP	No	5 copies/μl	No	No
Bokelmann *et al.* (*47*)	RT-LAMP	No	5–20 copies/μl	No	No
Yamazaki *et al.* (*48*)	RT-LAMP	Yes	25 copies/μl	No	No
Qian *et al.* (*49*)	RPA	Yes	0.5 copies/μl	No	No
Patchsung *et al.* (*50*)	SHERLOCK	No	2.1 copies/μl	No	No
Joung *et al.* (*51*)	SHERLOCK	No	2 copies/μl	No	No
Broughton *et al.* (*52*)	CRISPR cas12	No	10 copies/μl	No	No

Harmony uses ready-to-use reagents that enables fast and accurate assay setup compared to laboratory PCR while maintaining high assay sensitivity and specificity. The lyophilized Harmony assay detected as low as 15 copies per reaction of synthetic SARS-CoV-2 RNA and detected all extracted RNA from clinical specimens at ≥20 copies per reaction with no false positives across all sample types. Harmony chemistry is also robust, enabling detection of contrived samples in human nasal matrix (intended sample type) and saliva without RNA extraction. We found excellent sensitivity when testing samples with ≥20 viral particles in nasal matrix (estimated 500 copies per swab for 1 ml of elution buffer and 40 μl of reaction). In saliva samples at the same concentrations (not the intended sample type), Harmony showed moderate sensitivity. Heat treatment of saliva shown to enable detection by RT-PCR (*25*) would likely improve the sensitivity of Harmony with saliva samples. Regardless, our current level of assay sensitivity would be sufficient to detect SARS-CoV-2 in most nasal specimens (~10^3^ to ~10^9^ copies per swab) (*26*) or saliva (~10^4^ to ~10^8^ copies/ml) (*27*) from infected individuals during the first week after the onset.

Harmony’s high assay sensitivity and specificity despite its high multiplexity was made possible by multiple modifications to traditional RT-LAMP. We used novel polymerase and probe design that can specifically discriminate between the SARS-CoV-2 from IAC amplification, while most RT-LAMP analysis in tube relies on detection of total amplification and splits each RT-LAMP reaction to detect only one target per reaction (table S1). Multitarget amplification is essential to reduce false negatives in SARS-CoV-2 nucleic acid tests as the new variants continue to emerge.

In addition to the core assay, our lyophilization process also involves nontraditional modifications. Unlike other lyophilized RT-LAMP (*28*), our lyophilized reagents are magnesium-free, which prohibits any enzymatic activity that may occur before assay initiation. Our lyophilized RT-LAMP is activated upon the addition of sample that contains magnesium and detergent. Thus, it should be less prone to moisture degradation than magnesium-containing lyophilized RT-LAMP. Second, RT-WarmStart enzyme in Harmony assay is inactive at room temperature and thus prevented activities during the reaction setup and eliminated the need for setting up the reaction on ice as performed in other SARS-CoV-2 LAMP assays (table S1). Cold temperature can promote dimer and/or secondary structures of LAMP primers, which we observed as a negative impact on the assay sensitivity and speed. While setting up Harmony assay for lyophilization, RT-LAMP primers and probes were heated and cooled down passively at room temperature to minimize secondary structures before mixing with the other components. Last, we removed water to reduce the final volume of lyophilized reaction to 20 μl. This enabled doubling the excipient concentrations in the lyophilized pellet and shortens the lyophilization drying time. When testing samples, the excipients are then diluted by half to 40 μl of reaction, thus reducing any impact of excipients on the core assay function. We anticipate that these components will be stable up to 30°C, but future experiments will be conducted in the final form of the product for future commercialization efforts.

Isothermal amplification, like RT-LAMP in Harmony, is often promoted as ideal for POC testing because it can be carried out using a constant temperature heat source, such as a water bath, heat block, or even more novel sources (chemical heat source, sunlight, and body temperature) (*29*). However, reported tests often have other limitations that may prevent their use in POC testing (Table 1B). Common limitations include use of frozen reagent stocks that must be thawed and then formulated using laboratory pipettes, manual steps for sample processing that require laboratory skill, or detection steps that rely heavily on user interpretation or analysis by a user’s cell phone. Harmony is a complete sample-to-result system that uses ready-to-use reagents for simple 1-min setup and a dedicated device that reports results without extra postamplification steps or reliance on user interpretation.

Some RT-LAMP tests use end-point lateral flow strips for detection (table S1), which adds a user step but, more importantly, should not be done at the POC because it exposes the testing site to amplicons that will give false-positive results in subsequent tests. Others use in-tube detection by eye (*30*) or a cell phone (*31*), which increases the user burden and could introduce additional sources of error. Real-time detection used in Harmony COVID-19 removes the need for extra detection steps or user interpretation. Real-time detection also allows Harmony to report positive results as soon as the SARS-CoV-2 signal appears rather than waiting for an end-point detection method. High–viral load samples could be detected in as little as 20 min, which could enable more timely infection control measures to limit viral spread. Harmony real-time detection allows testing without opening tubes after amplification, requires no extra steps or interpretation by the user, and allows reporting a positive sample earlier than end-point detection methods.

The Harmony device automates heating, multiplexed detection, and data interpretation. We integrated a simple heater with low-cost LEDs and sensors to enable real-time two-color detection of RT-LAMP fluorescence in four independent sample wells. The device costs <US$300 in parts for prototypes, while the lowest cost of a commercial RT-PCR machine on the market is >US$5000 (*32*). Moreover, the Harmony device is portable and energy efficient, requiring <15 W and operable by USB power sources. These features are attractive for use at the POC in the United States and resource-limited settings.

Last, the simple workflow and analysis of Harmony is appropriate for POC testing. Test operation involves a few simple steps that take ~1 min, and there are no further user steps after starting the test. Here, HCWs were able to operate the tests with simple written instructions and a video showing the procedures. The simplicity of the Harmony procedure and completeness of the stand-alone system can be a significant advantage for onboarding to ambulatory care settings and potentially community testing locations. Compared to existing U.S. Food and Drug Administration EUA RT-LAMP tests (table S1), Harmony is very competitive. Most RT-LAMP technologies are not designed for use in CLIA (Clinical Laboratory Improvement Amendments of 1988)-waived settings. Lucira is the only product approved for use at the POC, but it is custom-designed to be battery-powered and completely disposable. This strategy, while is convenient for users, can contribute to electronic waste and relatively high cost for producing a unit. In contrast, Harmony relies on low-cost consumables and generates no electronic waste after each run.

In this study, we evaluated the system using contrived specimens in nasal and saliva matrix (XPRIZE) and clinical specimens stored in VTM, which may not reflect the performance in fresh clinical samples. These samples were used because of the difficulty of obtaining fresh dry swabs intended for this POC test. Because VTM is irrelevant for immediate testing at the POC, we tested RNA extracted from clinical samples to assess the overall assay performance on clinical RNA and subsequently tested feasibility of direct amplification of VTM samples. Nevertheless, this testing shows excellent analytical reactivity to clinical specimens, no cross-reactivity with other respiratory pathogens, analytical sensitivity comparable to the CDC RT-PCR, and resilience in the presence of nasal matrix. While our assay is relatively fast and sensitive, there is potential to further improve the performance. We ran RT-LAMP at 64°C, which is 9°C above the optimal temperature for the RT enzyme. We hypothesize that cooling of the heat block when the lid is open, followed by a few minutes to ramp back up to 64°C, provided an opportunity for the RT enzyme to operate. In contrast, the reaction speed of the temperature-stable TFv1 DNA polymerase in this assay is optimal at higher temperature. Thus, operating the device with an initial 55°C step for optimal RT activity followed by a higher-temperature step for fast DNA amplification could increase sensitivity and/or speed.

The current design of RT-LAMP assay can be improved to include an IAC that is more representative of the SARS-CoV-2 virus. We currently use a DNA IAC, which has been used in several EUA assays, but the DNA control does not capture the potential failure of the lysis and complementary DNA conversion processes. We are developing a new IAC that uses RNA that can be packaged in a viral envelope. This modification may require redevelopment of excipients that preserve the RT-LAMP enzymes and the enveloped RNA.

POC tests like Harmony could provide rapid testing to enable the reopening of businesses, schools, and borders, but the need for POC testing extends beyond the current emergency. As natural immunity and vaccination take hold, COVID-19 is likely to become endemic with seasonal outbreaks. Tests such as Harmony will be necessary to help identify individuals with not only SARS-CoV-2 but also other respiratory pathogens such as influenza and Rous sarcoma virus, as the symptoms of these are similar. Before the current SARS-CoV-2 pandemic, the yearly influenza infections caused nearly 30 million cases in the United States alone, with 400,000 hospital admission and 35,000 deaths (*33*, *34*). In particular, when more typical patterns of respiratory infections occur, there will be a need for tests that can perform multiplex testing for individuals presenting respiratory symptoms. Tests such as Harmony will become a common practice for confirming presence of viral infections that can be treated with antiviral therapies or that demand infection control, as well as helping to reduce inappropriate use of antibiotics. Furthermore, test platforms developed for this pandemic could be more readily adapted to detect new diseases to increase readiness for future pandemics. The advances in awareness and acceptance of testing for infections at scale and the technology platforms developed will have an ongoing benefit for COVID-19 control, reducing harm from other endemic diseases and fighting future pandemics.

## MATERIALS AND METHODS

### Preparation of synthetic RNA standards

MERS and SARS-CoV-1 plasmid standards (10006623 and 10006624, Integrated DNA Technologies, Coralville, IA) were amplified using M13 PCR primers to generate DNA templates tagged with T7 promoter sequences. DNA templates containing T7 promoter sequences were transcribed using Hi-T7 RNA polymerase (M0658, New England Biolabs, Lawley, MA) following the manufacturer’s protocol, with ribonuclease (RNase) inhibitor (1 U/μl; N2611, Promega, Madison, WI) added. Following transcription, deoxyribonuclease I (0.01 U/μl; EN0521, Thermo Fisher Scientific, Waltham, MA) was added to each reaction and incubated at 37°C for 15 min. RNA transcripts were purified with the Monarch RNA Cleanup Kits (T2040, New England Biolabs). RNA was quantified on a Qubit 4 fluorometer (Q32852, RNA HS Assay Kit, Thermo Fisher Scientific, Waltham, MA) and checked for length and integrity by electrophoresis on a 2200 TapeStation (5067 RNA ScreenTape, Agilent Technologies, Santa Clara, CA). RNA at 10^10^ copies/μl was stored in nuclease-free water in single-use aliquots. SARS-CoV-2 RNA was prepared and quantified as previously described (*35*, *36*).

### In-house TFv1 polymerase production

#### Plasmid preparation

The chimeric polymerase (TFv1) was generated by assembly PCR using genomic material from *Thermus thermophilus* (*T. th*) HB27 (BAA-163D-5, American Type Culture Collection, Gaithersburg, MD) and a synthetic gene fragment derived from *Thermodesulfatator indicus* (*T. in*) (Integrated DNA Technologies, Coralville, IA), similar to the method previously described (*37*). Briefly, a 3′ terminal fragment of the *T. th* HB27 *polA* gene downstream of the finger domain was amplified by PCR using Phusion Hot Start Flex DNA Polymerase (M0535L, New England Biolabs) with a modified forward primer containing sequence overlap with the *T. in polA* gene fragment (*AA734-795*, codon optimized for *Escherichia coli*). A chimeric sequence was then generated by assembly PCR. A second *T. th* polymerase fragment, upstream of the finger domain and lacking the 3′ to 5′ exonuclease domains, was similarly amplified and appended to the 5′ end of the chimeric fragment. The resultant sequence corresponds to a *T. th* DNA polymerase I fragment, orthologous to the Stoffel fragment of *T. aq* polymerase, with a replacement in the finger domain derived from *T. in*. This gene was then cloned into the expression vector pET His6 TEV LIC cloning vector (2B-T) (a gift from S. Gradia, plasmid no. 29666, Addgene, Watertown, MA) by ligation-independent cloning (*38*). The annealed vector and insert were transformed into T7 Express lysY/Iq Competent *E. coli* (C2987H, New England Biolabs) per the manufacturer’s protocol and plated on LB carbenicillin plates (100 μg/ml). Colony plasmid inserts were verified by Sanger sequencing.

#### Protein expression and purification

Starter culture (50 ml) in LB with carbenicillin (100 μg/ml) was inoculated with a sequence-verified colony and incubated at 30°C overnight in a shaking incubator at 200 rpm. The culture was centrifuged at 5000 relative centrifugal force (RCF) for 15 min and resuspended in an equivalent volume fresh LB carbenicillin media. Flasks of 500 ml of LB with carbenicillin (100 μg/ml) were inoculated with 5 ml each of resuspended starter culture and incubated at 37°C in a shaking incubator at 200 rpm until optical density at 600 nm of 0.4 to 0.6 had been achieved. Cultures were induced with isopropyl-β-d-thiogalactopyranoside (0.4 mg/ml), incubated for 2 hours, then harvested by centrifugation at 5000 RCF for 15 min, decanted, and stored as cell pellets at −80°C until needed. Before purification, cell pellets were resuspended in lysis buffer [20 mM sodium phosphate (pH 7.4), 80 mM NaCl, 1% (v/v) Triton X-100 (648466, Sigma-Aldrich, St. Louis, MO), and lysozyme (1 mg/ml) from chicken egg white (MilliporeSigma, Burlington, MA)]. Suspensions were heated at 37°C for 20 min and then lysed by ultrasonic disruption using a Fisherbrand Model 120 Sonic Dismembrator (Thermo Fisher Scientific, Waltham, MA) with two cycles of 2-s pulses at 50% amplitude for 2 min while on ice. Lysates were heat-clarified at 65°C for 20 min, followed by centrifugation at 20,000 RCF. The supernatant was collected and diluted with sample diluent buffer [20 mM sodium phosphate, 1.8 M NaCI (pH 7.4) for 6× stock; five parts supernatant to 1 part diluent buffer] and filtered with a 0.22-μm syringe filter (25-342 Olympus, Genesee Scientific, San Diego, CA). The purified clarified lysate was applied to a 1-ml Hitrap Talon Crude column (Cytiva, Marlborough, MA) and enriched per the manufacturer’s default recommended specifications using an ÄKTA start system with the Frac30 fraction collector (Cytiva). Elution fractions were evaluated by A_280_ on a spectrophotometer (2200 NanoDrop, Thermo Fisher Scientific) using the protein’s expected molecular weight (63.062 kDa) and extinction coefficient (60,850 m^2^/mol) of the His-tagged polymerase. Elution fractions containing protein were diluted 1:10 with 10 mM sodium phosphate and applied to a 1-ml HiTrap Heparin HP column and purified per the manufacturer’s recommended specifications except for a modified binding buffer (10 mM sodium phosphate and 30 mM NaCl at pH 7). Fractions containing protein were concentrated and buffer-exchanged into storage buffer [17 mM tris-HCl, 167 mM KCl, and 17% glycerol (pH 7.5)] using an Amicon Ultra-2 30K centrifugal filter (MilliporeSigma), as recommended by the manufacturer. The recovered polymerase solution was then adjusted to 10 mM tris-HCl, 100 mM KCl, 2 mM dithiothreitol (DTT), 0.1% Triton X-100, 50% glycerol (pH 7.5). Polymerase purity was evaluated by SDS–polyacrylamide gel electrophoresis and spectrophotometry throughout purification.

### RT-LAMP assay design

Alignment of SARS-CoV-2 sequences (available on National Center for Biotechnology Information up to January 2020) were performed using Geneious (Auckland, New Zealand). Three sets of LAMP primers (Harmony NC1, Harmony NC2, and Harmony NC3) were developed targeting the 22,285 to 28,517 (232 bases), 28,528 to 28,471 (213 bases), and 29,135 to 29,416 (281 bases) regions of the SARS-CoV-2 nucleocapsid phosphoprotein gene. In silico analysis showed that this primer design matched 100% of SARS-CoV-2 sequences and did not have cross-reactivity to MERS and SARS-CoV-1. We used two detection probes, each composed of a fluorescent probe and its complementary quencher probe. For detection of SARS-CoV-2 RNA amplification, we used FAM-labeled detection probe and Iowa Black quencher probe. For detection of IAC amplification, we used TEX 615–labeled detection probe and BHQ-2–labeled quencher probe. The primer and probe sequences are listed in table S2.

### Wet RT-LAMP reactions

Characterization of polymerases (Fig. 2) was conducted in the wet reaction format. Water (2 μl) or SARS-CoV-2 RNA at 200 or 2000 copies/μl was added to 38 μl of master mix to achieve a 40-μl RT-LAMP reaction. Harmony NC1, NC2, and NC3 primer mix (20×) for SARS-CoV-2 amplification each contained 20 μM each forward inner primers/backward inner primers (FIP/BIP primer), 10 μM each loop primer, and 4 μM each F3/B3 primer. IAC primer stock (20×) contained 10 μM loop primer. Probe/quencher mix (20×) for SARS-CoV-2 or IAC amplification contained 4 μM fluorophore probes and 6 μM quencher probes. Depending on the experimental design, each RT-LAMP reaction contained 1× each primer mix or only 1× NC1 primer mix, 1× each probe or only 1× FAM probe, 2.5% (v/v) mannitol (OPS Diagnostics, EXMN 500-01), 5 mM DTT, WarmStart RTx (0.6 U/μl), 0.7 μg of TFv1 DNA polymerase or Warmstart DNA polymerase 2.0 (0.32 U/μl) (M 0538, New England Biolabs), 1.4 mM each deoxynucleotide triphosphate (dNTP), RNase inhibitor (1 U/μl; N2615, Promega), TiPP (25 mU/μl; M0296L, New England Biolabs), IAC DNA (250 copies/μl), 6 mM magnesium sulfate, and 1× ThermoPol buffer (B9004S, New England Biolabs) in nuclease-free water. RT-LAMP reactions were incubated at 63.3°C and FAM, and/or Texas Red signal was read every 13 s for 1 hour using a CFX96 thermal cycler (Bio-Rad).

### Lyophilization of RT-LAMP reagents

Reactions containing 2× primers (2 μM each FIP/BIP primer, 1 μM each loop primer, and 0.4 μM each F3/B3 primer) and probes (0.4 μM fluorophore probes and 0.6 μM quencher probes) in the presence of 5% (v/v) mannitol, 10 mM DTT, 1.4 μg of TFv1 DNA polymerase, WarmStart RTx (1.2 U/μl), 2.8 mM of each dNTP in 10 mM tris-HCl (pH 7.4), RNase inhibitor (2 U/μl), TiPP (50 mU/μl), and IAC DNA (500 copies/μl) were prepared in 20-μl aliquots of 0.2-ml reagent tubes and lyophilized. Packages were stored in desiccant packets at −20°C until use.

### Harmony RT-LAMP reaction setup

Lyophilized reagents were resuspended in a final volume of 40 μl containing 1× rehydration buffer [1× ThermoPol buffer (B9004S, New England Biolabs), 6 mM supplemental MgSO_4_, and 0.5% (v/v) Triton X-100]. For analytical sensitivity experiments, 2 μl of SARS-CoV-2 RNA in water was added. For clinical testing, 10 μl of extracted RNA was added to each RT-LAMP reaction. For testing the XPRIZE contrived specimens or VTM in direct-to-amplification experiments, 10 μl of sample (not extracted) was added to each RT-LAMP reaction. For usability testing, 40 μl of swab eluate was added directly to the RT-LAMP reaction using transfer pipettes or the in-house unified dispensers.

### Analytical sensitivity of RT-LAMP assay

Analytical sensitivity of lyophilized RT-LAMP was tested using purified RNA (0 × 10^6^ to 2 × 10^6^ copies/40 μl of reaction). We also tested the effect of salt, human DNA, and mucin on amplification of SARS-CoV-2 RNA and IAC DNA using simulated nasal matrix as previously described (*39*).

### Preparation of contrived swab samples and kits for usability testing

For the transfer pipette workflow, each kit contained one swab (either positive or negative), a vial (10-500-25, Thermo Fisher Scientific, Waltham, MA) containing 400 μl of 1× rehydration buffer (see the “Harmony RT-LAMP reaction setup” section), and one lyophilized RT-LAMP reagent tube. For positive swabs, 10 μl of SARS-CoV-2 DNA (100 copies/μl) in 0.05% Triton X-100 was pipetted onto the swab (1804-PF, Puritan Medical) and dried at room temperature in an air-clean chamber (825-PCR/HEPA, Plas-Labs, Lansing, NI) for 4 hours before packaging in a sealed foil pouch with desiccant (S-8032, ULINE). The unified sampler was built from parts using an assembly jig (custom order from Hygiena, LLC, Camarillo, CA). The unified dispenser contained a hollow-shaft polyurethane swab attached to a bulb preloaded with 400 μl of 1× rehydration buffer and a separate tube with a filter and drip dispenser friction fit inside the tube. Each custom sampler kit contained an in-house assembled dispenser unit and one lyophilized RT-LAMP reagent tube.

### Usability study

The Harmony usability study was approved by the Institutional Review Board (IRB) at the University of Washington (IRB no. STUDY00010884), and informed consent was provided by participants. HCWs (*n* = 10), including registered nurses, medical students, and dental students, were enrolled in this study over the course of 1 month. All data were collected without identifiers. The primary objective of this study was to determine the accuracy and precision of the two sample transfer methods and test whether variation in reproducibility affected assay performance, and the secondary objective was to gather user input on the feasibility of each protocol. Participants first received an explanation of the test and its intended use and then watched an instructional video narrated by the facilitator to become familiar with the Harmony workflow (movies S1 and S2). After watching the video, participants were given a packet with a unique identifier that contained comprehensive instructions for the study procedures and survey questions. Study procedures were modeled on a coaching methodology (*40*) to allow natural progression of the procedures and to identify blocking steps in the instructions. The facilitator sat 3 meters from the participant and kept a record of verbal questions and comments from participants but did not provide answers to procedural questions unless the participant could not proceed.

For part 1, data related to user preference on sample preparation, tube sizes, and their level of confidence in performing each task were collected anonymously as prompts within their study packet. Open-ended questions were extracted for themes in errors or issues and supplemented with comments and observations recorded by the facilitator. Samples were saved with deidentified labels, and after all participants had completed the study, facilitators measured volumes in each receiving tube from part 1. The variances of the volume recovered by each method were tested for their equality using *F* test, and the *P* value was reported. The means of the recovered volumes transferred by LPs or HCWs were compared using Student’s *t* test (two-sided), and the *P* value was reported.

For part 2, each participant followed instructions in the packet and on the integrated mobile phone to complete the entire Harmony workflow on four total contrived samples (two samples using the transfer pipette protocol and two using the unified sampler system). Kits were prepared as specified above.

The LP (*n =* 5) portion of the user testing aimed to compare performance of the Harmony device by HCW to that of an “experienced” population, by replicating this process with four students and a researcher from the Lutz Lab. Survey questions were omitted for this group because these volunteers provided feedback throughout the development of Harmony, and their views are included in the discussion of this manuscript. The accuracies of tests performed by LPs and HCWs using each method were tested for their equality using *Z* test (two-sided), and the *P* value was reported.

### Clinical nasal swab specimens from individuals presenting respiratory symptoms

A minimum sample size of 30 (each negative and positive) was required to achieve the CI of 90 to 100% (binomial cumulative distribution function) should all results be accurate. Our clinical specimen panel (*n* = 110) was previously used to evaluate other SARS-CoV-2 assays (*35*), but 9 specimens had insufficient volume for RNA extraction and were thus excluded from the study. These specimens were collected with informed consent as part of the Seattle Flu Study, approved by the IRB at the University of Washington (IRB no. STUDY0006181). Thermo Fisher Scientific’s OpenArray is an arrayed RT-qPCR system capable of detecting up to 26 pathogens simultaneously in a semiquantitative manner. Specimens from the Seattle Flu Study that had other respiratory pathogens detected using the OpenArray platform were used to determine cross reactivity with Harmony. All participant specimens were deidentified before the transfer to the Lutz Lab at the University of Washington for analysis. The remaining 101 specimens were evaluated using the newly extracted RNA for analysis by RT-qPCR and Harmony system, to avoid variations introduced from different batches of extraction. RNA was extracted from clinical nasal swab specimens stored in VTM using the QIAamp Viral RNA Mini Kit (52906, Qiagen, Hilden, Germany). Before extraction, 100 μl of each specimen was mixed with 40-μl negative VTM to reach the suggested 140-μl sample volume. RNA was extracted according to the manufacturer’s protocol, eluted into 70 μl of EB buffer, and stored at −80°C in single-use aliquots until amplification. RNA (5 μl) was used for analysis in 20 μl of RT-qPCR using U.S. CDC N1, N2, or RNA polymerase (RP) human control assays (*23*). RNA (10 μl) was used for analysis in 40 μl of lyophilized RT-LAMP in the Harmony workflow. Each sample was run in duplicate. Only samples with positive results from both Harmony replicates were reported as positive for SARS-CoV-2. Sensitivity [true positive/(true positive + false negative)], specificity [true negative/(true negative + false positive)], and concordance [(true positive + true negative)/(true positive + true negative + false positive + false negative)] were calculated and reported with 95% CI using binomial exact proportions.

### XPRIZE contrived sample panel

The blinded panel was assembled and distributed by HudsonAlpha Discovery (Huntsville, AL) for the COVID-19 XPRIZE competition, and information on the samples was revealed to the authors only after results were submitted to XPRIZE. Samples were shipped to the Lutz Lab at the University of Washington. We reported results for contrived samples of chemically inactivated SARS-CoV-2 viral particle (ZeptoMetrix Corporation, Buffalo, NY) spiked in 1× PBS (*n* = 29), nasal specimens (*n* = 20), or saliva (*n* = 20) at 0 to 1000 copies per reaction. All samples came in liquid form. We added 10 μl of sample and 30 μl of Harmony rehydration buffer to rehydrate each lyophilized reagent and tested across seven different Harmony devices. Each sample was originally tested once for each sample. To affirm the identity of these specimens, we also performed RT-qPCR using CDC primer/probes after the samples had been tested using the Harmony system. To improve our confidence in reporting sensitivity and specificity, we tested two additional replicates for each sample to achieve a total of triplicates.

### Housing

The housing combines the heater/reader assembly with the cell phone while concealing the connector cables and presenting a sturdy and easy-to-use platform. The three-dimensional (3D) CAD (computer-aided drafting) program, Fusion 360 (AutoDesk), was used for the design of housing. Prototypes were printed in polylactic acid (PLA) plastic (Overture Filament, OVPLA175) using a Prusa i3 MK3s at 0.3-mm layer height. Power was supplied to both the heater/reader assembly and the phone simultaneously using a Charge-Plus USB-C (LAVA Computer Manufacturing Inc.) connector device and a standard fast-charging American cellphone charger. The LAVA Charge-Plus USB-C device also facilitates the transfer of data between the devices.

### Reader/heater device

The aluminum block to house the reagent tubes was custom-made by B. Willman according to the specifications in fig. S3. The circuit boards were designed using Autodesk EAGLE (San Rafael, CA), and the assembly of the circuit boards is shown in figs. S4 and S5. The lid, latch, and upper and lower housing of the device were drawn using SolidWorks (Dassault Systèmes, Waltham, MA) and printed by Xometry - HP MultiJet Fusion 3D (Gaithersburg, MD). All parts were assembled as shown in fig. S6.

### Real-time fluorescence signal analysis

After the user inserts the reaction tube into the device, the software adjusts the temperature to reequilibrate the temperature of heat block back to the reaction temperature. During this initial period, the signals from the photo diodes [the blue LED (FAM signal for target amplification) and the yellow LED (TEX 615 signal for the IAC amplification)] are collected but not used in analyses. Once the initial period is over, signal analysis commences. Each signal is analyzed in real time by comparing the current mean of the signal over the most recent 60-s interval to the signal’s behavior some 360 s in the past. Specifically, the current mean is compared to a bound consisting of the signal’s mean over the period from 420 to 360 s in the past plus a multiple (1.9× for target and 1.5× for IAC) of the signal’s sample SD over that same period. If the current mean exceeds the bound and maintains that condition for at least 15 s, then the signal is said to have “indicated.” If the signal from the target emission indicates, then the presence of SARS-CoV-2 has been detected, and the software immediately reports “positive COVID-19.” If the signal from the IAC emission indicates, then successful amplification of the IAC has been detected. Then, if the target emission does not also indicate before the end of the run, then a “negative COVID-19” result is reported; this delay in reporting negative results provides the maximum opportunity to detect low concentrations of SARS-CoV-2 that may be present in samples. If neither the target emission nor the IAC emission is detected, then “test failed” is reported.

## Supplementary Materials

### This PDF file includes:

Supplementary Text Figs. S1 to S10 Tables S1 to S5 References

## Notes

View/request a protocol for this paper from *Bio-protocol*.

## References

[1] D. Cucinotta, M. Vanelli, WHO declares COVID-19 a pandemic. Acta Biomed. 91, 157–160 (2020). 32191675 10.23750/abm.v91i1.9397 PMC7569573

[2] World Health Organization, WHO Coronavirus (COVID-19) Dashboard (2021); https://covid19.who.int/.

[3] A. Sandford, Coronavirus: Half of humanity now on lockdown as 90 countries call for confinement (Euronews, 2021); www.euronews.com/2020/04/02/coronavirus-in-europe-spain-s-death-toll-hits-10-000-after-record-950-new-deaths-in-24-hou.

[4] G. Bonaccorsi, F. Pierri, M. Cinelli, A. Flori, A. Galeazzi, F. Porcelli, A. L. Schmidt, C. M. Valensise, A. Scala, W. Quattrociocchi, F. Pammolli, Economic and social consequences of human mobility restrictions under COVID-19. Proc. Natl. Acad. Sci. U.S.A. 117, 15530–15535 (2020). 32554604 10.1073/pnas.2007658117 PMC7355033

[5] R. A. Teran, K. A. Walblay, E. L. Shane, S. Xydis, S. Gretsch, A. Gagner, U. Samala, H. Choi, C. Zelinski, S. R. Black, Postvaccination SARS-CoV-2 infections among skilled nursing facility residents and staff members - Chicago, Illinois, December 2020-March 2021. MMWR Morb. Mortal. Wkly Rep. 70, 632–638 (2021). 33914721 10.15585/mmwr.mm7017e1 PMC8084122

[6] C. M. Brown, J. Vostok, H. Johnson, M. Burns, R. Gharpure, S. Sami, R. T. Sabo, N. Hall, A. Foreman, P. L. Schubert, G. R. Gallagher, T. Fink, L. C. Madoff, S. B. Gabriel, B. MacInnis, D. J. Park, K. J. Siddle, V. Harik, D. Arvidson, T. Brock-Fisher, M. Dunn, A. Kearns, A. S. Laney, Outbreak of SARS-CoV-2 Infections, Including COVID-19 Vaccine Breakthrough Infections, Associated with Large Public Gatherings — Barnstable County, Massachusetts, July 2021 (US CDC, 2021); www.cdc.gov/mmwr/volumes/70/wr/mm7031e2.htm?s_cid=mm7031e2_w#contribAff. 10.15585/mmwr.mm7031e2 PMC8367314 34351882

[7] Centers for Disease Control and Prevention, Requirement for Proof of Negative COVID-19 Test or Recovery from COVID-19 for All Air Passengers Arriving in the United States (2021); www.cdc.gov/coronavirus/2019-ncov/travelers/testing-international-air-travelers.html.

[8] E. C. Kline, N. Panpradist, I. T. Hull, Q. Wang, A. K. Oreskovic, P. D. Han, L. M. Starita, B. R. Lutz, Multiplex target-redundant RT-LAMP for robust detection of SARS-CoV-2 using novel fluorescent universal displacement probes. medRxiv 2021.08.13.21261995 (2021). 10.1128/spectrum.01583-21 PMC9430505 35708340

[9] V. L. Dao Thi, K. Herbst, K. Boerner, M. Meurer, L. P. M. Kremer, D. Kirrmaier, A. Freistaedter, D. Papagiannidis, C. Galmozzi, M. L. Stanifer, S. Boulant, S. Klein, P. Chlanda, D. Khalid, I. Barreto Miranda, P. Schnitzler, H. G. Kräusslich, M. Knop, S. Anders, A colorimetric RT-LAMP assay and LAMP-sequencing for detecting SARS-CoV-2 RNA in clinical samples. Sci. Transl. Med. 12, eabc7075 (2020). 32719001 10.1126/scitranslmed.abc7075 PMC7574920

[10] S. Wu, X. Liu, S. Ye, J. Liu, W. Zheng, X. Dong, X. Yin, Colorimetric isothermal nucleic acid detection of SARS-CoV-2 with dye combination. Heliyon 7, e06886 (2021). 33903853 10.1016/j.heliyon.2021.e06886 PMC8059943

[11] J. Xu, J. Wang, Z. Zhong, X. Su, K. Yang, Z. Chen, D. Zhang, T. Li, Y. Wang, S. Zhang, S. Ge, J. Zhang, N. Xia, Room-temperature-storable PCR mixes for SARS-CoV-2 detection. Clin. Biochem. 84, 73–78 (2020). 32592724 10.1016/j.clinbiochem.2020.06.013 PMC7313492

[12] N. Panpradist, I. A. Beck, J. Vrana, N. Higa, D. McIntyre, P. S. Ruth, I. So, E. C. Kline, R. Kanthula, A. Wong-On-Wing, J. Lim, D. Ko, R. Milne, T. Rossouw, U. D. Feucht, M. Chung, G. Jourdain, N. Ngo-Giang-Huong, L. Laomanit, J. Soria, J. Lai, E. D. Klavins, L. M. Frenkel, B. R. Lutz, OLA-Simple: A software-guided HIV-1 drug resistance test for low-resource laboratories. EBioMedicine 50, 34–44 (2019). 31767540 10.1016/j.ebiom.2019.11.002 PMC6921160

[13] N. Panpradist, I. A. Beck, P. S. Ruth, S. Ávila-Ríos, C. García-Morales, M. Soto-Nava, D. Tapia-Trejo, M. Matías-Florentino, H. E. Paz-Juarez, S. del Arenal-Sanchez, G. Reyes-Terán, B. R. Lutz, L. M. Frenkel, Near point-of-care, point-mutation test to detect drug resistance in HIV-1: A validation study in a Mexican cohort. AIDS 34, 1331–1338 (2020). 32205723 10.1097/QAD.0000000000002524 PMC8284934

[14] J. D. Vrana, N. Panpradist, N. Higa, D. Ko, P. Ruth, R. Kanthula, J. J. Lai, Y. Yang, S. R. Sakr, B. Chohan, M. H. Chung, L. M. Frenkel, B. R. Lutz, E. Klavins, I. A. Beck, Implementation of an interactive mobile application to pilot a rapid assay to detect HIV drug resistance mutations in Kenya. *medRxiv* (2021); https://www.medrxiv.org/content/10.1101/2021.05.06.21256654v1.full.pdf+html. 10.1371/journal.pgph.0000185 PMC10021139 36962187

[15] B. Moon, M. Jones, J. Valdez, Lyophilized beads containing mannitol (Cepheid, 2021); https://patents.google.com/patent/US20050069898A1/en.

[16] D.-C. Nyan, L. E. Ulitzky, N. Cehan, P. Williamson, V. Winkelman, M. Rios, D. R. Taylor, Rapid detection of hepatitis B virus in blood plasma by a specific and sensitive loop-mediated isothermal amplification assay. Clin. Infect. Dis. 59, 16–23 (2014). 24704724 10.1093/cid/ciu210 PMC4305128

[17] A. N. Spiess, N. Mueller, R. Ivell, Trehalose is a potent PCR enhancer: Lowering of DNA melting temperature and thermal stabilization of taq polymerase by the disaccharide trehalose. Clin. Chem. 50, 1256–1259 (2004). 15229160 10.1373/clinchem.2004.031336

[18] Y. Mori, K. Nagamine, N. Tomita, T. Notomi, Detection of loop-mediated isothermal amplification reaction by turbidity derived from magnesium pyrophosphate formation. Biochem. Biophys. Res. Commun. 289, 150–154 (2001). 11708792 10.1006/bbrc.2001.5921

[19] K. Tone, R. Fujisaki, T. Yamazaki, K. Makimura, Enhancing melting curve analysis for the discrimination of loop-mediated isothermal amplification products from four pathogenic molds: Use of inorganic pyrophosphatase and its effect in reducing the variance in melting temperature values. J. Microbiol. Methods 132, 41–45 (2017). 27984058 10.1016/j.mimet.2016.10.020

[20] S. Xie, Y. Yuan, Y. Chai, R. Yuan, Tracing phosphate ions generated during loop-mediated isothermal amplification for electrochemical detection of nosema bombycis genomic DNA PTP1. Anal. Chem. 87, 10268–10274 (2015). 26412581 10.1021/acs.analchem.5b01858

[21] I. New England Biolabs, Thermostable Inorganic Pyrophosphatase (2021); www.neb.com/products/m0296-thermostable-inorganic-pyrophosphatase#Product%20Information.

[22] T. Lennox, B. E. Slatko, L. E. Sears, Purified thermostable inorganic pyprophosphatase obtainable from thermococcus litoralis (New England Biolabs Inc., 2021); https://patents.google.com/patent/US5861296A/en.

[23] U.S. Centers for Disease Control and Prevention, CDC 2019-Novel Coronavirus (2019-nCoV) Real-Time RT-PCR Diagnostic Panel For Emergency Use Only (U.S. Food and Drug Administration, 2021); www.fda.gov/media/134922/download.

[24] Thermo Fisher Scientific, OpenArray Technology Overview (2021); www.thermofisher.com/us/en/home/life-science/pcr/real-time-pcr/real-time-openarray/open-array-technology.html.

[25] C. B. F. Vogels, A. E. Watkins, C. A. Harden, D. E. Brackney, J. Shafer, J. Wang, C. Caraballo, C. C. Kalinich, I. M. Ott, J. R. Fauver, E. Kudo, P. Lu, A. Venkataraman, M. Tokuyama, A. J. Moore, M. C. Muenker, A. Casanovas-Massana, J. Fournier, S. Bermejo, M. Campbell, R. Datta, A. Nelson; Yale IMPACT Research Team, C. S. Dela Cruz, A. I. Ko, A. Iwasaki, H. M. Krumholz, J. D. Matheus, P. Hui, C. Liu, S. F. Farhadian, R. Sikka, A. L. Wyllie, N. D. Grubaugh, SalivaDirect: A simplified and flexible platform to enhance SARS-CoV-2 testing capacity. Med 2, 263–280.e6 (2021). 33521748 10.1016/j.medj.2020.12.010 PMC7836249

[26] R. Wolfel, V. M. Corman, W. Guggemos, M. Seilmaier, S. Zange, M. A. Müller, D. Niemeyer, T. C. Jones, P. Vollmar, C. Rothe, M. Hoelscher, T. Bleicker, S. Brünink, J. Schneider, R. Ehmann, K. Zwirglmaier, C. Drosten, C. Wendtner, Virological assessment of hospitalized patients with COVID-2019. Nature 581, 465–469 (2020). 32235945 10.1038/s41586-020-2196-x

[27] J. Zhu, J. Guo, Y. Xu, X. Chen, Viral dynamics of SARS-CoV-2 in saliva from infected patients. J. Infect. 81, e48–e50 (2020). 32593658 10.1016/j.jinf.2020.06.059 PMC7316041

[28] B. Pang, J. Xu, Y. Liu, H. Peng, W. Feng, Y. Cao, J. Wu, H. Xiao, K. Pabbaraju, G. Tipples, M. A. Joyce, H. A. Saffran, D. L. Tyrrell, H. Zhang, X. C. Le, Isothermal amplification and ambient visualization in a single tube for the detection of SARS-CoV-2 using loop-mediated amplification and CRISPR technology. Anal. Chem. 92, 16204–16212 (2020). 33238709 10.1021/acs.analchem.0c04047

[29] P. Craw, W. Balachandran, Isothermal nucleic acid amplification technologies for point-of-care diagnostics: A critical review. Lab Chip 12, 2469–2486 (2012). 22592150 10.1039/c2lc40100b

[30] E. Gonzalez-Gonzalez, I. Montserrat Lara-Mayorga, I. P. Rodríguez-Sánchez, Y. S. Zhang, S. O. Martínez-Chapa, G. Trujillo-de Santiago, M. M. Alvarez, Colorimetric loop-mediated isothermal amplification (LAMP) for cost-effective and quantitative detection of SARS-CoV-2: The change in color in LAMP-based assays quantitatively correlates with viral copy number. Anal. Methods 13, 169–178 (2021). 33399137 10.1039/d0ay01658f

[31] J. Rodriguez-Manzano, K. Malpartida-Cardenas, N. Moser, I. Pennisi, M. Cavuto, L. Miglietta, A. Moniri, R. Penn, G. Satta, P. Randell, F. Davies, F. Bolt, W. Barclay, A. Holmes, P. Georgiou, Handheld point-of-care system for rapid detection of SARS-CoV-2 extracted RNA in under 20 min. ACS Cent. Sci. 7, 307–317 (2021). 33649735 10.1021/acscentsci.0c01288 PMC7839415

[32] Chai Bio (Santa Clara, CA).

[33] W. Putri, D. J. Muscatello, M. S. Stockwell, A. T. Newall, Economic burden of seasonal influenza in the United States. Vaccine 36, 3960–3966 (2018). 29801998 10.1016/j.vaccine.2018.05.057

[34] Centers for Diseases Control and Prevention, Estimated influenza illnesses, medical visits, hospitalizations, and deaths in the United States — 2019–2020 influenza season. (1 October 2021); https://www.cdc.gov/flu/about/burden/2019-2020.html.

[35] N. Panpradist, Q. Wang, P. S. Ruth, J. H. Kotnik, A. K. Oreskovic, A. Miller, S. W. A. Stewart, J. Vrana, P. D. Han, I. A. Beck, L. M. Starita, L. M. Frenkel, B. R. Lutz, Simpler and faster Covid-19 testing: Strategies to streamline SARS-CoV-2 molecular assays. EBioMedicine 64, 103236 (2021). 33582488 10.1016/j.ebiom.2021.103236 PMC7878117

[36] G. K. Gulati, N. Panpradist, S. W. A. Stewart, I. A. Beck, C. Boyce, A. K. Oreskovic, C. García-Morales, S. Avila-Ríos, P. D. Han, G. Reyes-Terán, L. M. Starita, L. M. Frenkel, B. R. Lutz, J. J. Lai, Inexpensive workflow for simultaneous monitoring of HIV viral load and detection of SARS-CoV-2 infection. *medRxiv* 2021.08.18.21256786 (2021).

[37] N. Morant, Novel thermostable DNA polymerases for isothermal DNA amplification. Thesis, University of Bath (2015).

[38] Addgene, Ligation Independent Cloning (2021); www.addgene.org/protocols/lic/.

[39] N. Panpradist, B. J. Toley, X. Zhang, S. Byrnes, J. R. Buser, J. A. Englund, B. R. Lutz, Swab sample transfer for point-of-care diagnostics: Characterization of swab types and manual agitation methods. PLOS ONE 9, e105786 (2014). 25181250 10.1371/journal.pone.0105786 PMC4152222

[40] R. Mack, J. B. Robinson, When novices elicit knowledge: Question asking in designing, evaluating, and learning to use software, in *The Psychology of Expertise: Cognitive Research and Empirical AI*, R. R. Hoffman, Ed. (Springer, 1992), pp. 245–268.

[41] FPbase, Spectra viewer (2020); www.fpbase.org/spectra/.

[42] Newport, Optical Filters (2021); www.newport.com/c/optical-filters.

[43] Mousers Electronics, LED lighting (2021); www.mouser.com/Optoelectronics/LED-Lighting/_/N-74g9t.

[44] A. Alekseenko, D. Barrett, Y. Pareja-Sanchez, R. J. Howard, E. Strandback, H. Ampah-Korsah, U. Rovšnik, S. Zuniga-Veliz, A. Klenov, J. Malloo, S. Ye, X. Liu, B. Reinius, S. J. Elsässer, T. Nyman, G. Sandh, X. Yin, V. Pelechano, Direct detection of SARS-CoV-2 using non-commercial RT-LAMP reagents on heat-inactivated samples. Sci. Rep. 11, 1820 (2021). 33469065 10.1038/s41598-020-80352-8 PMC7815738

[45] B. A. Rabe, C. Cepko, SARS-CoV-2 detection using isothermal amplification and a rapid, inexpensive protocol for sample inactivation and purification. Proc. Natl. Acad. Sci. U.S.A. 117, 24450–24458 (2020). 32900935 10.1073/pnas.2011221117 PMC7533677

[46] S. Wei, E. Kohl, A. Djandji, S. Morgan, S. Whittier, M. Mansukhani, E. Hod, M. D’Alton, Y. Suh, Z. Williams, Direct diagnostic testing of SARS-CoV-2 without the need for prior RNA extraction. Sci. Rep. 11, 2402 (2021). 33510181 10.1038/s41598-021-81487-y PMC7844049

[47] L. Bokelmann, O. Nickel, T. Maricic, S. Pääbo, M. Meyer, S. Borte, S. Riesenberg, Point-of-care bulk testing for SARS-CoV-2 by combining hybridization capture with improved colorimetric LAMP. Nat. Commun. 12, 1467 (2021). 33674580 10.1038/s41467-021-21627-0 PMC7935920

[48] W. Yamazaki, Y. Matsumura, U. Thongchankaew-Seo, Y. Yamazaki, M. Nagao, Development of a point-of-care test to detect SARS-CoV-2 from saliva which combines a simple RNA extraction method with colorimetric reverse transcription loop-mediated isothermal amplification detection. J. Clin. Virol. 136, 104760 (2021). 33610926 10.1016/j.jcv.2021.104760 PMC7877809

[49] J. Qian, S. A. Boswell, C. Chidley, Z. X. Lu, M. E. Pettit, B. L. Gaudio, J. M. Fajnzylber, R. T. Ingram, R. H. Ward, J. Z. Li, M. Springer, An enhanced isothermal amplification assay for viral detection. Nat. Commun. 11, 5920 (2020). 33219228 10.1038/s41467-020-19258-y PMC7679446

[50] M. Patchsung, K. Jantarug, A. Pattama, K. Aphicho, S. Suraritdechachai, P. Meesawat, K. Sappakhaw, N. Leelahakorn, T. Ruenkam, T. Wongsatit, N. Athipanyasilp, B. Eiamthong, B. Lakkanasirorat, T. Phoodokmai, N. Niljianskul, D. Pakotiprapha, S. Chanarat, A. Homchan, R. Tinikul, P. Kamutira, K. Phiwkaow, S. Soithongcharoen, C. Kantiwiriyawanitch, V. Pongsupasa, D. Trisrivirat, J. Jaroensuk, T. Wongnate, S. Maenpuen, P. Chaiyen, S. Kamnerdnakta, J. Swangsri, S. Chuthapisith, Y. Sirivatanauksorn, C. Chaimayo, R. Sutthent, W. Kantakamalakul, J. Joung, A. Ladha, X. Jin, J. S. Gootenberg, O. O. Abudayyeh, F. Zhang, N. Horthongkham, C. Uttamapinant, Clinical validation of a Cas13-based assay for the detection of SARS-CoV-2 RNA. Nat. Biomed. Eng. 4, 1140–1149 (2020). 32848209 10.1038/s41551-020-00603-x

[51] J. Joung, A. Ladha, M. Saito, M. Segel, R. Bruneau, M.-L. W. Huang, N.-G. Kim, X. Yu, J. Li, B. D. Walker, A. L. Greninger, K. R. Jerome, J. S. Gootenberg, O. O. Abudayyeh, F. Zhang, Point-of-care testing for COVID-19 using SHERLOCK diagnostics. medRxiv , 2020.05.04.20091231 (2020).

[52] J. P. Broughton, X. Deng, G. Yu, C. L. Fasching, V. Servellita, J. Singh, X. Miao, J. A. Streithorst, A. Granados, A. Sotomayor-Gonzalez, K. Zorn, A. Gopez, E. Hsu, W. Gu, S. Miller, C. Y. Pan, H. Guevara, D. A. Wadford, J. S. Chen, C. Y. Chiu, CRISPR-Cas12-based detection of SARS-CoV-2. Nat. Biotechnol. 38, 870–874 (2020). 32300245 10.1038/s41587-020-0513-4 PMC9107629

[53] Mammoth Biosciences Inc., INSTRUCTIONS FOR USE SARS-CoV-2 DETECTRTM Reagent Kit (2021); www.fda.gov/media/141765/download.

[54] UCSF Health Clinical Laboratories UCSF Clinical Labs at China Basin, SARS-CoV-2 RNA DETECTR Assay (2021); www.fda.gov/media/139937/download.

[55] MobileDetect Bio. Inc., MobileDetect-BIO BCC19 Test Kit for SARS-CoV-2 Detection (2021); www.fda.gov/media/141791/download.

[56] MobileDetect Bio. Inc. (2021), vol. 2021.

[57] SEASUN BIOMATERIALS Inc., AQ-TOP™ COVID-19 Rapid Detection Kit PLUS (2021); www.fda.gov/media/142800/download.

[58] Sherlock Biosciences Inc., INSTRUCTIONS FOR USE Sherlock^TM^ CRISPR SARS-CoV-2 kit (2021); www.fda.gov/media/137746/download.

[59] Color Health Inc., Color SARS-CoV-2 RT-LAMP Diagnostic Assay EUA Summary (2021); www.fda.gov/media/138249/download.

[60] Lucira Health, Lucira™ CHECK-IT COVID-19 Test Kit (2021); www.fda.gov/media/147494/download.

[61] D. MitraIvan, K. Dimov, J. R. Waldeisen, Colorimetric Detection of Nucleic Acid Amplification (2021); https://patents.google.com/patent/US20170044599A1/pt-pt.

