# Supplementary material for: Harmony COVID-19: A ready-to-use kit, low-cost detector, and smartphone app for point-of-care SARS-CoV-2 RNA detection
Source: Sci Adv. 2021 Dec 15;7(51):eabj1281. doi: 10.1126/sciadv.abj1281 (PMC8673764; doi:10.1126/sciadv.abj1281)
Supplement: Supplementary file 1 — Supplementary Text Figs. S1 to S10 Tables S1 to S5 References [file sciadv.abj1281_sm.pdf]

Science Advances
AAAS

Supplementary Materials for

Harmony COVID-19: A ready-to-use kit, low-cost detector, and smartphone app for point-of-care SARS-CoV-2 RNA detection

Nuttada Panpradist, Enos C. Kline, Robert G. Atkinson, Michael Roller, Qin Wang, Ian T. Hull, Jack H. Kotnik, Amy K. Oreskovic, Crissa Bennett, Daniel Leon, Victoria Lyon, Shane D. Gilligan-Steinberg, Peter D. Han, Paul K. Drain, Lea M. Starita, Matthew J. Thompson, Barry R. Lutz*

*Corresponding author. Email: blutz@uw.edu

Published 15 December 2021, *Sci. Adv.* **7**, eabj1281 (2021)
DOI: 10.1126/sciadv.abj1281

The PDF file includes:

Supplementary Text
Figs. S1 to S10
Tables S1 to S5
References

Other Supplementary Material for this manuscript includes the following:

Movies S1 and S2

Supplementary Text

Development of lyophilized RT-LAMP formulation

Trehalose can stabilize several enzymes, but we found that 2.5% (*w/v*) trehalose resulted in a slower reaction at 63°C, the optimal reaction temperature of the excipient-free RT-LAMP. An increase of reaction temperature to 65°C can speed up the trehalose-containing RT-LAMP reactions but resulted in unreliable detection at 20 RNA copies/reaction (**Fig. S1**).

A

Target (copies/ reaction)	min to detection							
	65 °C		63 °C		59 °C		55 °C	
	SARS-CoV-2	IAC	SARS-CoV-2	IAC	SARS-CoV-2	IAC	SARS-CoV-2	IAC
0	N/A	27.3, 27.7	N/A	31.8, 30.7	N/A	44.7, 42.6	N/A	N/A
20	N/A	29.8, 27.5	N/A, 31.63	30.6, 32.1	N/A, 43.0	42.0, 42.8	N/A	N/A, 60.4
200	N/A	27.3, 27.4	N/A, 27.86	29.4, 39.5	N/A, 43.2	47.9, 40.4	N/A	N/A, 62.0
2000	24.7, 28.6	N/A	28.0, 28.2	30.3, 32.8	N/A, 51.0	43.3, 41.8	N/A	N/A, 60.3

B

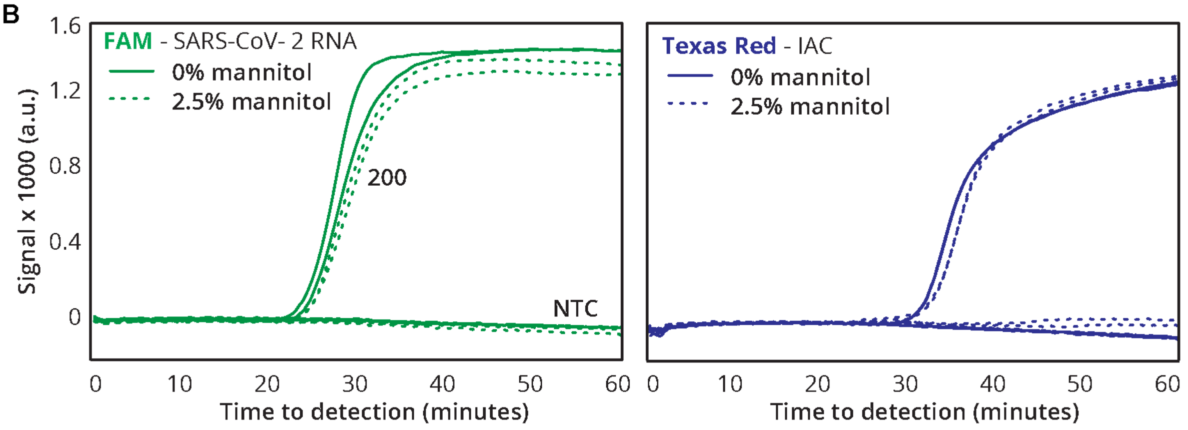

% mannitol	Target (copies/reaction)	SARS-CoV-2 (min to detection)	IAC (min to detection)
0	0	N/A	33.0, 33.0
	20	25.0, 25.7	N/A
2.5	0	N/A	33.9, 34.0
	20	26.3, 26.3	N/A

Fig. S1. Effects of excipients on fresh RT-LAMP reactions tested on a commercial real-time thermal cycler.

(**A**) Time to detection of SARS-CoV-2 RNA and IAC targets in the RT-LAMP containing 2.5% *(w/v)* trehalose and 0-2000 copies of SARS-CoV-2 RNA/40μL reaction. N/A means undetectable. For each condition, two technical replicates were run at 4 different reaction temperatures (i.e., 55°C, 59°C, 63°C, and 65°C). (**B**) Time to detection of SARS-CoV-2 RNA and IAC targets in the RT-LAMP reactions containing 0 or 20 copies of SARS-CoV-2 RNA/reaction, with and without 2.5% (*w*/*v*) mannitol, at 63°C.

non-SARS-CoV-2 RNA (copies/reaction)	SARS-CoV-2 RNA (copies/reaction)	min to detection SARS-CoV-2	IAC
4000 copies SARS-CoV-1	0	N/A	29.0, 30
4000 copies SARS-CoV-1	100	23.5, 24.1	N/A
4000 copies MERS	0	N/A	29.8, 30.1
4000 copies MERS	100	24.7, 24.7	N/A

Fig. S2. Cross-reactivity of MERS and SARS in lyophilized RT-LAMP.

Detection time (min) of SARS-CoV-2 RNA and IAC targets in the presence of 4000 copies of either SARS-CoV-1 RNA or MERS RNA in 40µL RT-LAMP reactions at 63°C. Two technical replicates were conducted in each condition. In the reactions without SARS-CoV-2 RNA, we did not observe amplification of FAM signal but observed IAC amplification indicating the amplification reaction was functioning properly in each replicate. 100 copies of SARS-CoV-2 RNA were amplified in each positive control replicate, indicating proper function of SARS-CoV-2 amplification in this master mix.

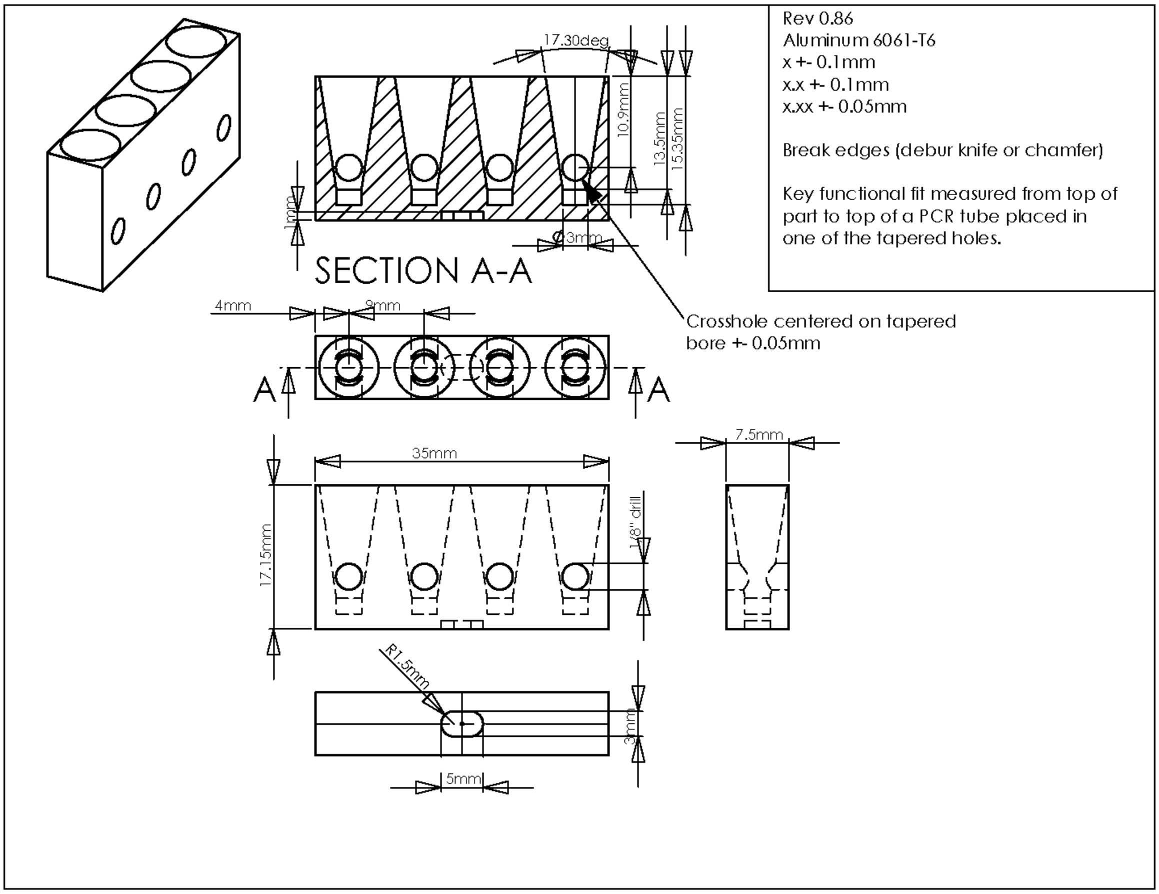

Fig. S3. Heat block used in Harmony COVID-19 device.

Drawing credit: Bryan Willman.

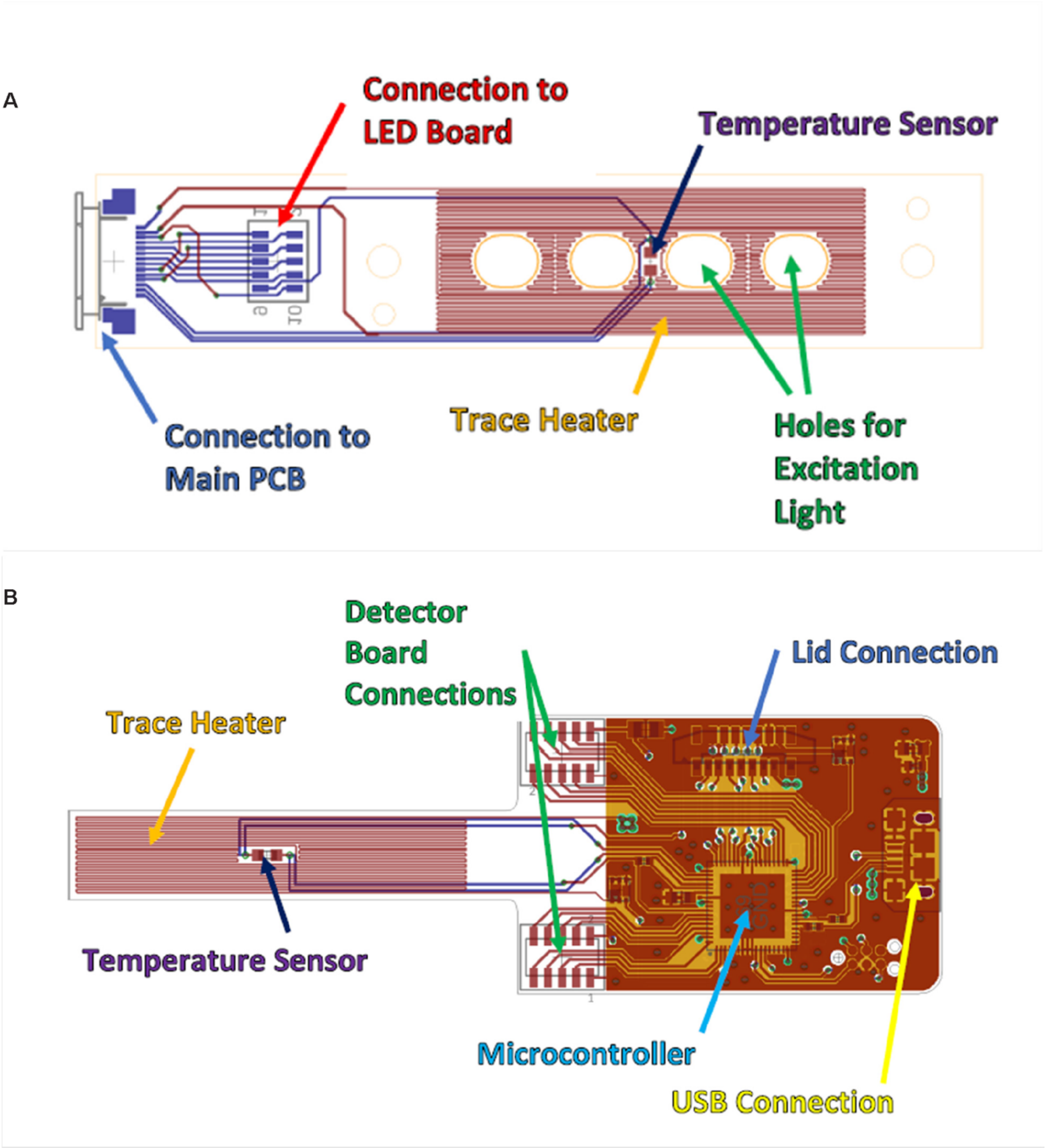

Fig. S4. The design of optical and heater circuit boards for the Harmony COVID-19 device. (**A**) LED board (**B**) the detector board

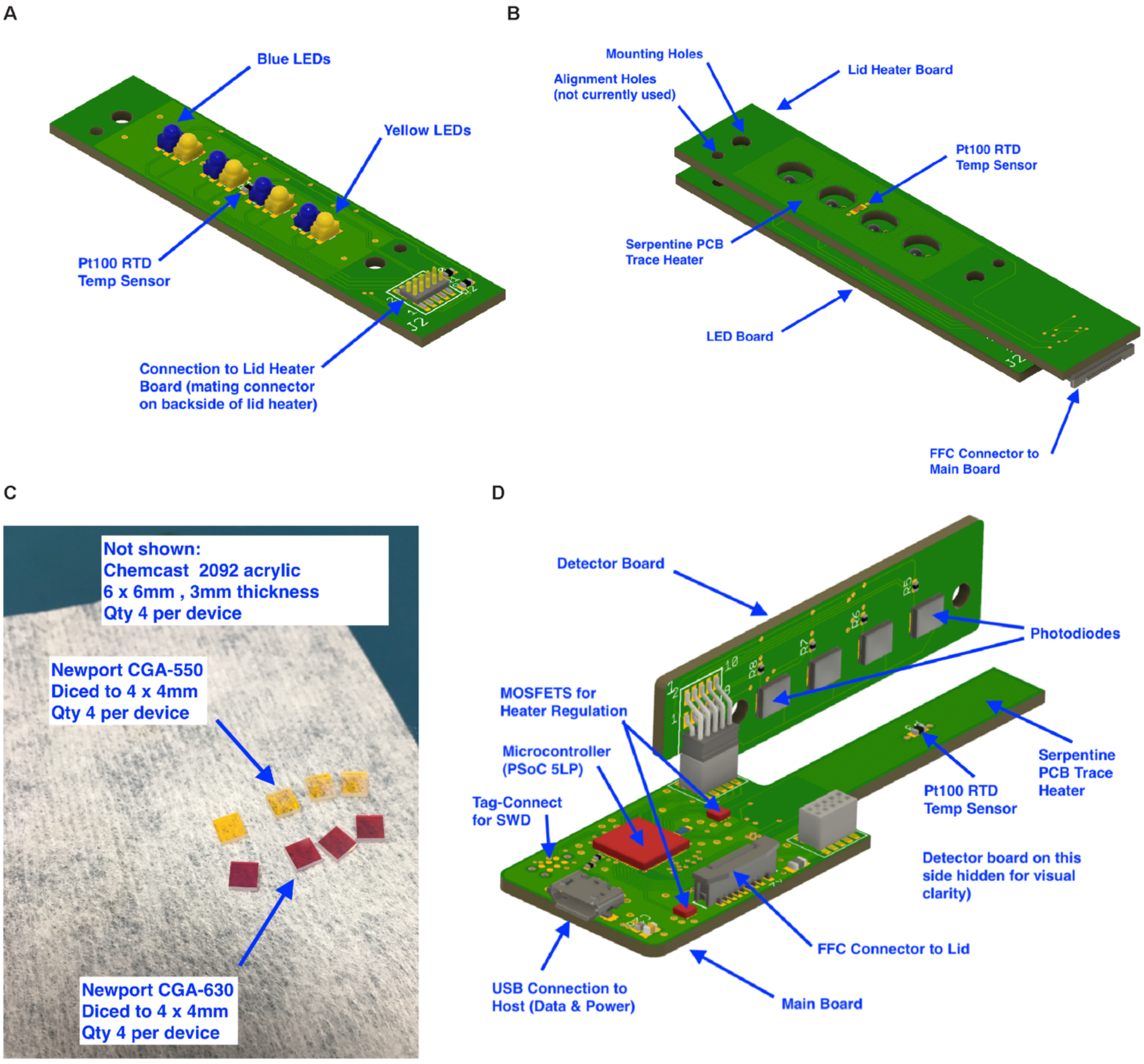

Fig. S5. Circuit board assembly and required parts.
(**A**) LED board. (**B**) LED board assembled to the lid heater board. (**C**) Custom-cut filter pieces. (**D**) Detector board and the main board. Each device require the following parts: thick film resistors (RCL12251R20FKEG, RCL12252R20FKEG, CRCW08050000Z0EBC and RCL12254R70FKEG from Vishey; RN73C1J75RBTDF from TE Connectivity; FTS-105-03-F-DV from Samtec; CR0402AJW-472GAS from Bourns), thin film resistors (RN73H1ETTP3003B25 from KOA Speer; ERJ-2RKF22R0X from Panasonic), multilayer ceramic capacitors (C0402C104M4RACAUTO, KEMET; 0603YC105KAT2A, AVX),

multilayer ceramic capacitor (GRM188R60J226MEA0D, Murata), USB connectors (10118193-0001LF and 10118192-0001LF, FCI / Amphenol), FFC & FPC connector (52745-1497, Molex), standard yellow LEDs (LY E63B-CBEA-26-1-Z, Osram Opto Semiconductor), standard blue LEDs (150141BS63140, Wurth Elektronik), board-to-board & mezzanine connector (CLP-105-02-F-D and FLE-105-01-G-DV-K-TR, Samtec), FFC/FPC jumper cables (15166-0143, Molex), FFC & FFC connector (52559-1452, Molex), 2 headers & wire Housings (FTSH-105-01-F-DH, Samtec), MOSFET (FDMA410NZ, ON Semiconductor), 2 board-mount temperature sensors (PTS060301B100RP100 from Vishay), ARM Microcontrollers (CY8C5888LTI-LP097, Cypress Semiconductor), Photodiodes (SFH 2201, Opto Semiconductor).

A

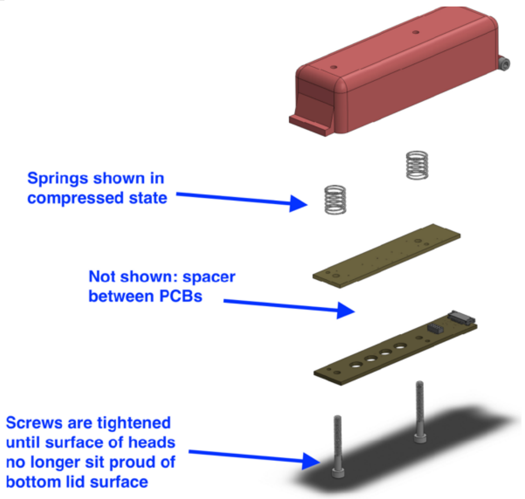

B

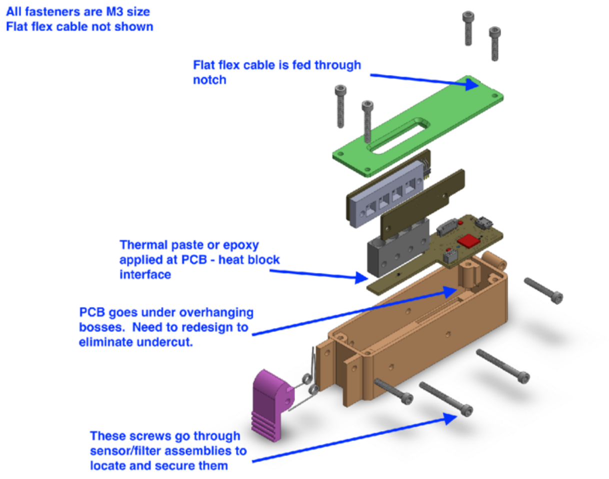

Fig. S6. Device assembly.
(**A**), Top part (**B**), Bottom part of the device are assembled using 10 screws, 2 compression springs, and 2 torsion springs (McMaster-Carr, Elmhurst, IL) including: Torsion Spring 90 Degree Angle, Left-Hand Wound, 0.204" OD (9271K109), Torsion Spring 90 Degree Angle, Right-Hand Wound, 0.204" OD (9271K143),Black-Oxide Alloy Steel Socket Head Screw M3 x 0.5 mm Thread, 5 mm Long (91290A110), Black-Oxide Alloy Steel Socket Head Screw M3 x 0.5 mm Thread, 10 mm Long (91290A115), Black-Oxide Alloy Steel Socket Head Screw M3 x 0.5 mm Thread, 15 mm Long (91290A572), Black-Oxide Alloy Steel Socket Head Screw M3 x 0.5 mm Thread, 20 mm Long (91290A123), Black-Oxide Alloy Steel Socket Head Screw M3 x 0.5 mm Thread, 25 mm Long (91290A125), Black-Oxide Alloy Steel Socket Head Screw M3 x 0.5 mm Thread, 30 mm Long, Partially Threaded (91290A130), Music-Wire Steel Compression Springs 0.875" Long, 0.36" OD, 0.296" ID (9434K73).

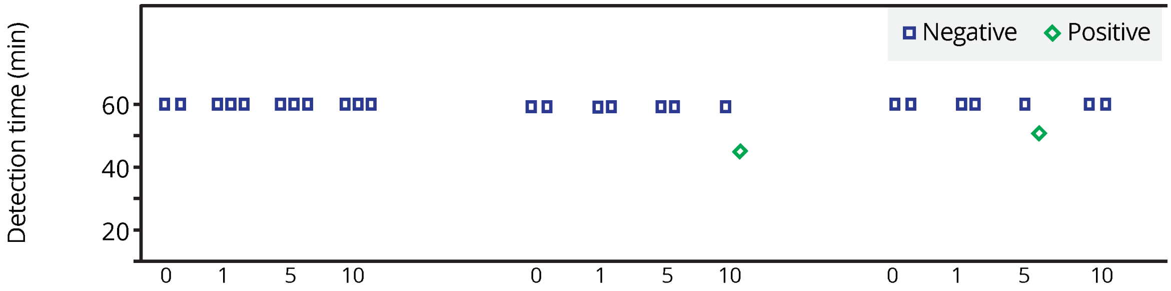

SARS-CoV-2 virus (copies/reaction)	SARS-CoV-2 virus in 1x PBS (copies/rxn)	SARS-CoV-2 virus in nasal specimens (copies/rxn)	SARS-CoV-2 virus in saliva (copies/rxn)
0	0/2	0/2	0/2
1	0/3	0/2	0/2
5	0/3	0/2	1/2
10	0/3	1/2	0/2

Fig. S7. Analysis of XPRIZE panel of contrived samples containing less than 20 copies/reaction.

A

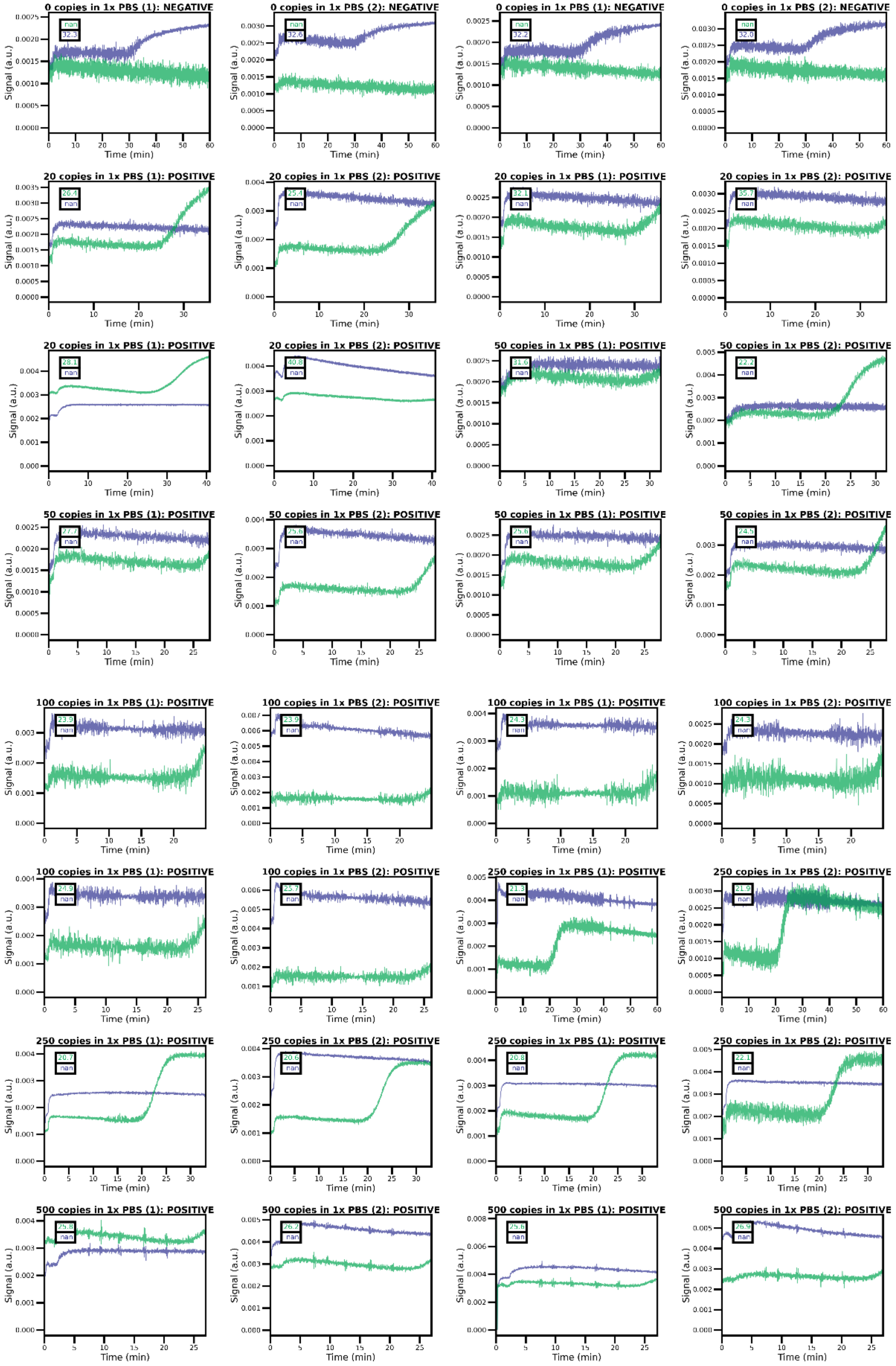
0 copies in 1x PBS (1): NEGATIVE
0 copies in 1x PBS (2): NEGATIVE
0 copies in 1x PBS (1): NEGATIVE
0 copies in 1x PBS (2): NEGATIVE
20 copies in 1x PBS (1): POSITIVE
20 copies in 1x PBS (2): POSITIVE
20 copies in 1x PBS (1): POSITIVE
20 copies in 1x PBS (2): POSITIVE
20 copies in 1x PBS (1): POSITIVE
20 copies in 1x PBS (2): POSITIVE
50 copies in 1x PBS (1): POSITIVE
50 copies in 1x PBS (2): POSITIVE
50 copies in 1x PBS (1): POSITIVE
50 copies in 1x PBS (2): POSITIVE
50 copies in 1x PBS (1): POSITIVE
50 copies in 1x PBS (2): POSITIVE
100 copies in 1x PBS (1): POSITIVE
100 copies in 1x PBS (2): POSITIVE
100 copies in 1x PBS (1): POSITIVE
100 copies in 1x PBS (2): POSITIVE
100 copies in 1x PBS (1): POSITIVE
100 copies in 1x PBS (2): POSITIVE
250 copies in 1x PBS (1): POSITIVE
250 copies in 1x PBS (2): POSITIVE
250 copies in 1x PBS (1): POSITIVE
250 copies in 1x PBS (2): POSITIVE
250 copies in 1x PBS (1): POSITIVE
250 copies in 1x PBS (2): POSITIVE
500 copies in 1x PBS (1): POSITIVE
500 copies in 1x PBS (2): POSITIVE
500 copies in 1x PBS (1): POSITIVE
500 copies in 1x PBS (2): POSITIVE
Signal (a.u.)
Time (min)

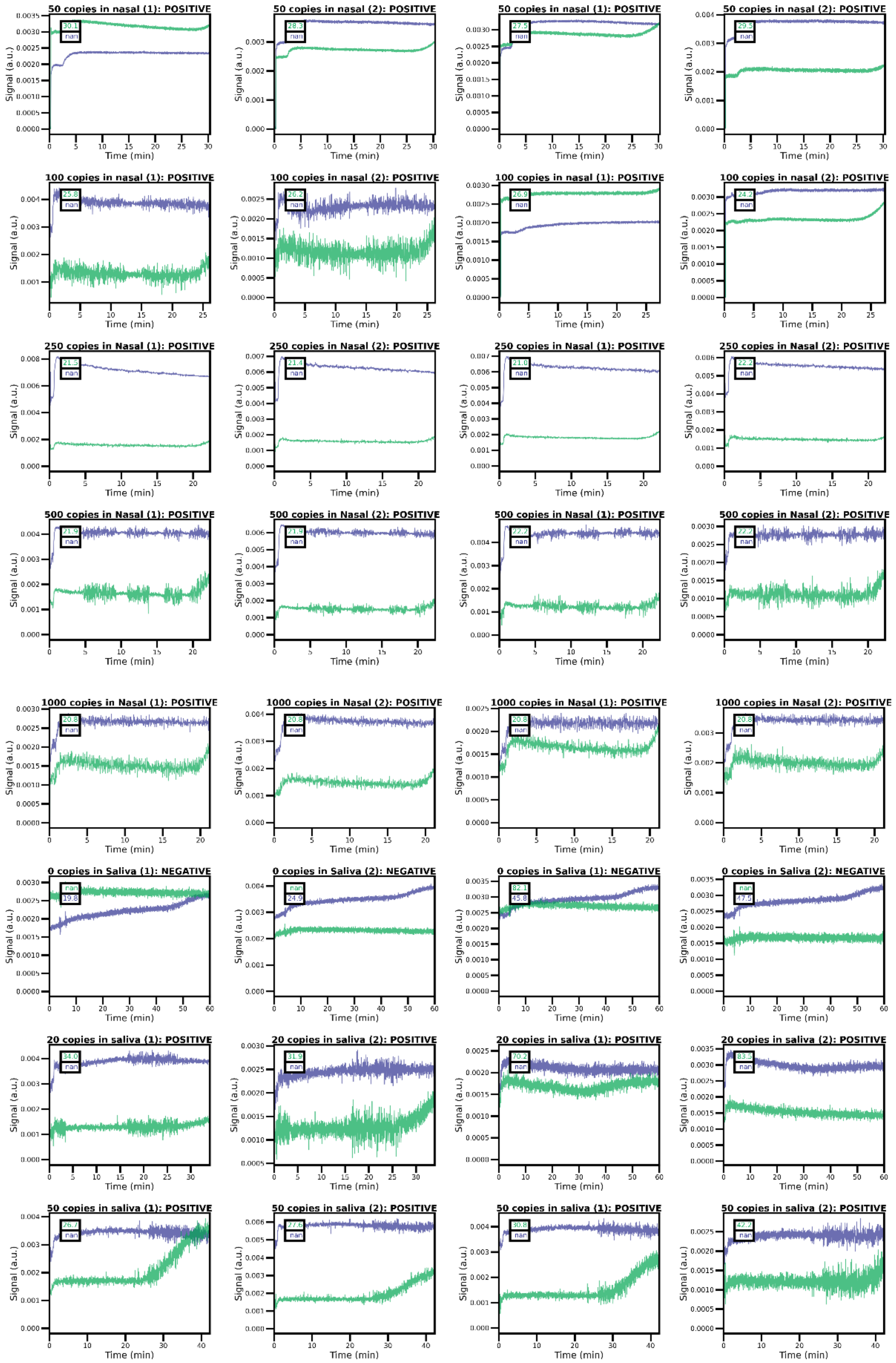
50 copies in nasal (1): POSITIVE
50 copies in nasal (2): POSITIVE
50 copies in nasal (1): POSITIVE
50 copies in nasal (2): POSITIVE
100 copies in nasal (1): POSITIVE
100 copies in nasal (2): POSITIVE
100 copies in nasal (1): POSITIVE
100 copies in nasal (2): POSITIVE
250 copies in Nasal (1): POSITIVE
250 copies in Nasal (2): POSITIVE
250 copies in Nasal (1): POSITIVE
250 copies in Nasal (2): POSITIVE
500 copies in Nasal (1): POSITIVE
500 copies in Nasal (2): POSITIVE
500 copies in Nasal (1): POSITIVE
500 copies in Nasal (2): POSITIVE
1000 copies in Nasal (1): POSITIVE
1000 copies in Nasal (2): POSITIVE
1000 copies in Nasal (1): POSITIVE
1000 copies in Nasal (2): POSITIVE
0 copies in Saliva (1): NEGATIVE
0 copies in Saliva (2): NEGATIVE
0 copies in Saliva (1): NEGATIVE
0 copies in Saliva (2): NEGATIVE
20 copies in saliva (1): POSITIVE
20 copies in saliva (2): POSITIVE
20 copies in saliva (1): POSITIVE
20 copies in saliva (2): POSITIVE
50 copies in saliva (1): POSITIVE
50 copies in saliva (2): POSITIVE
50 copies in saliva (1): POSITIVE
50 copies in saliva (2): POSITIVE
Signal (a.u.)
Time (min)

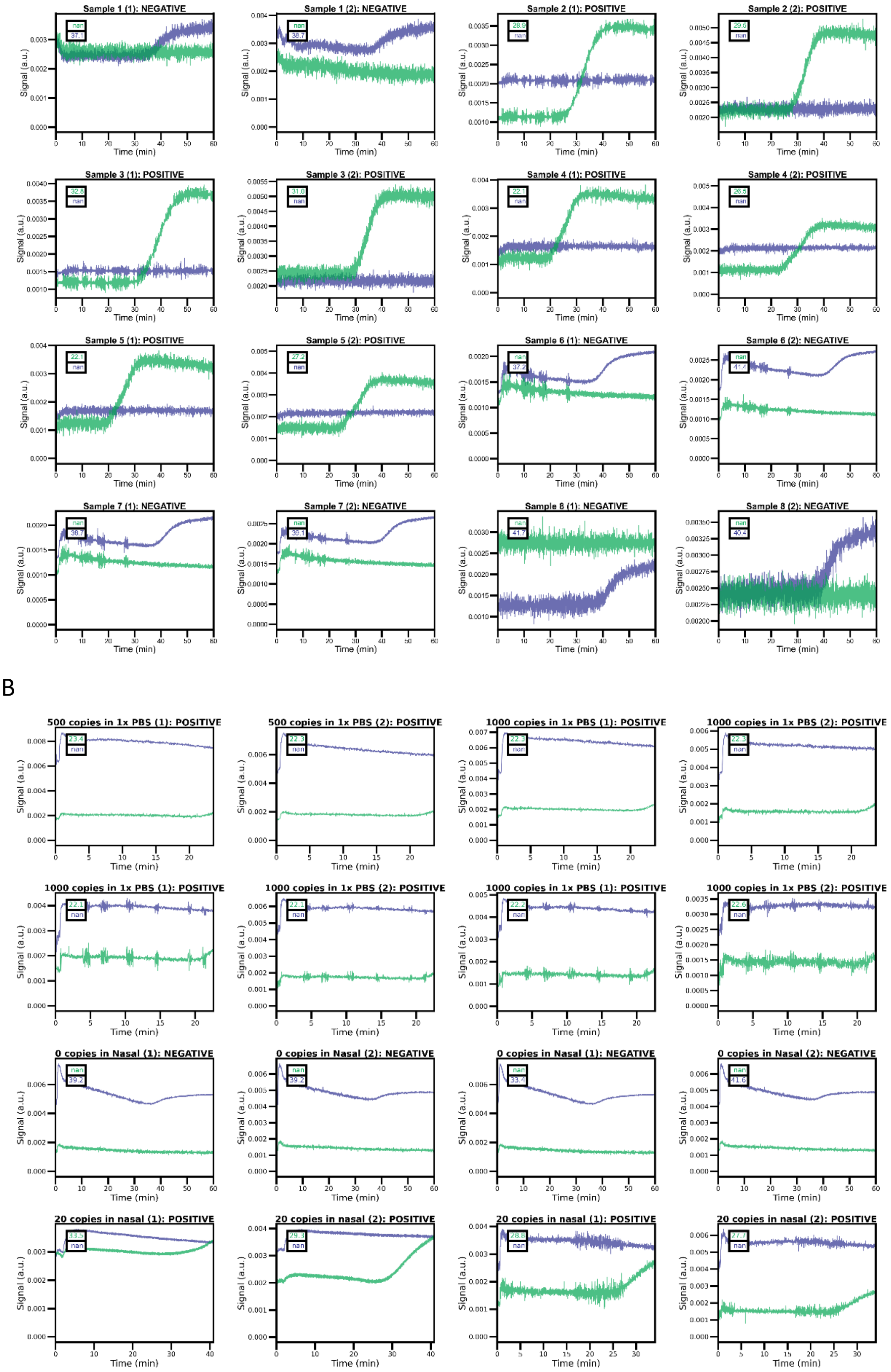
Sample 1 (1): NEGATIVE
Sample 1 (2): NEGATIVE
Sample 2 (1): POSITIVE
Sample 2 (2): POSITIVE
Sample 3 (1): POSITIVE
Sample 3 (2): POSITIVE
Sample 4 (1): POSITIVE
Sample 4 (2): POSITIVE
Sample 5 (1): POSITIVE
Sample 5 (2): POSITIVE
Sample 6 (1): NEGATIVE
Sample 6 (2): NEGATIVE
Sample 7 (1): NEGATIVE
Sample 7 (2): NEGATIVE
Sample 8 (1): NEGATIVE
Sample 8 (2): NEGATIVE
B
500 copies in 1x PBS (1): POSITIVE
500 copies in 1x PBS (2): POSITIVE
1000 copies in 1x PBS (1): POSITIVE
1000 copies in 1x PBS (2): POSITIVE
1000 copies in 1x PBS (1): POSITIVE
1000 copies in 1x PBS (2): POSITIVE
1000 copies in 1x PBS (1): POSITIVE
1000 copies in 1x PBS (2): POSITIVE
0 copies in Nasal (1): NEGATIVE
0 copies in Nasal (2): NEGATIVE
0 copies in Nasal (1): NEGATIVE
0 copies in Nasal (2): NEGATIVE
20 copies in nasal (1): POSITIVE
20 copies in nasal (2): POSITIVE
20 copies in nasal (1): POSITIVE
20 copies in nasal (2): POSITIVE
Signal (a.u.)
Time (min)

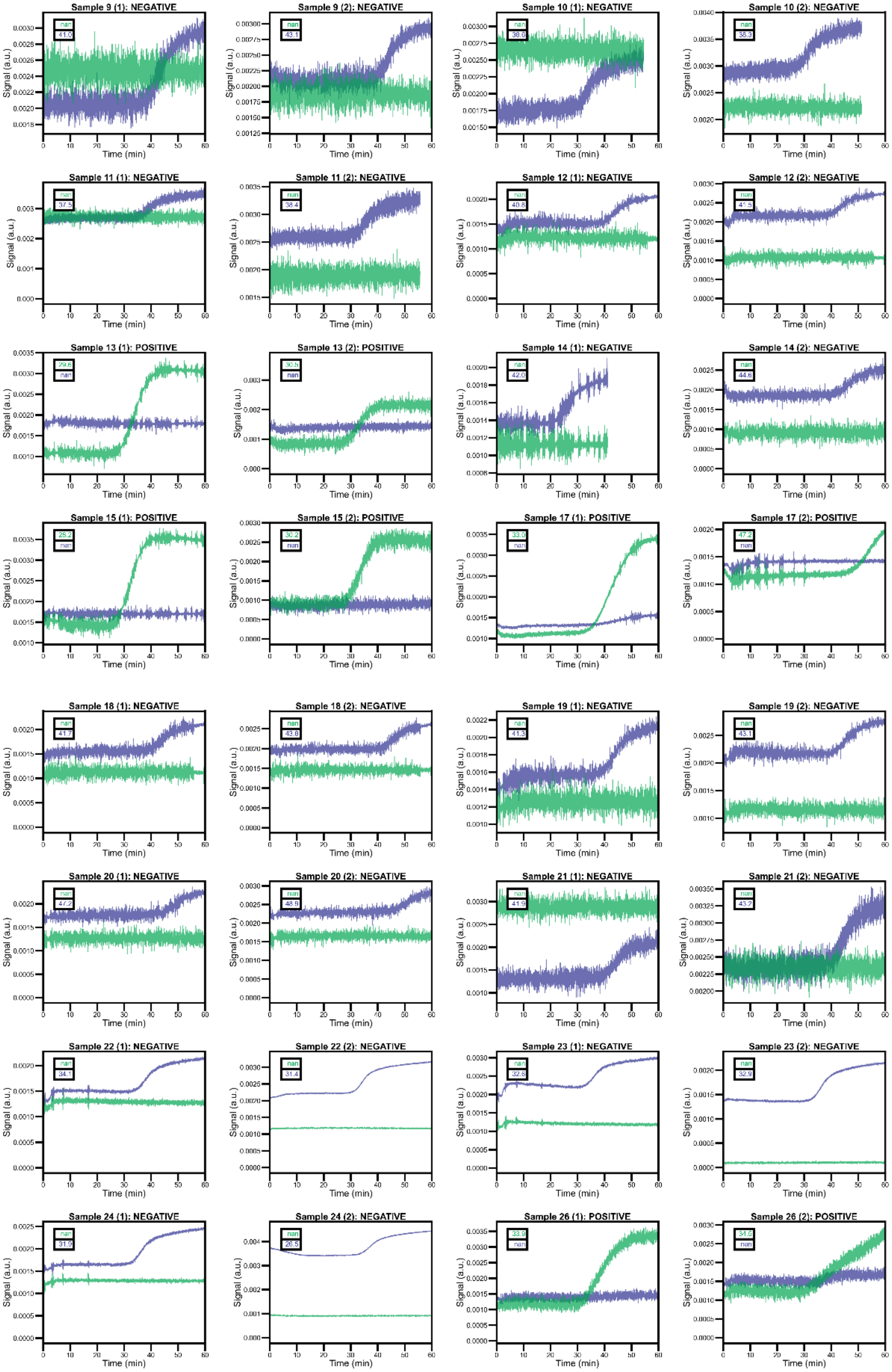
Sample 9 (1): NEGATIVE
Sample 9 (2): NEGATIVE
Sample 10 (1): NEGATIVE
Sample 10 (2): NEGATIVE
Sample 11 (1): NEGATIVE
Sample 11 (2): NEGATIVE
Sample 12 (1): NEGATIVE
Sample 12 (2): NEGATIVE
Sample 13 (1): POSITIVE
Sample 13 (2): POSITIVE
Sample 14 (1): NEGATIVE
Sample 14 (2): NEGATIVE
Sample 15 (1): POSITIVE
Sample 15 (2): POSITIVE
Sample 17 (1): POSITIVE
Sample 17 (2): POSITIVE
Sample 18 (1): NEGATIVE
Sample 18 (2): NEGATIVE
Sample 19 (1): NEGATIVE
Sample 19 (2): NEGATIVE
Sample 20 (1): NEGATIVE
Sample 20 (2): NEGATIVE
Sample 21 (1): NEGATIVE
Sample 21 (2): NEGATIVE
Sample 22 (1): NEGATIVE
Sample 22 (2): NEGATIVE
Sample 23 (1): NEGATIVE
Sample 23 (2): NEGATIVE
Sample 24 (1): NEGATIVE
Sample 24 (2): NEGATIVE
Sample 26 (1): POSITIVE
Sample 26 (2): POSITIVE
Signal (a.u.)
Time (min)

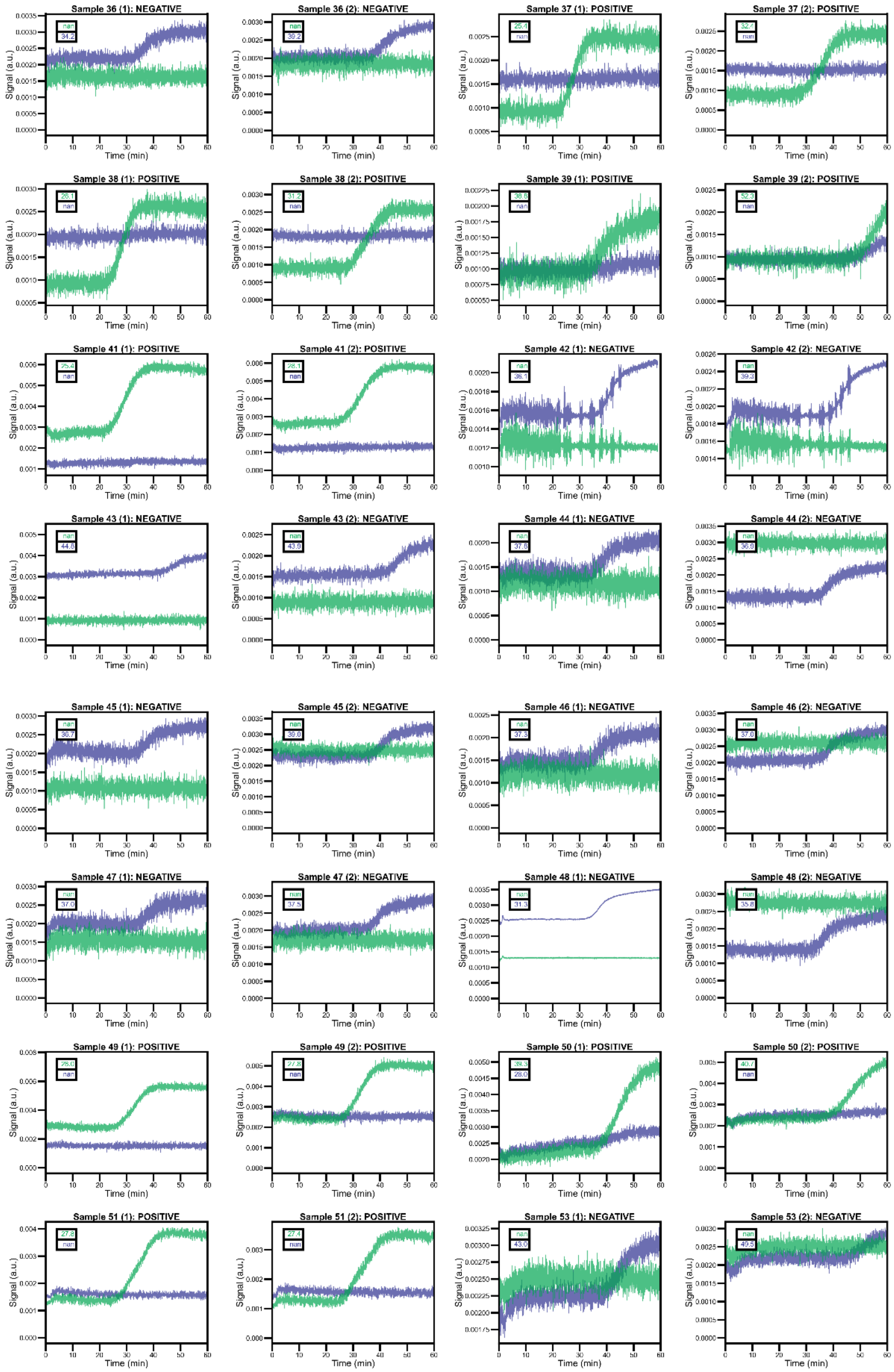
Sample 36 (1): NEGATIVE
Sample 36 (2): NEGATIVE
Sample 37 (1): POSITIVE
Sample 37 (2): POSITIVE
Sample 38 (1): POSITIVE
Sample 38 (2): POSITIVE
Sample 39 (1): POSITIVE
Sample 39 (2): POSITIVE
Sample 41 (1): POSITIVE
Sample 41 (2): POSITIVE
Sample 42 (1): NEGATIVE
Sample 42 (2): NEGATIVE
Sample 43 (1): NEGATIVE
Sample 43 (2): NEGATIVE
Sample 44 (1): NEGATIVE
Sample 44 (2): NEGATIVE
Sample 45 (1): NEGATIVE
Sample 45 (2): NEGATIVE
Sample 46 (1): NEGATIVE
Sample 46 (2): NEGATIVE
Sample 47 (1): NEGATIVE
Sample 47 (2): NEGATIVE
Sample 48 (1): NEGATIVE
Sample 48 (2): NEGATIVE
Sample 49 (1): POSITIVE
Sample 49 (2): POSITIVE
Sample 50 (1): POSITIVE
Sample 50 (2): POSITIVE
Sample 51 (1): POSITIVE
Sample 51 (2): POSITIVE
Sample 53 (1): NEGATIVE
Sample 53 (2): NEGATIVE
Signal (a.u.)
Time (min)

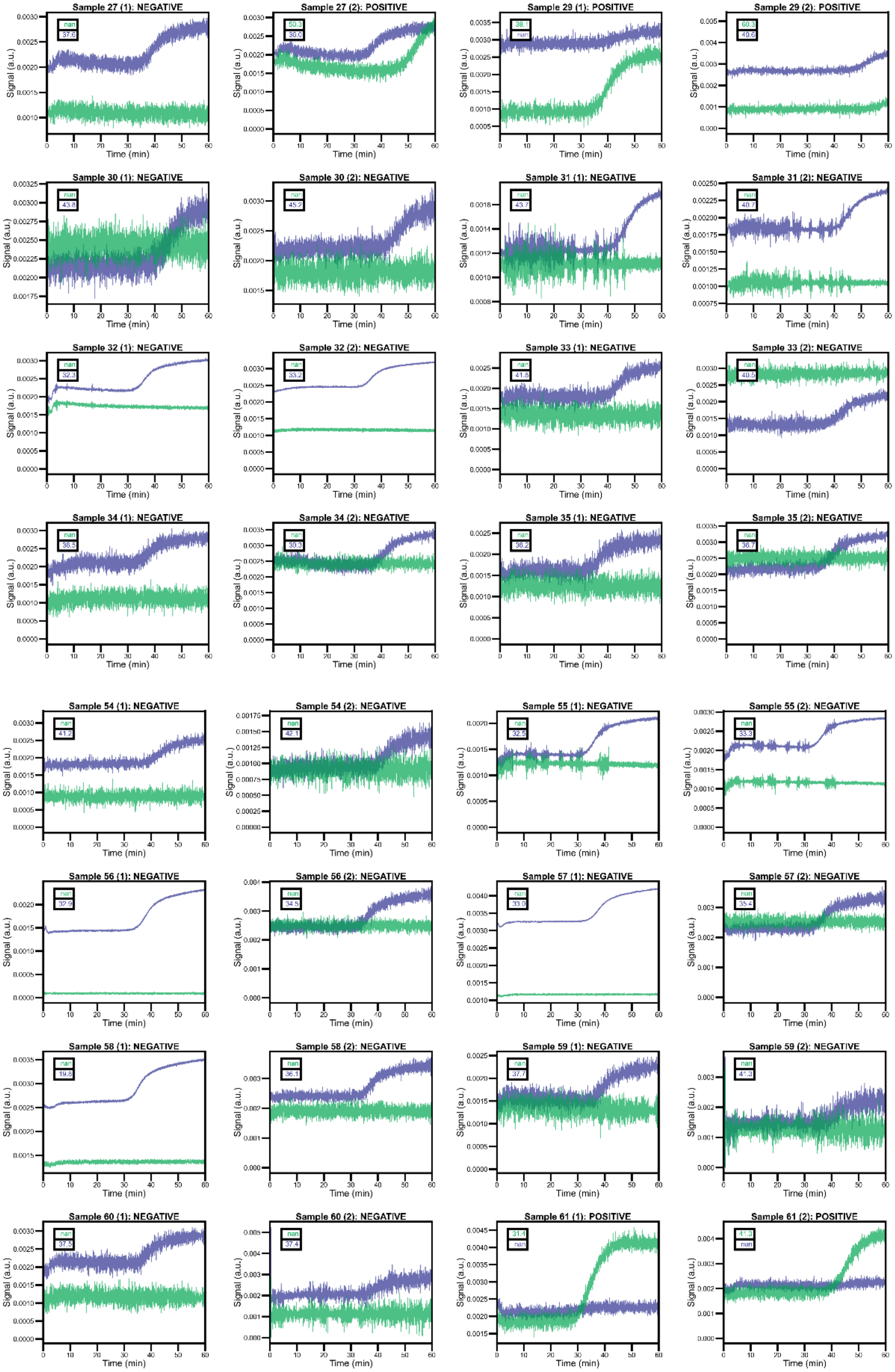
Sample 27 (1): NEGATIVE
Sample 27 (2): POSITIVE
Sample 29 (1): POSITIVE
Sample 29 (2): POSITIVE
Sample 30 (1): NEGATIVE
Sample 30 (2): NEGATIVE
Sample 31 (1): NEGATIVE
Sample 31 (2): NEGATIVE
Sample 32 (1): NEGATIVE
Sample 32 (2): NEGATIVE
Sample 33 (1): NEGATIVE
Sample 33 (2): NEGATIVE
Sample 34 (1): NEGATIVE
Sample 34 (2): NEGATIVE
Sample 35 (1): NEGATIVE
Sample 35 (2): NEGATIVE
Sample 54 (1): NEGATIVE
Sample 54 (2): NEGATIVE
Sample 55 (1): NEGATIVE
Sample 55 (2): NEGATIVE
Sample 56 (1): NEGATIVE
Sample 56 (2): NEGATIVE
Sample 57 (1): NEGATIVE
Sample 57 (2): NEGATIVE
Sample 58 (1): NEGATIVE
Sample 58 (2): NEGATIVE
Sample 59 (1): NEGATIVE
Sample 59 (2): NEGATIVE
Sample 60 (1): NEGATIVE
Sample 60 (2): NEGATIVE
Sample 61 (1): POSITIVE
Sample 61 (2): POSITIVE
Signal (a.u.)
Time (min)

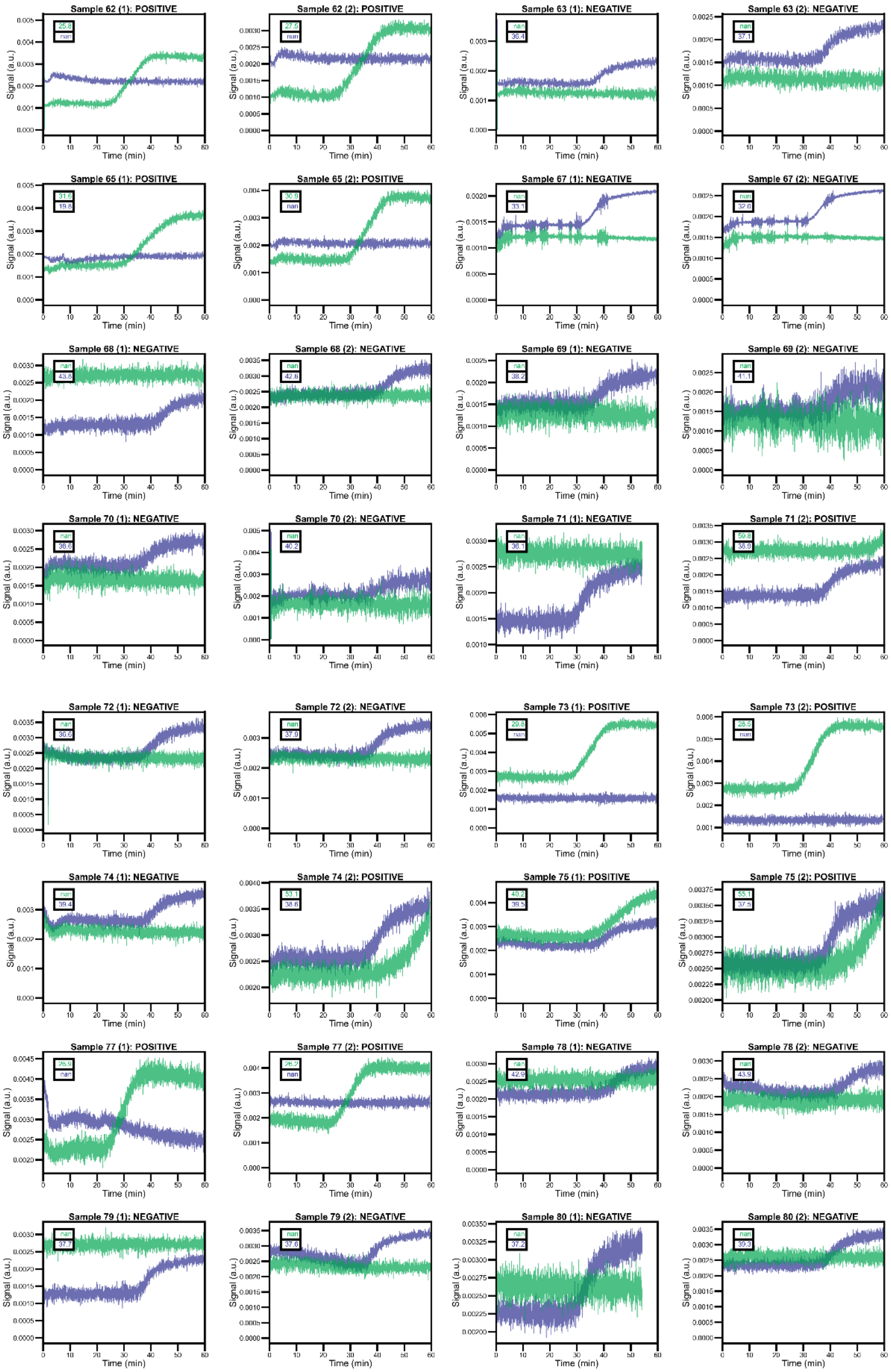
Sample 62 (1): POSITIVE
Sample 62 (2): POSITIVE
Sample 63 (1): NEGATIVE
Sample 63 (2): NEGATIVE
Sample 65 (1): POSITIVE
Sample 65 (2): POSITIVE
Sample 67 (1): NEGATIVE
Sample 67 (2): NEGATIVE
Sample 68 (1): NEGATIVE
Sample 68 (2): NEGATIVE
Sample 69 (1): NEGATIVE
Sample 69 (2): NEGATIVE
Sample 70 (1): NEGATIVE
Sample 70 (2): NEGATIVE
Sample 71 (1): NEGATIVE
Sample 71 (2): POSITIVE
Sample 72 (1): NEGATIVE
Sample 72 (2): NEGATIVE
Sample 73 (1): POSITIVE
Sample 73 (2): POSITIVE
Sample 74 (1): NEGATIVE
Sample 74 (2): POSITIVE
Sample 75 (1): POSITIVE
Sample 75 (2): POSITIVE
Sample 77 (1): POSITIVE
Sample 77 (2): POSITIVE
Sample 78 (1): NEGATIVE
Sample 78 (2): NEGATIVE
Sample 79 (1): NEGATIVE
Sample 79 (2): NEGATIVE
Sample 80 (1): NEGATIVE
Sample 80 (2): NEGATIVE
Signal (a.u.)
Time (min)

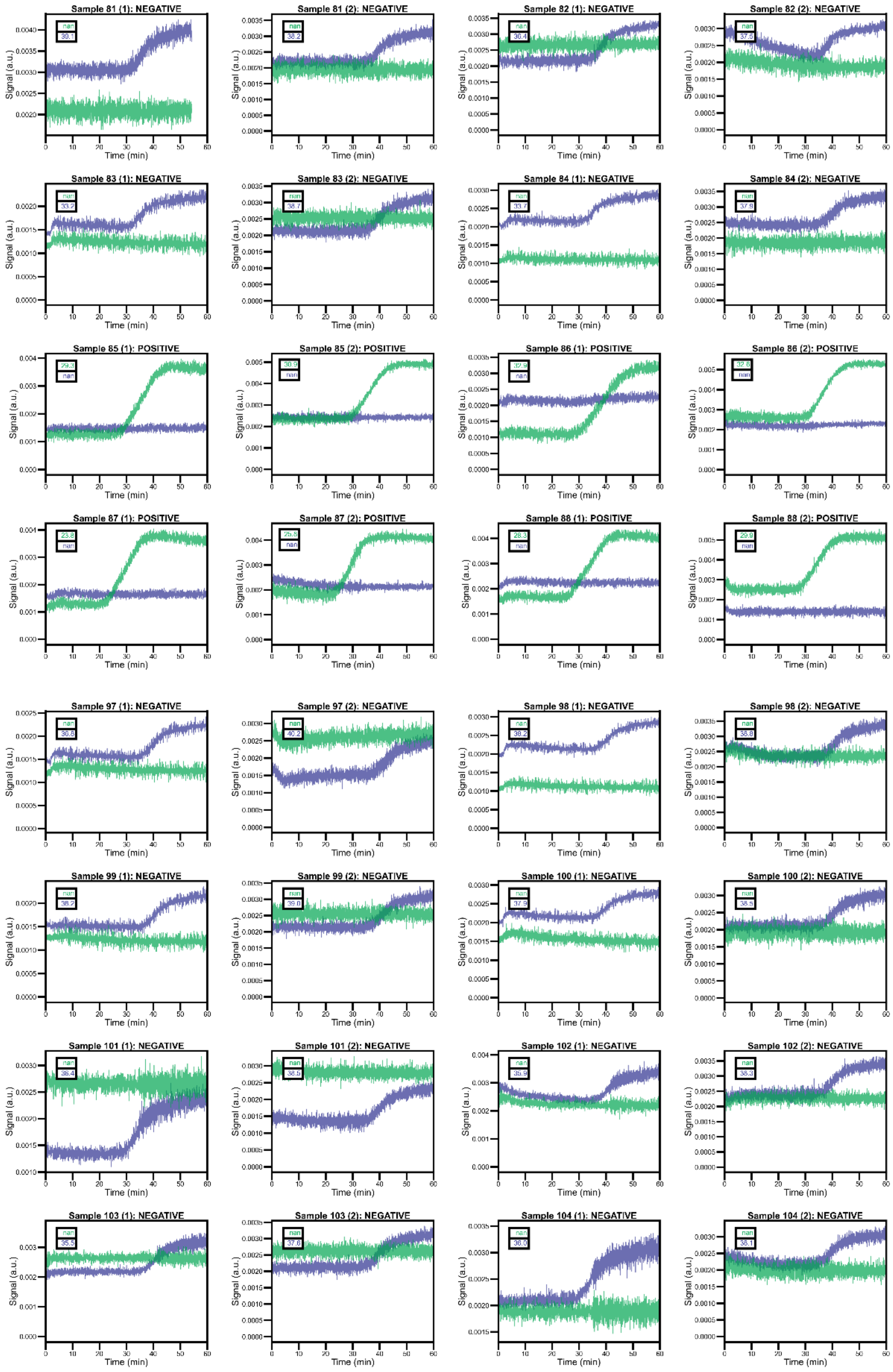
Sample 81 (1): NEGATIVE
Sample 81 (2): NEGATIVE
Sample 82 (1): NEGATIVE
Sample 82 (2): NEGATIVE
Sample 83 (1): NEGATIVE
Sample 83 (2): NEGATIVE
Sample 84 (1): NEGATIVE
Sample 84 (2): NEGATIVE
Sample 85 (1): POSITIVE
Sample 85 (2): POSITIVE
Sample 86 (1): POSITIVE
Sample 86 (2): POSITIVE
Sample 87 (1): POSITIVE
Sample 87 (2): POSITIVE
Sample 88 (1): POSITIVE
Sample 88 (2): POSITIVE
Sample 97 (1): NEGATIVE
Sample 97 (2): NEGATIVE
Sample 98 (1): NEGATIVE
Sample 98 (2): NEGATIVE
Sample 99 (1): NEGATIVE
Sample 99 (2): NEGATIVE
Sample 100 (1): NEGATIVE
Sample 100 (2): NEGATIVE
Sample 101 (1): NEGATIVE
Sample 101 (2): NEGATIVE
Sample 102 (1): NEGATIVE
Sample 102 (2): NEGATIVE
Sample 103 (1): NEGATIVE
Sample 103 (2): NEGATIVE
Sample 104 (1): NEGATIVE
Sample 104 (2): NEGATIVE
Signal (a.u.)
Time (min)

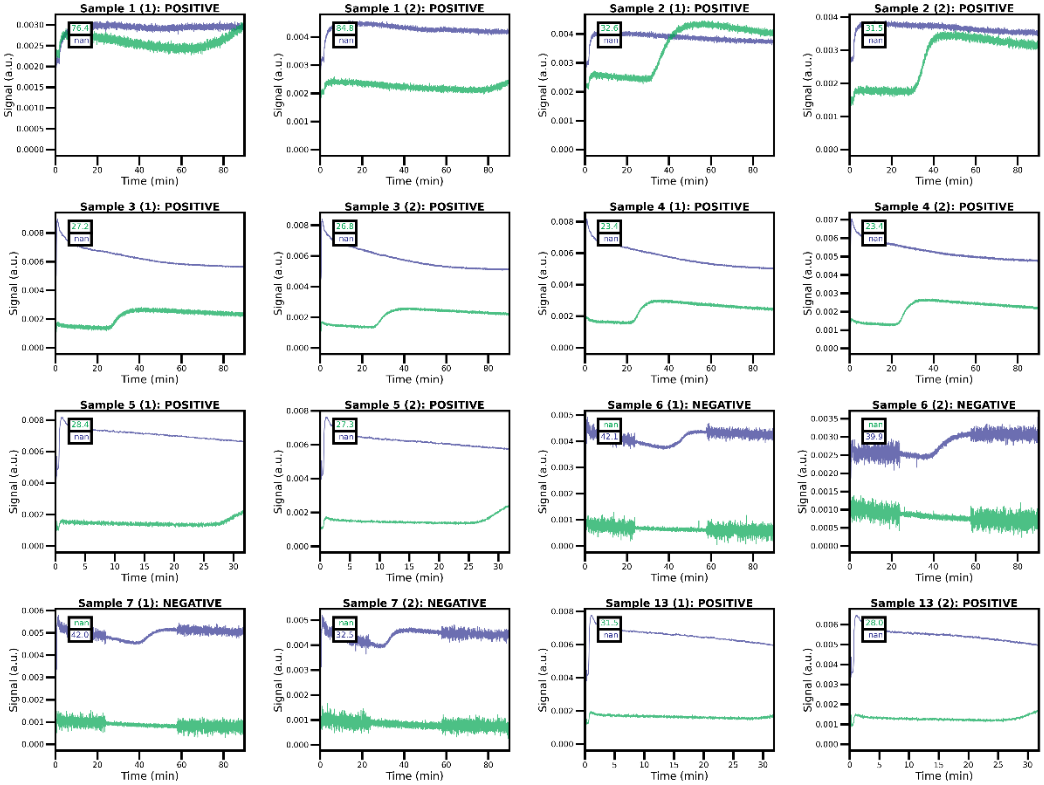
Sample 1 (1): POSITIVE
Sample 1 (2): POSITIVE
Sample 2 (1): POSITIVE
Sample 2 (2): POSITIVE
Sample 3 (1): POSITIVE
Sample 3 (2): POSITIVE
Sample 4 (1): POSITIVE
Sample 4 (2): POSITIVE
Sample 5 (1): POSITIVE
Sample 5 (2): POSITIVE
Sample 6 (1): NEGATIVE
Sample 6 (2): NEGATIVE
Sample 7 (1): NEGATIVE
Sample 7 (2): NEGATIVE
Sample 13 (1): POSITIVE
Sample 13 (2): POSITIVE
Signal (a.u.)
Time (min)

C

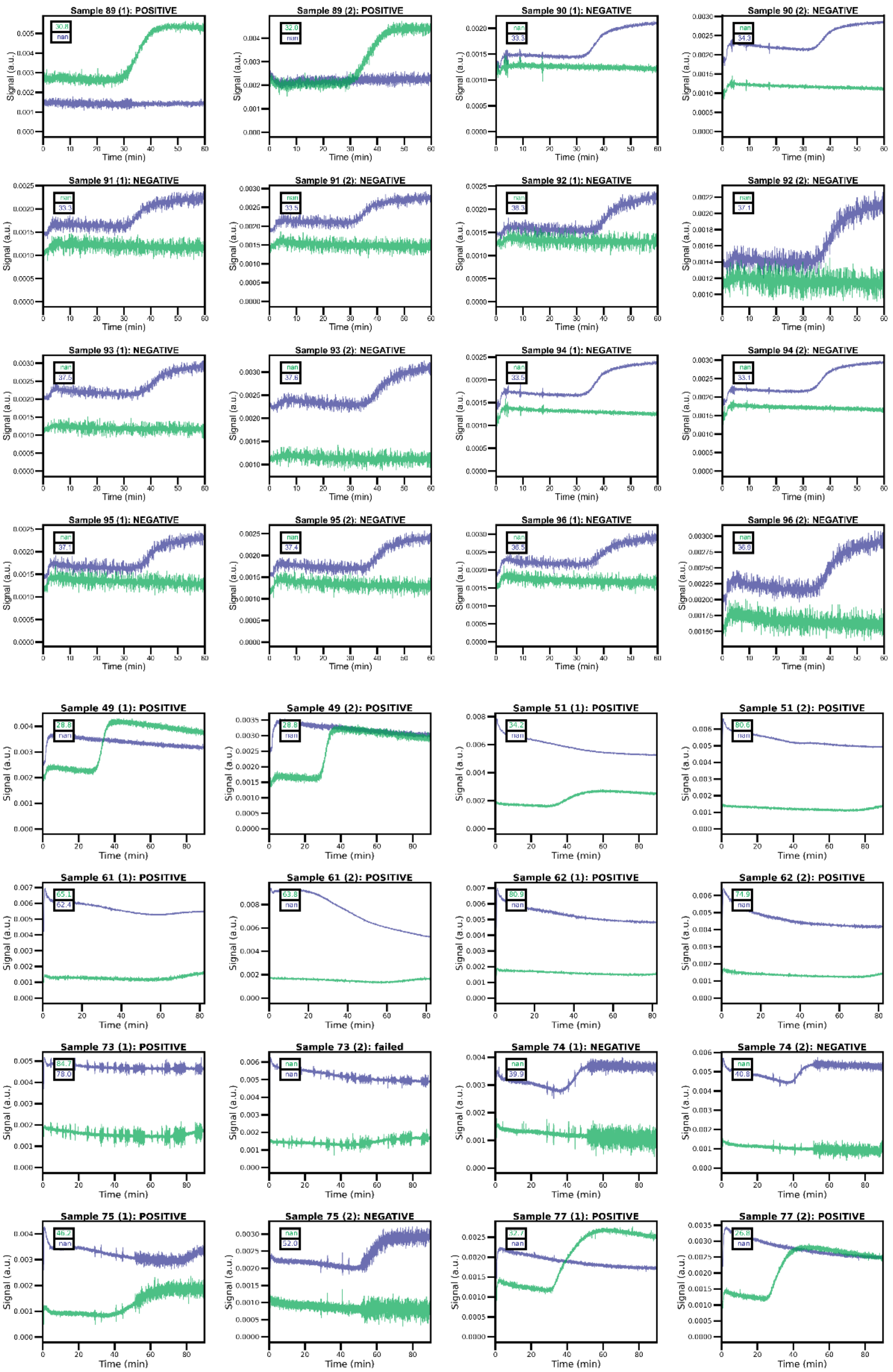
Sample 89 (1): POSITIVE
Sample 89 (2): POSITIVE
Sample 90 (1): NEGATIVE
Sample 90 (2): NEGATIVE
Sample 91 (1): NEGATIVE
Sample 91 (2): NEGATIVE
Sample 92 (1): NEGATIVE
Sample 92 (2): NEGATIVE
Sample 93 (1): NEGATIVE
Sample 93 (2): NEGATIVE
Sample 94 (1): NEGATIVE
Sample 94 (2): NEGATIVE
Sample 95 (1): NEGATIVE
Sample 95 (2): NEGATIVE
Sample 96 (1): NEGATIVE
Sample 96 (2): NEGATIVE
Sample 49 (1): POSITIVE
Sample 49 (2): POSITIVE
Sample 51 (1): POSITIVE
Sample 51 (2): POSITIVE
Sample 61 (1): POSITIVE
Sample 61 (2): POSITIVE
Sample 62 (1): POSITIVE
Sample 62 (2): POSITIVE
Sample 73 (1): POSITIVE
Sample 73 (2): failed
Sample 74 (1): NEGATIVE
Sample 74 (2): NEGATIVE
Sample 75 (1): POSITIVE
Sample 75 (2): NEGATIVE
Sample 77 (1): POSITIVE
Sample 77 (2): POSITIVE
Signal (a.u.)
Time (min)

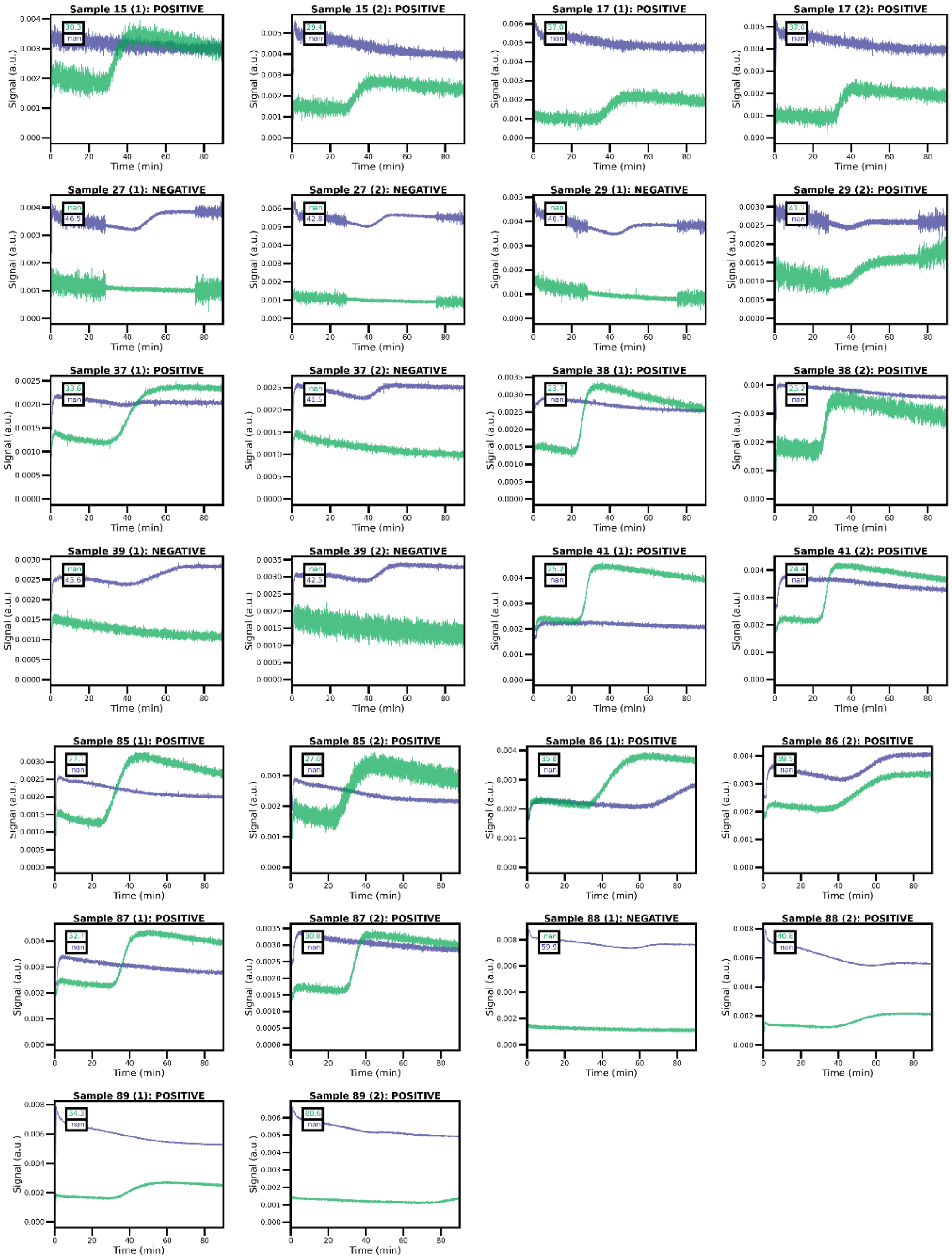

Fig. S8. Real-time amplification curves of samples analyzed by HARMONY Covid-19. (**A**) XPRIZE panel of contrived specimens (**B**) Extracted RNA from VTM specimens (**C**) VTM specimens without extraction. Data were exported from the software as .txt file and plotted using in-house Python code available at https://zenodo.org/record/5502373#.YT7XsZ5KgsM

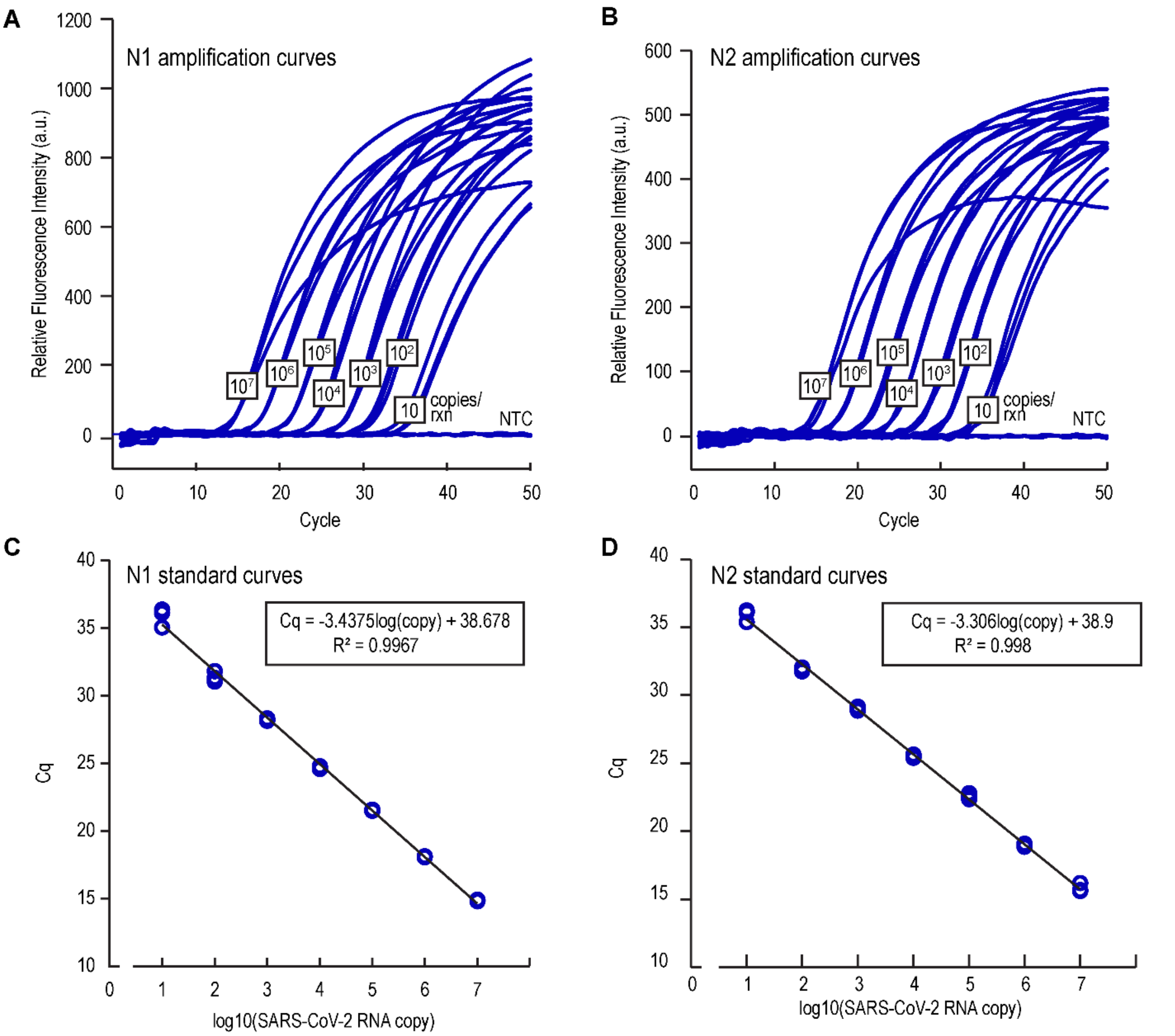

Fig. S9. Standard curves of RT-qPCR using CDC N1 and N2 primers. (**A**) Amplification curves of N1 assay and (**B**) N2 assay ranging from 0 to 10^7 copies/reaction (rxn) of SARS-CoV-2 synthetic RNA. Calibration curves for (**C**) N1 and (**D**) N2 assays.

A

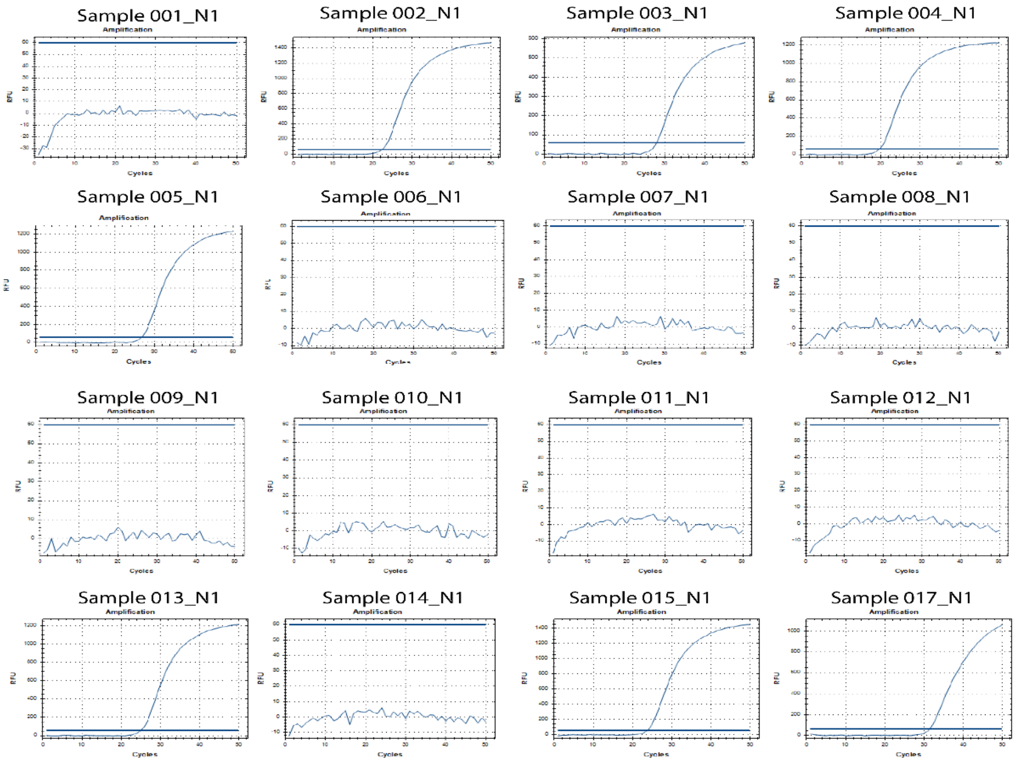
Sample 001_N1
Sample 002_N1
Sample 003_N1
Sample 004_N1
Sample 005_N1
Sample 006_N1
Sample 007_N1
Sample 008_N1
Sample 009_N1
Sample 010_N1
Sample 011_N1
Sample 012_N1
Sample 013_N1
Sample 014_N1
Sample 015_N1
Sample 017_N1

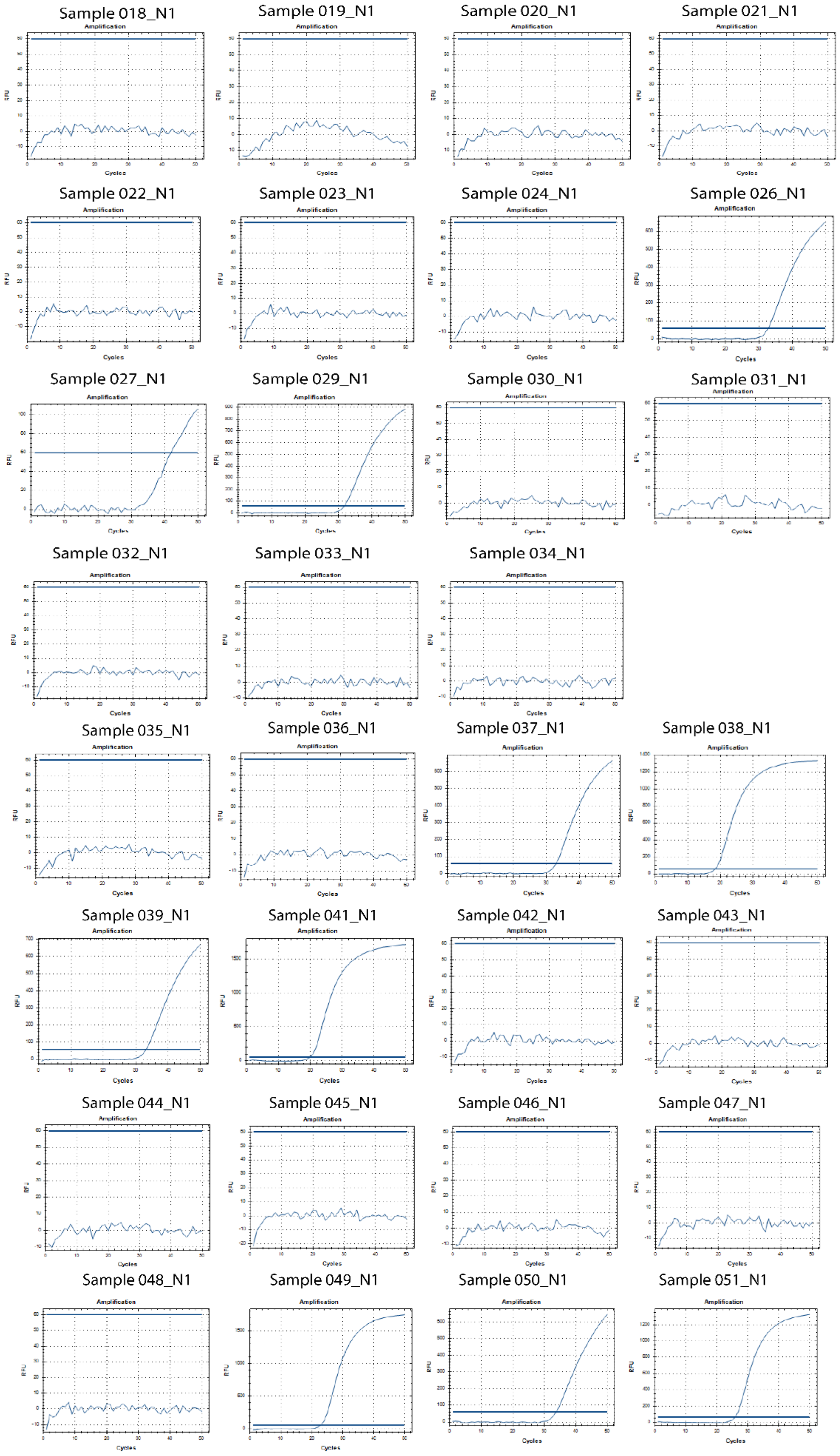
Sample 018_N1
Sample 019_N1
Sample 020_N1
Sample 021_N1
Sample 022_N1
Sample 023_N1
Sample 024_N1
Sample 026_N1
Sample 027_N1
Sample 029_N1
Sample 030_N1
Sample 031_N1
Sample 032_N1
Sample 033_N1
Sample 034_N1
Sample 035_N1
Sample 036_N1
Sample 037_N1
Sample 038_N1
Sample 039_N1
Sample 041_N1
Sample 042_N1
Sample 043_N1
Sample 044_N1
Sample 045_N1
Sample 046_N1
Sample 047_N1
Sample 048_N1
Sample 049_N1
Sample 050_N1
Sample 051_N1
Amplification
RFU
Cycles

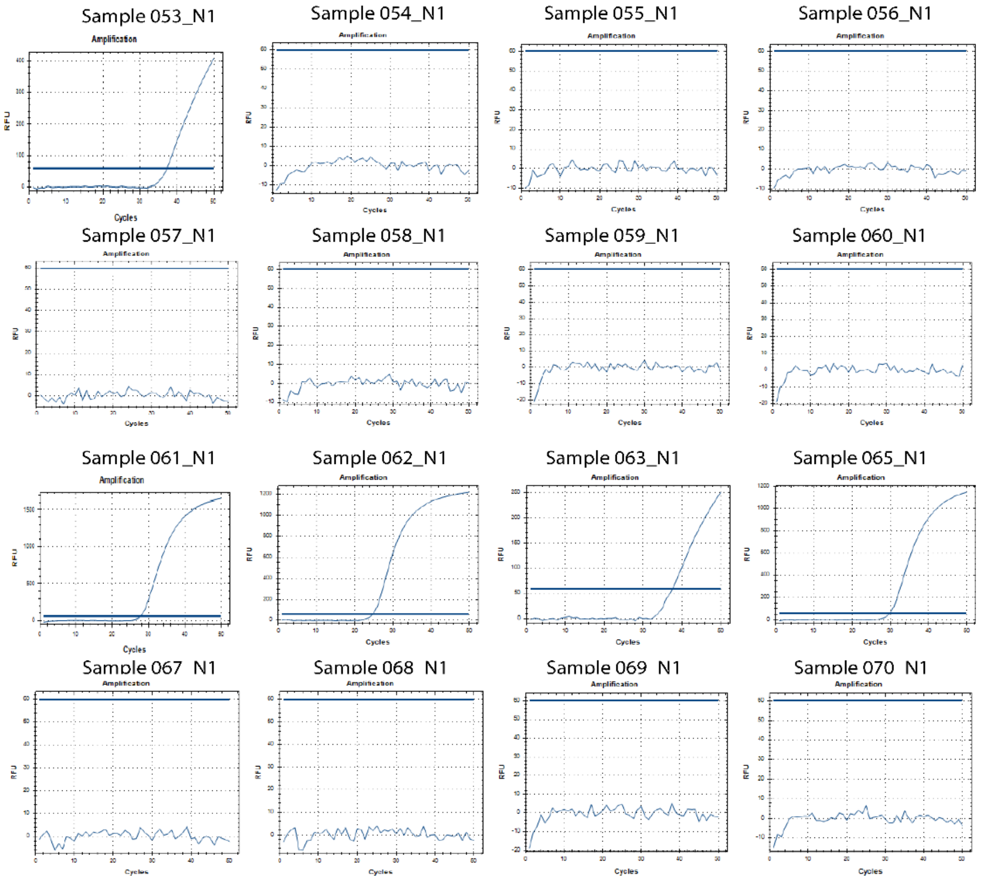
Sample 053_N1
Sample 054_N1
Sample 055_N1
Sample 056_N1
Sample 057_N1
Sample 058_N1
Sample 059_N1
Sample 060_N1
Sample 061_N1
Sample 062_N1
Sample 063_N1
Sample 065_N1
Sample 067_N1
Sample 068_N1
Sample 069_N1
Sample 070_N1
Amplification
RFU
Cycles

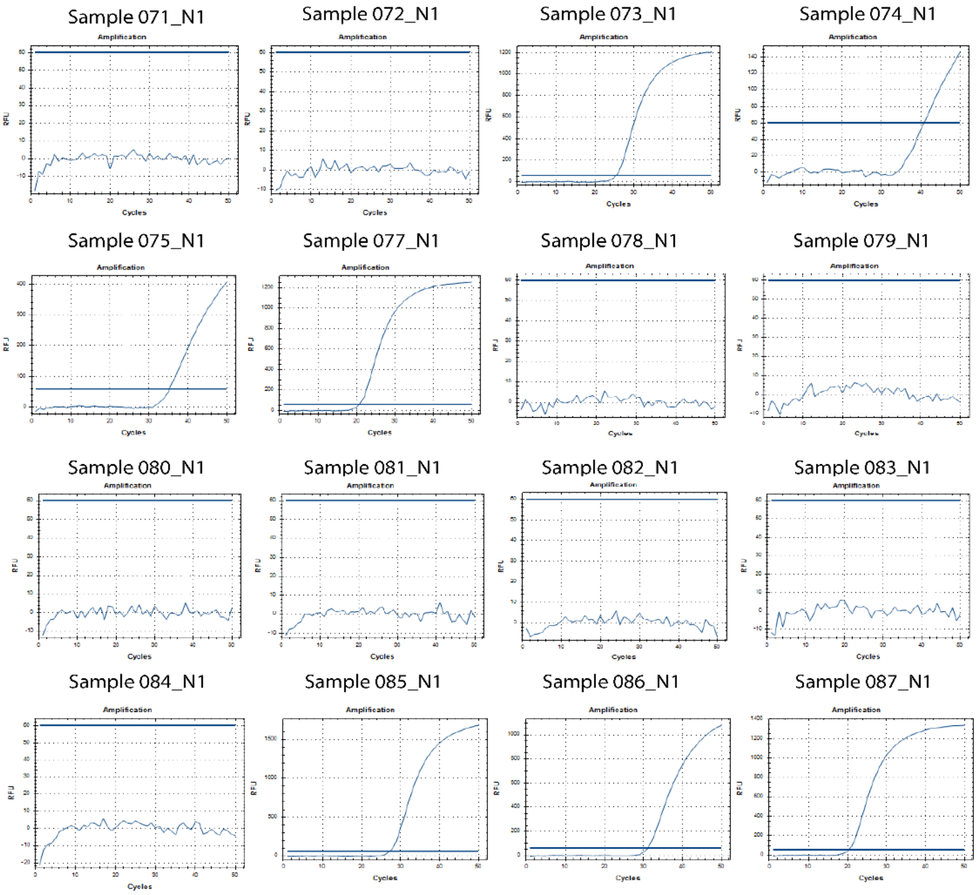
Sample 071_N1
Sample 072_N1
Sample 073_N1
Sample 074_N1
Sample 075_N1
Sample 077_N1
Sample 078_N1
Sample 079_N1
Sample 080_N1
Sample 081_N1
Sample 082_N1
Sample 083_N1
Sample 084_N1
Sample 085_N1
Sample 086_N1
Sample 087_N1
Amplification
RFU
Cycles

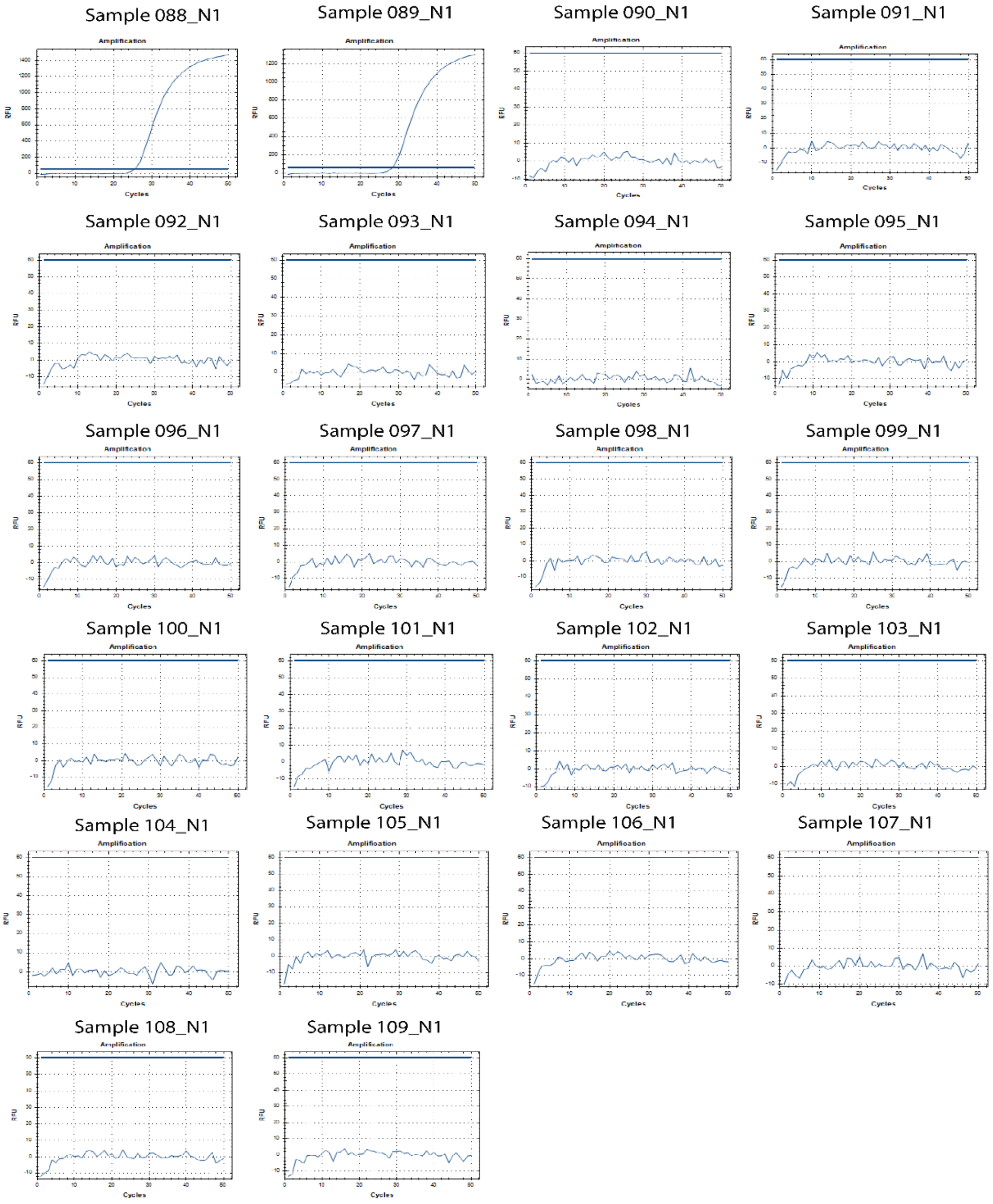
Sample 088_N1
Sample 089_N1
Sample 090_N1
Sample 091_N1
Sample 092_N1
Sample 093_N1
Sample 094_N1
Sample 095_N1
Sample 096_N1
Sample 097_N1
Sample 098_N1
Sample 099_N1
Sample 100_N1
Sample 101_N1
Sample 102_N1
Sample 103_N1
Sample 104_N1
Sample 105_N1
Sample 106_N1
Sample 107_N1
Sample 108_N1
Sample 109_N1

B

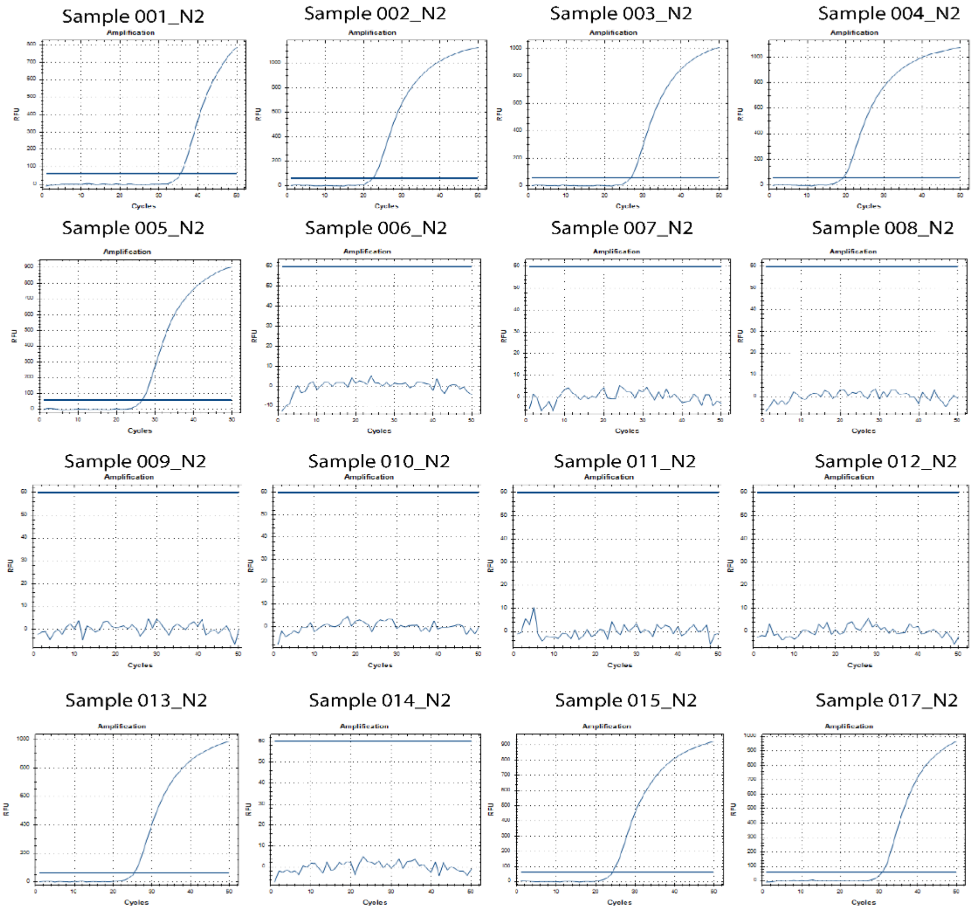
Sample 001_N2
Sample 002_N2
Sample 003_N2
Sample 004_N2
Sample 005_N2
Sample 006_N2
Sample 007_N2
Sample 008_N2
Sample 009_N2
Sample 010_N2
Sample 011_N2
Sample 012_N2
Sample 013_N2
Sample 014_N2
Sample 015_N2
Sample 017_N2
Amplification
RFU
Cycles

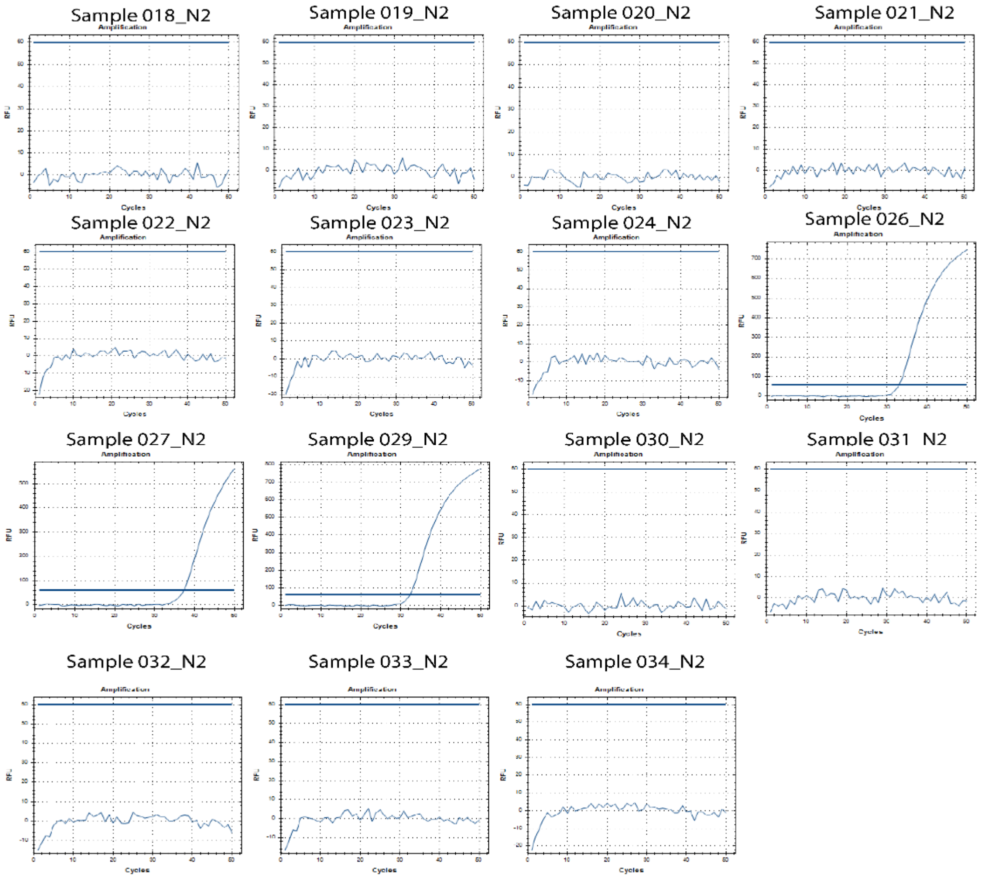
Sample 018_N2
Sample 019_N2
Sample 020_N2
Sample 021_N2
Sample 022_N2
Sample 023_N2
Sample 024_N2
Sample 026_N2
Sample 027_N2
Sample 029_N2
Sample 030_N2
Sample 031_N2
Sample 032_N2
Sample 033_N2
Sample 034_N2

Sample 035_N2

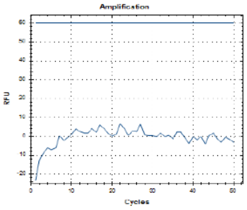

Sample 036_N2

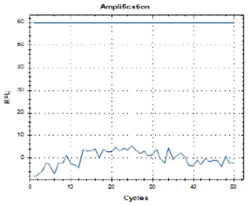

Sample 037_N2

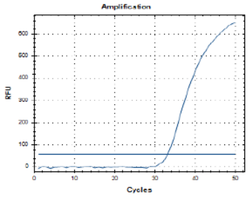

Sample 038_N2

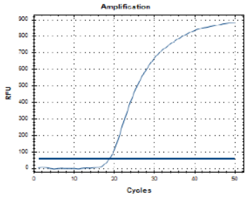

Sample 039_N2

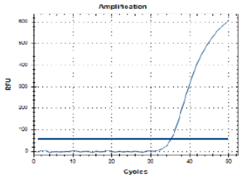

Sample 041_N2

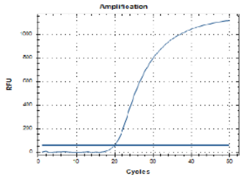

Sample 042_N2

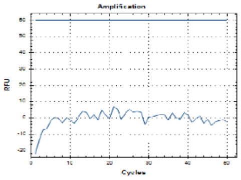

Sample 043_N2

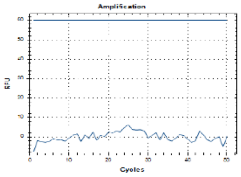

Sample 044_N2

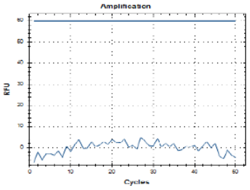

Sample 045_N2

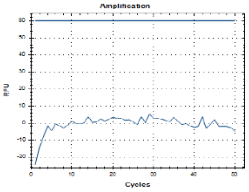

Sample 046_N2

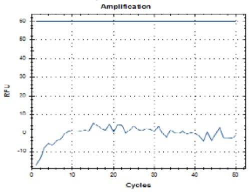

Sample 047_N2

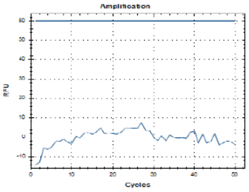

Sample 048_N2

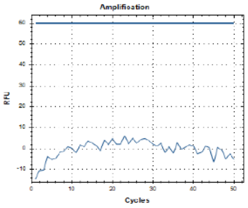

Sample 049_N2

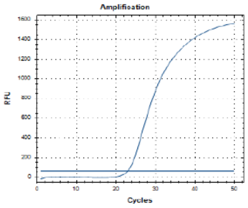

Sample 050_N2

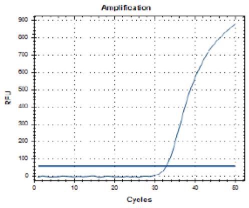

Sample 051_N2

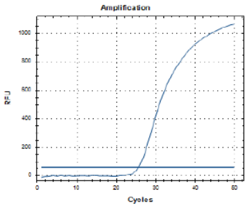

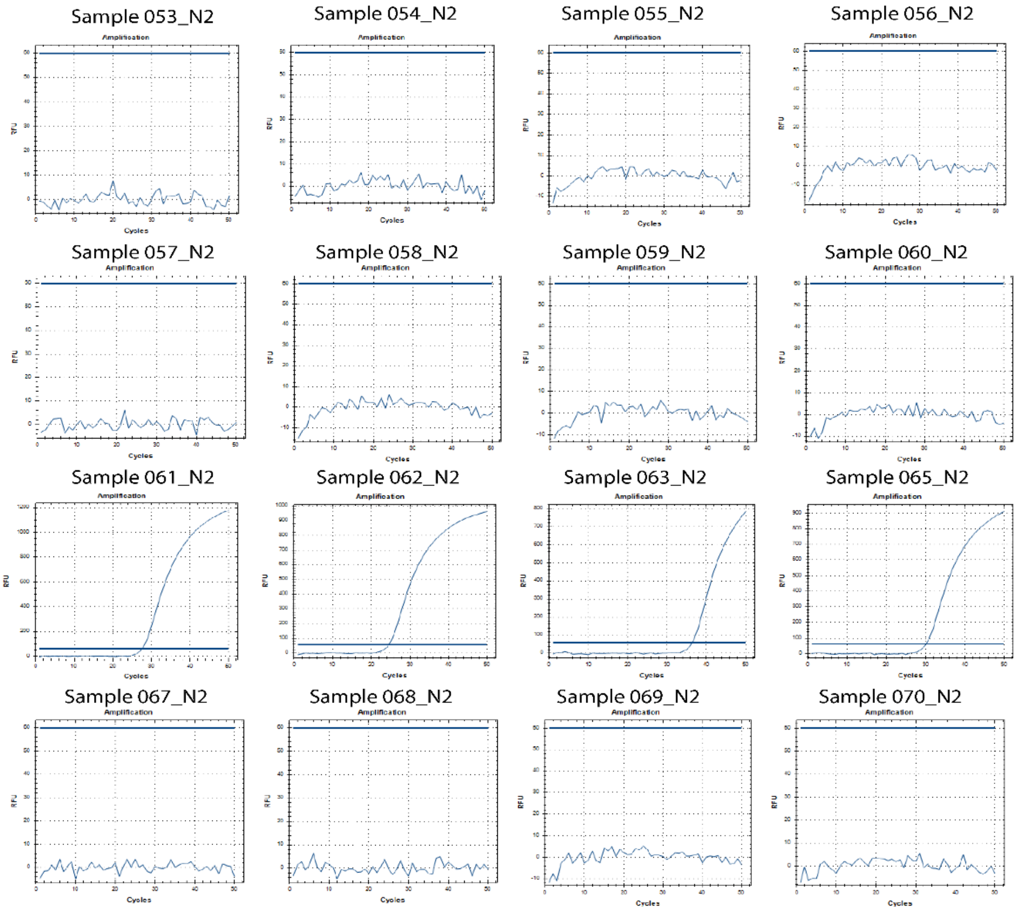
Sample 053_N2
Sample 054_N2
Sample 055_N2
Sample 056_N2
Sample 057_N2
Sample 058_N2
Sample 059_N2
Sample 060_N2
Sample 061_N2
Sample 062_N2
Sample 063_N2
Sample 065_N2
Sample 067_N2
Sample 068_N2
Sample 069_N2
Sample 070_N2
Amplification
RFU
Cycles

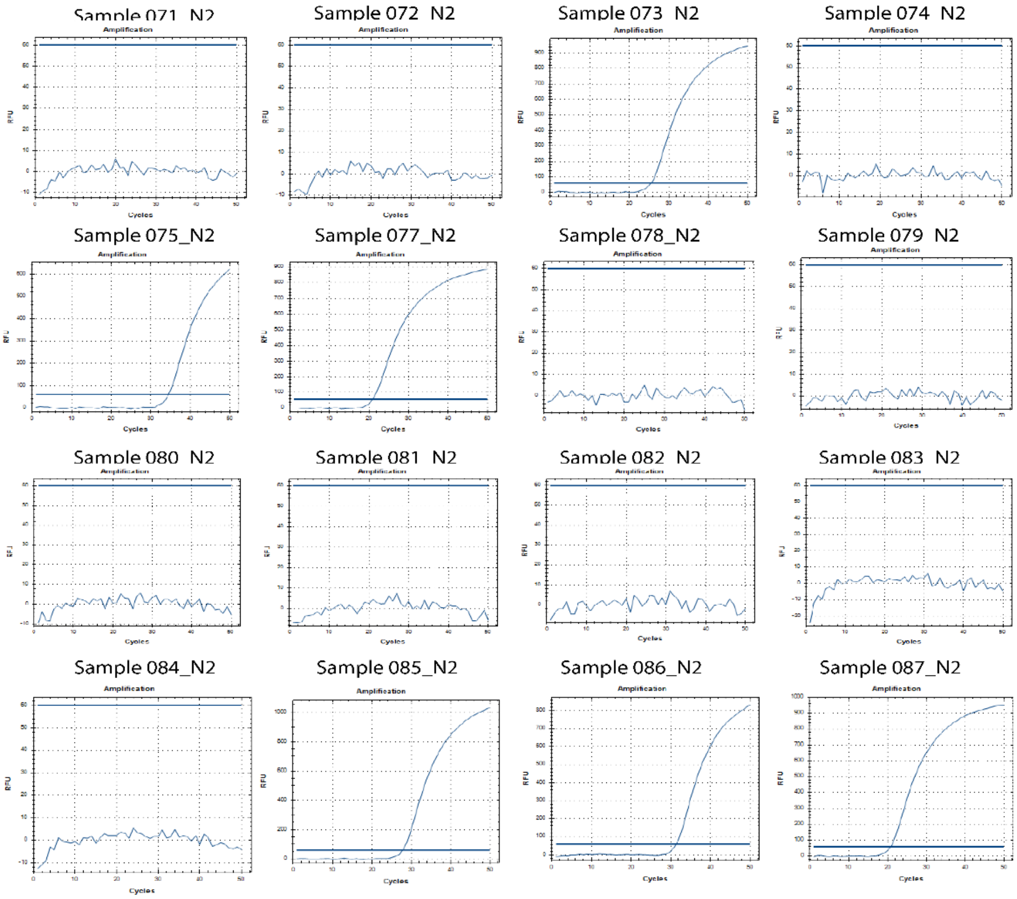
Sample 071_N2
Sample 072_N2
Sample 073_N2
Sample 074_N2
Sample 075_N2
Sample 077_N2
Sample 078_N2
Sample 079_N2
Sample 080_N2
Sample 081_N2
Sample 082_N2
Sample 083_N2
Sample 084_N2
Sample 085_N2
Sample 086_N2
Sample 087_N2
Amplification
RFU
Cycles

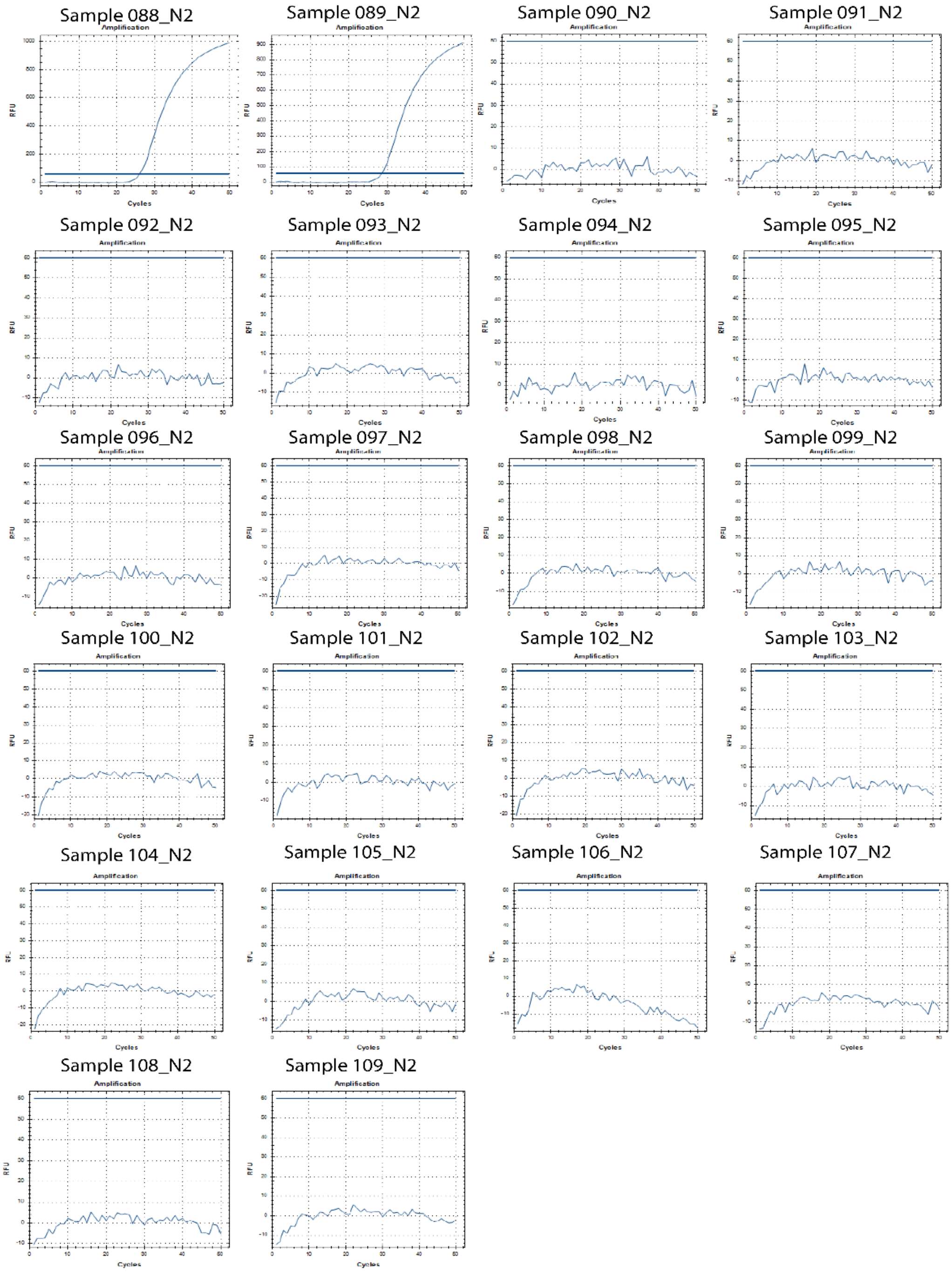
Sample 088_N2
Sample 089_N2
Sample 090_N2
Sample 091_N2
Sample 092_N2
Sample 093_N2
Sample 094_N2
Sample 095_N2
Sample 096_N2
Sample 097_N2
Sample 098_N2
Sample 099_N2
Sample 100_N2
Sample 101_N2
Sample 102_N2
Sample 103_N2
Sample 104_N2
Sample 105_N2
Sample 106_N2
Sample 107_N2
Sample 108_N2
Sample 109_N2
Amplification
RFU
Cycles

C

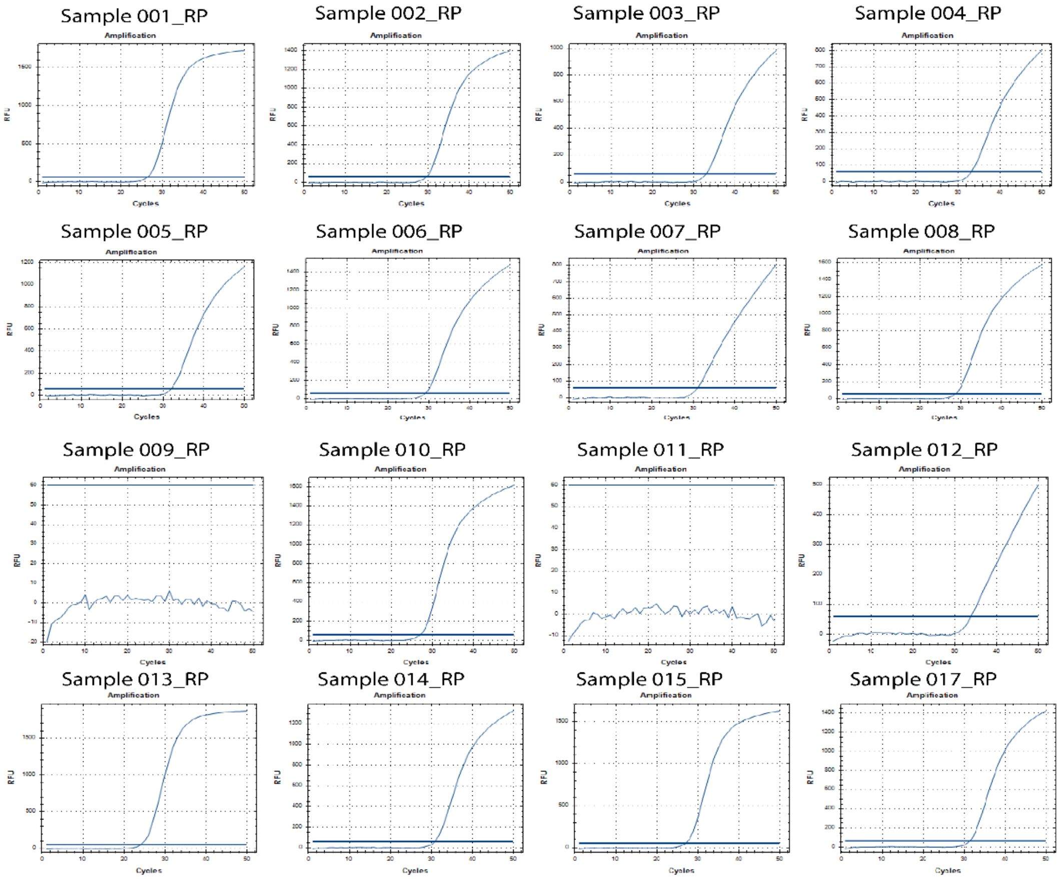
Sample 001_RP
Sample 002_RP
Sample 003_RP
Sample 004_RP
Sample 005_RP
Sample 006_RP
Sample 007_RP
Sample 008_RP
Sample 009_RP
Sample 010_RP
Sample 011_RP
Sample 012_RP
Sample 013_RP
Sample 014_RP
Sample 015_RP
Sample 017_RP
Amplification
RFU
Cycles

Sample 018_RP

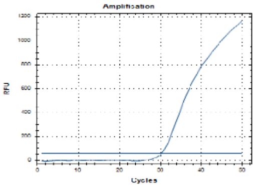

Sample 019_RP

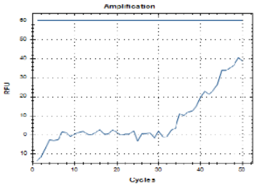

Sample 020_RP

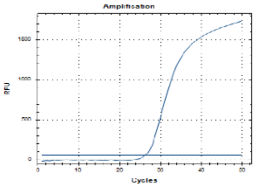

Sample 021_RP

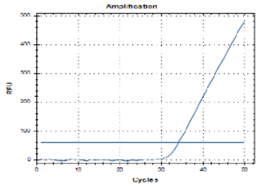

Sample 022_RP

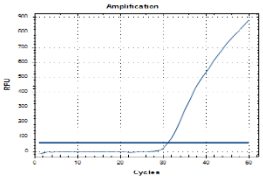

Sample 023_RP

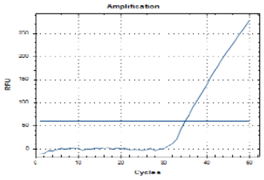

Sample 024_RP

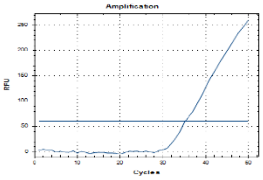

Sample 026_RP

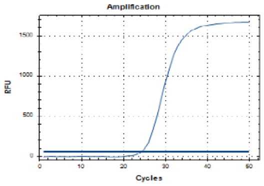

Sample 027_RP

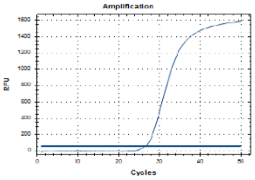

Sample 029_RP

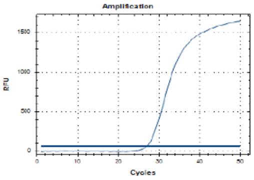

Sample 030_RP

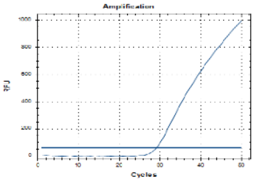

Sample 031_RP

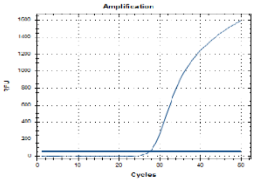

Sample 032_RP

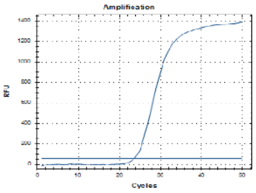

Sample 033_RP

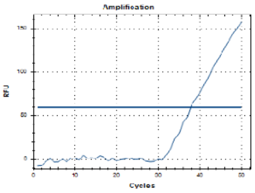

Sample 034_RP

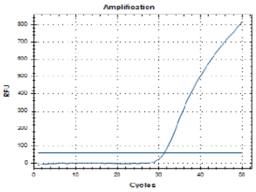

Sample 035_RP

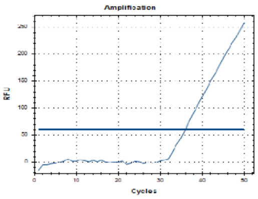

Sample 036_RP

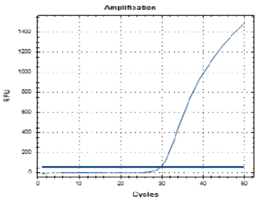

Sample 037_RP

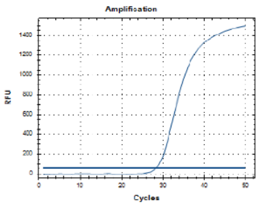

Sample 038_RP

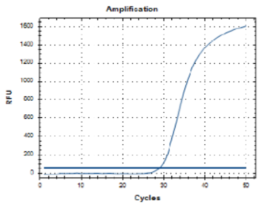

Sample 039_RP

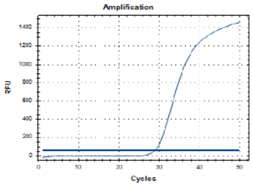

Sample 041_RP

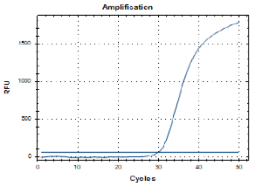

Sample 042_RP

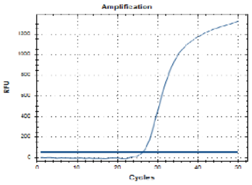

Sample 043_RP

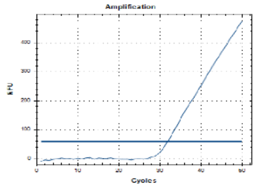

Sample 044_RP

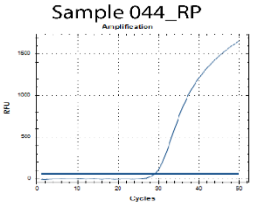

Sample 045_RP

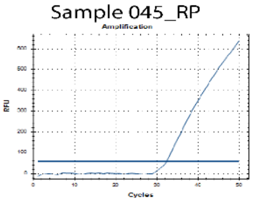

Sample 046_RP

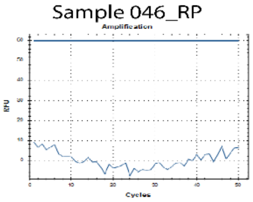

Sample 047_RP

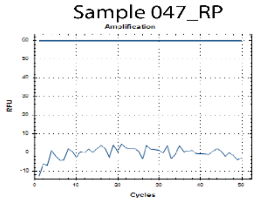

Sample 048_RP

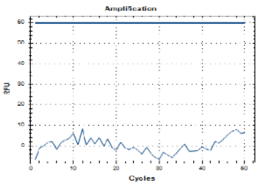

Sample 049_RP

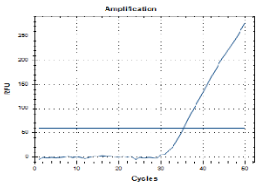

Sample 050_RP

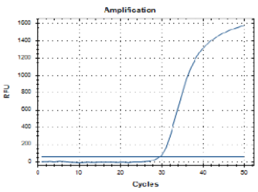

Sample 051_RP

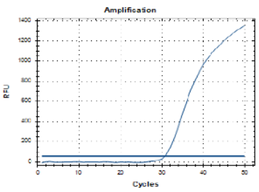

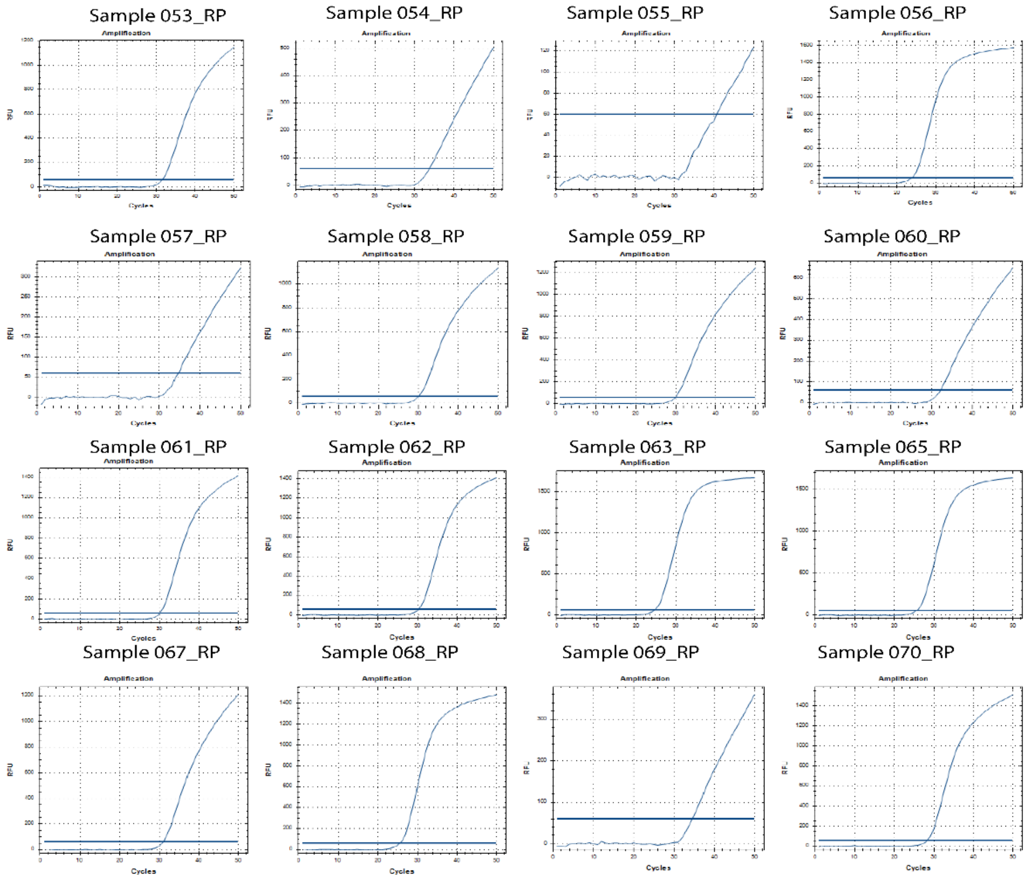
Sample 053_RP
Sample 054_RP
Sample 055_RP
Sample 056_RP
Sample 057_RP
Sample 058_RP
Sample 059_RP
Sample 060_RP
Sample 061_RP
Sample 062_RP
Sample 063_RP
Sample 065_RP
Sample 067_RP
Sample 068_RP
Sample 069_RP
Sample 070_RP
Amplification
RFU
Cycles

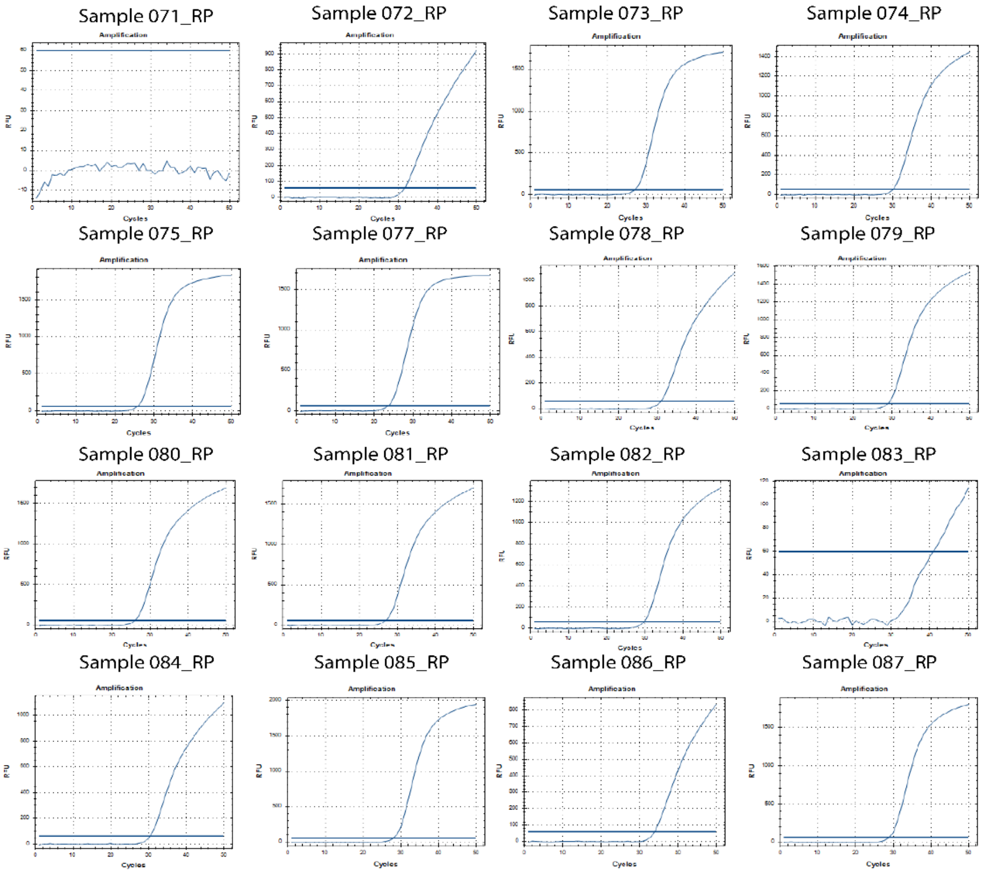
Sample 071_RP
Sample 072_RP
Sample 073_RP
Sample 074_RP
Sample 075_RP
Sample 077_RP
Sample 078_RP
Sample 079_RP
Sample 080_RP
Sample 081_RP
Sample 082_RP
Sample 083_RP
Sample 084_RP
Sample 085_RP
Sample 086_RP
Sample 087_RP
Amplification
RFU
Cycles

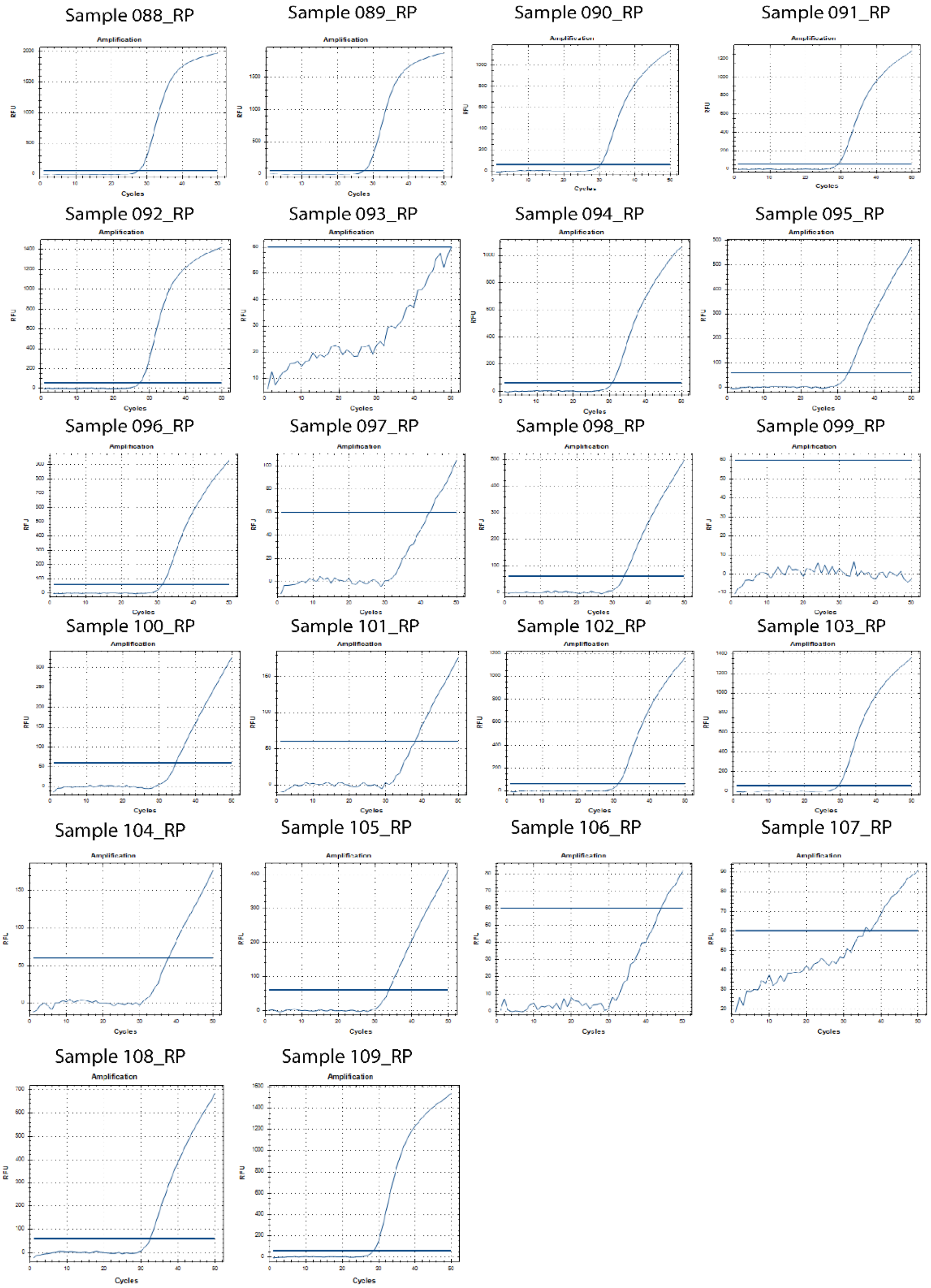

Fig. S10. Real-time amplification curves of RT-qPCR using CDC primers/probes.
(**A**) N1 assay. (**B**) N2 assay. (**C**) RP assay. Data were exported from the CFX Maestro (Biorad).

Table S1.

Summary of RT-LAMP based technologies that have received the US FDA emergency use authorization as of July 15th, 2021. Note that multiple tests were previously approved but recently removed from the list. Information was gathered from the package inserts and patents. Some aspects of the tests are not available, so listed as not available (N/A).

Developer	Intended use[1]	Product name	Core technology	DNA polymerase	SARS-CoV-2 target(s)	Control	# reaction/ sample tested	LAMP multiplexity	Reported LoD				Samples require nucleic acid extraction prior to RT-LAMP	Reagent set up from wet reaction (no lyophilized reaction)	Additional reaction setup after RT-LAMP	Post-reaction analysis
									Copies/uL of unpurified sample	Copies/uL of extracted RNA	Copies/reaction	Copies/uL of reaction				
**Mammoth Biosciences, Inc OR University of California, San Francisco (UCSF) and Mammoth Biosciences**	H	SARS-CoV-2 DETECTR™ Reagent Kit	RT-LAMP, CRISPR	Bst	N	Human RP	2	Single-target LAMP	12	20	100	4	Yes, EZ1 Virus Mini Kit v2.0 with automated extraction	Yes, reagents are thawed at room temperature but kept on ice throughout setting up.	Yes, CRISPR	Yes, based on the difference of end-point fluorescence signal from baseline signal
**MobileDetect Bio Inc.**	H, M	MobileDetect Bio BCC19 (MD-Bio BCC19) Test Kit	RT-LAMP	Not provided	N,E	N/A	1	Two-target, single readout	75	N/A	75	3	No, 1uL VTM sample directly amplified in 25uL reaction.	Yes, reagents must be thawed on ice 30 min prior to set up.	No	Yes, cell phone analysis based on color difference of positive and negative.
**SEASUN BIOMATERIALS, Inc.**	H	AQ-TOP™ COVID-19 Rapid Detection Kit PLUS	RT-LAMP	Bst	N, ORF	Human RP	2	Biplexed FAM/HEX-VIC-JOE with amp control	1	5	50	1.67	Yes, multiple authorized kits	Yes, reagents are thawed at room temperature but kept on ice throughout setting up.	No	Yes, based on automatic CT call by RT-qPCR machine

**Sherlock BioSciences, Inc.**	H	Sherlock™ CRISPR SARS-CoV-2 Kit	RT-LAMP, CRISPR	Not provided	N, ORF	Human RP	3	Single-target LAMP	6.75	45	360	18	Yes, PureLink™ Viral RNA/DNA Mini Kit	Yes, 2 components for RT-LAMP master mix, 8 components for CRISPR reaction	Yes, CRISPR	Yes, manual calculation of signal ratio
**Color Health, Inc.**	H	Color SARS-CoV-2 RT-LAMP Diagnostic Assay	RT-LAMP	Not provided	N, E [2]	Human RP	3	Single-target LAMP	0.75	N/A	N/A	N/A	Yes, Viral DNA/RNA 300 Kit H96 (Perkin Elmer, CMG-1033)	Yes	No	Yes, manual calculation of signal ratio
**Lucira Health, Inc.**	Home, H, M, W	Lucira™ CHECK-IT COVID-19 Test Kit	RT-LAMP	Bst or GspM	N (2 regions)	Multiple chambers detecting the sample, a positive internal control (PIC), and a lysis internal control (LIC).	5	Biplexed LAMP without internal control	0.9	N/A	N/A	N/A	No	No	No	No, real-time analysis based on on the color change of halochromic agent(s) due to pH change as an outcome from amplification

Continued Table. S1.

Developer	Intended use	Product name	Amplicon exposure after LAMP	Estimated operating time	Test batch size (reaction, samples)	Cost	Equipment involved
**Mammoth Biosciences, Inc OR University of California, San Francisco (UCSF) and Mammoth Biosciences**	H	SARS-CoV-2 DETECTR™ Reagent Kit	Yes	**85 min excluding analysis step** 20-min automatic extraction; 30-min RT-LAMP; 15-min CRISPR (anticipated minimal 20-min hands on from thawing, mixing, and aliquoting reactions for both steps)	96 (reaction), 48 (samples)	N/A	• Qiagen EZ1 Advanced benchtop automated extraction (48 samples)<br>• ABI 7500 Fast Dx Real-Time PCR system (Thermo Scientific) with software<br>• Mini Centrifuges<br>• Multichannel or single channel pipette and barrier tips (10 µL – 200 µL);<br>• PCR plate/tube strip support frame/racks<br>• PCR cooler rack
**MobileDetect Bio Inc.**	H, M	MobileDetect Bio BCC19 (MD-Bio BCC19) Test Kit	No	**60 min excluding post-analysis step** 30-min thawing reagents, 30-min RT-LAMP; (anticipated 10-min hands-on from mixing and aliquoting reagents)	8 or 96	N/A	• Custom heater or thermal cycler<br>• Cell phone with software<br>• Minivortex<br>• Reaction set up station<br>• Reaction analysis station<br>• Micropipettes<br>• Filtered tips
**SEASUN BIOMATERIALS, Inc.**	H	AQ-TOP™ COVID-19 Rapid Detection Kit PLUS	No	**70 min excluding analysis step** 30-min manual extraction; 30 min RT-LAMP (anticipated minimal 10-min hands-on from thawing, mixing, and aliquoting reactions)	96 (reaction), 48 (samples)	N/A	• Real-time PCR system (CFX 96 real-time PCR detection system with software CFX manager V3.1 or Applied Biosystems real-time PCR system 7500 with Software 2.0.6)<br>• Centrifuge<br>• Micropipettes<br>• Filtered tips<br>• Vortex
**Sherlock BioSciences, Inc.**	H	Sherlock™ CRISPR SARS-CoV-2 Kit	Yes	**110 min excluding analysis step** 30-min manual extraction; 40 min RT-LAMP; 10-min CRISPR (anticipated minimal 30 -min hands-on from thawing, mixing, and aliquoting reactions)	383(reaction), 30 (samples)	N/A	• Heat block with a heated lid capable of maintaining 61°C or PCR instrument with a heated lid<br>• Vortex<br>• Microcentrifuge<br>• Cold blocks<br>• Micropipettes<br>• Filtered tips
**Color Health, Inc.**	H	Color SARS-CoV-2 RT-LAMP Diagnostic Assay	No	**110 min excluding analysis step** 20-min bead-based RNA extraction; 70 min RT-LAMP; (anticipated minimal 20-min hands-on from thawing, mixing, and aliquoting reactions with robots)	384 (reaction), 128(samples)	N/A	• Hamilton STAR/STARlet automated liquid handler with Venus 4 software<br>• Agilent Bravo automated liquid handling platform with software

							• Perkin Elmer Chemagic 360 extraction instrument platform and Chemagic software v6.3.0.3<br>• Biotek Synergy NEO2 multi-mode microplate reader with Gen5 software v3.9
**Lucira Health, Inc.**	Home, H, M, W	Lucira™ CHECK-IT COVID-19 Test Kit	No	30-min sample run time (with anticipated 2-min handson). Positive samples are called as soon as finished	1	$50/test	• Single-use, disposable, customed heater, reader and sample collecter/transfer with 2 AA batteries

SARS-CoV-2 DETECTR Reagent Kits (*53, 54*)
MobileDetect Bio BCC19 (MD-Bio BCC19) Test Kit (*55, 56*)
AQ-TOP COVID-19 Rapid Detection Kit PLUS (*57*)
Sherlock CRISPR SARS-CoV-2 Kit (*58)*
Color SARS-CoV-2 RT-LAMP Diagnostic Assay (*59*)
Lucira CHECK-IT COVID-19 Test Kit (*60, 61*)

1 H (High complexity CLIA labs), M (Moderate complexity CLIA labs), W (CLIA-waived, patient care settings), Home (used at home)
2 ORF assay is available but removed from the current kits.

Table. S2.

Primers, probes, and control sequences. For primers and probes F2/B2 sequences are underlined, non-template linker sequences are italicized, and adapter sequences are in bold.

**SARS-CoV-2 NC1 Primers**	**Sequence (5' to 3')**
**NC1 FIP**	CCACTGCGTTCTCCATTC*TTTT*<u>CCCCGCATTACGTTTGGT</u>
**NC1 BIP**	GCGATCAAAACAACGTCGG*TTAT*<u>TGCCATGTTGAGTGAGAGCG</u>
**NC1 LF**	TGGTTACTGCCAGTTGAATCT
**NC1 LB + T Adapter**	**ACCAACACCTCACATCACACATAATA**GGTTTACCCAATAATACTGCGTCTTG
**NC1 F3**	TGGACCCCAAAATCAGCG
**NC1 B3**	ATCTGGACTGCTATTGGTGTTA
**SARS-CoV-2 NC2 Primer**	Sequence
**NC2 FIP**	CAGCTTCTGGCCCAGTTCCT<u>GTGGTGGTGACGGTAAAATG</u>
**NC2 BIP**	CTTCCCTATGGTGCTAACAAAG*T*<u>CCAATGTGATCTTTTGGTGTATTCA</u>
**NC2 LF**	GTAGTAGAAATACCATCTTGGACT
**NC2 LB + T Adapter**	**ACCAACACCTCACATCACACATAATA**ATATGGGTTGCAACTGAGGGAG
**NC2 F3**	CTACTACCGAAGAGCTACCAG
**NC2 B3**	GCAGCATTGTTAGCAGGATTG
**SARS-CoV-2 NC3 Primers**	Sequence
**NC3 FIP**	TGTGTAGGTCAACCACGTTC*T*<u>GCTTCAGCGTTCTTCGGA</u>
**NC3 BIP**	GTGCCATCAAATTGGATGACAAAG<u>GTTTTGTATGCGTCAATATGCTTATTCAG</u>
**NC3 LF + T Adapter**	**ACCAACACCTCACATCACACATAATA**TCCATGCCAATGCGCGACA
**NC3 LB**	CCAAATTTCAAAGATCAAGTCAT
**NC3 F3**	GACCAGGAACTAATCAGACAAG
**NC3 B3**	GCTTGAGTTTCATCAGCCTTC
**IAC (NC1) primer**	Sequence
**IAC FL + C Adapter**	**ACCACACCTACCACCACTAATAACTAA**CTCCAGCCATCCTCACCATC
**Target UDP**	Sequence
**CoV UDP Probe**	FITC – CCATCAGCACCAAGACTACCCACCTCGCCACCAA**ACCAACACCTCACATCACACATAATA**
**CoV UDP Quencher**	TTGGTGGCGAGGTGGGTAGTCTTGGTGCTGATGG– Iowa Black® FQ
**Control UDP**	Sequence
**IAC UDP Probe**	Tex615 – CCTGACCACTTCCGAACCCAACCACCTACGACAG**ACCACACCTACCACCACTAATAACTAA**
**IAC UDP Quencher**	CTGTCGTAGGTGGTTGGGTTCGGAAGTGGTCAGG – BHQ2
**IAC Template**	Sequence
**IAC (NC1) ssDNA**	AAT GGA CCC CAA AAT CAG CGA AAT GCA CCC CGC ATT ACG TTT GGT GGA CCC TCT GGA GTC AAT GGG TGG TGC CAG AAT GGA GAA CGC AGT GGG GCG CGA TCA AAA CAA CGT CGG CCC CAA GTT GAT CTC CAG CCA TCC TCA CCA TCG TTC ACC GCT CTC ACT CAA CAT GGC AAG AAT TAA CAC CAA TAG CAG TCC AGA TG

Sample transfer methods at the POC

Volumetric transfer pipettes are used in POC tests, but we have not identified a unified dispenser used in commercial pathogen tests. A unified dispenser integrating the swab and the buffer container in a single unit could reduce opportunities for sample mix-up when multiple samples are processed simultaneously. A simple workflow is crucial, especially in busy settings like clinics. However, in our hands, the in-house built dispenser led to variable dispensed fluid volumes and had a higher failure rate than those of the transfer pipette method. While the unified dispenser has many attractive features, we would not recommend using this in-house assembled dispenser unit until the method has been optimized to achieve a more accurately dispensed volume.

Table. S3.
Feedback from the HCWs

Among HCWs, 30% (3/10) reported problems dispensing the fluid using the unified system. However, 20% (2/10) of HCWs agreed that the unified dispenser offered an advantage in its similarity to other tools used in healthcare settings, and 30% (3/10) HCWs were concerned about contaminating the sample or the environment with the transfer pipette compared to the unified dispenser system. Only 1/10 (10%) HCW preferred the smallest tube (0.2mL) for either method, and 5/10 (50%) reported that larger (1.5mL) tubes were helpful for the unified system, while 40% (4/10) of HCWs said receptacle size did not make a difference when using the transfer pipette, and none reported preference for tube size when using the unified dispenser system. HCWs reported higher confidence in correctly completing the second kit compared to the first kit of each method, indicating a similar learning curve.

(A) User preference for reaction tube size

Note: Cells with 0% response were left empty to aid data visualization.

Question	User response, n (%)				
	0.2 mL	0.5 mL	1.5 mL	0.5mL or 1.5mL	no difference
**Which tube size were you most confident using with the unified dispenser swab?**		4 **(40%)**	5 **(50%)**	1 **(10%)**	
**Which tube size were you most confident using with the transfer pipette?**	1 **(10%)**	4 **(40%)**	1 **(10%)**		4 **(40%)**

(B) User responses to survey questions during sample transfer usability testing.

Users were asked to provide their response on a Likert scale: (1) Not at all, (2) Slightly, (3) Somewhat, (4) Fairly, (5) Completely. Note: Phrasing of the questions below was slightly modified to be clear outside of the context of the written protocol/survey (original survey here). Cells with 0% response were left empty to aid data visualization.

Question	User response, n (%)				
	**(1)**	**(2)**	**(3)**	**(4)**	**(5)**

How confident were you that you added the **correct amount of liquid** to each reaction tube using the **unified dispenser system?**		3 (**30%**)	3 (**30%**)	4 (**40%**)
How confident were you that you completed the protocol correctly using the **unified dispenser system** with the **first kit**?	2 (**20%**)	1 (**10%**)	4 (**40%**)	3 (**30%**)
**How confident were you that you completed the protocol correctly using the unified dispenser system with the second kit?**			5(**50%**)	5 (**50%**)
How confident were you that you added the correct amount of liquid to each reaction tube using the **transfer pipette**?		2 (**20%**)	2(**20%**)	6 (**60%**)
How confident were you that you completed the protocol correctly using the **transfer pipette** with the **first kit**?		3 (**30%**)	4 (**40%**)	3 (**30%**)
How confident were you that you completed the protocol correctly using the **transfer pipette** with the **second kit**?			5 (**50%**)	5 (**50%**)
How confident were you that you completed the **on-screen instructions correctly**?		1 (**10%**)	4 (**40%**)	5 (**50%**)

(C) User reported errors/challenges between two sample transfer methods. Cells with no response were left empty to aid data visualization.

	**Sample transfer method**	
**Reported error**	Number of occurrences for the **unified dispensing system**	Number of occurrences for the **transfer pipette**
**The transfer method was hard to aim at the receiving reaction tube.**	1	
**Transfer method dripped/leaked before dispensing to the reaction tube.**	2	
**The materials were too small or difficult to handle.**		3
**It was hard to execute specific instructions due to unfamiliarity with the sample transfer device.**	2	1
**Challenging to dispense a consistent amount of fluid.**	3	
**Not confident liquid was dispensed to the tube.**		2
**Concerned about contaminating samples or the environment.**		3

Table. S4.
Detailed reagent and device cost at a production scale of 10,000 units

**Item**	**Supplier**	**Cost/device (US$)**
Detector boards	Macrofab	$7 each x 2 ($14 total)
Main board	Macrofab	$25 each
LED board	Macrofab	$9 each
Lid Heater	Oshpark	$7 each
Heat block	Bryan Willman	$12 each
Housing	Xometry – HP MultiJet Fusion 3D printing, no volume discount	$50 (costs here should be reduced for scale-up production)
Red filters	Newport	$10 each, $40 in total
Green filters	Newport	$20 each, $80 in total
Assembly	N/A	$30 (half an hour assembly time at $60/hour cost)
	**Total**	**$267 per unit**

**Item**	Supplier	Cost/device (US$)
Detector boards	Macrofab	$7 each x 2 ($14 total)
Main board	Macrofab	$25 each
LED board	Macrofab	$9 each
Lid Heater	Oshpark	$7 each
Heat block	Bryan Willman	$12 each
Housing	Xometry – HP MultiJet Fusion 3D printing, no volume discount	$50 (costs here should be reduced for scale-up production)
Red filters	Newport	$10 each, $40 in total
Green filters	Newport	$20 each, $80 in total
Assembly	N/A	$30 (half an hour assembly time at $60/hour cost)
	**Total**	**$267 per unit**

Table S5.
Consumable costs per test

Item	Supplier	Costs per kit (US$)
Sampling components ($2 maximum)	Multiple sources	$2
Desiccant	Multiple sources	$0.5
PCR tube	Multiple sources	Negligible
DNA polymerase	Produced in-house	Negligible
Reverse transcriptase	New England Biolabs	$3.5
Thermostable inorganic pyrophosphatase	New England Biolabs	$0.23
Primers	Integrated DNA Technologies	$0.03
Fluorescent probe/quenchers	Integrated DNA Technologies	$0.24
Triton-X100	Sigma-Aldrich	Negligible
dNTPs	New England Biolabs	$0.38
Mannitol	OPS Diagnostics	Negligible
DL-Dithiothreitol (DTT)	Promega	Negligible
Nuclease-free water	VWR	Negligible
1X Tris low-EDTA buffer	VWR	Negligible
Rnasin ribonuclease inhibitor	Promega	$0.71
DNA internal amplification control	Integrated DNA Technologies	$0.01
**Cost of goods**		**$8.00**

REFERENCES AND NOTES

1. D. Cucinotta, M. Vanelli, WHO declares COVID-19 a pandemic. *Acta Biomed.* **91**, 157–160 (2020).

2. World Health Organization, WHO Coronavirus (COVID-19) Dashboard (2021); https://covid19.who.int/.

3. A. Sandford, Coronavirus: Half of humanity now on lockdown as 90 countries call for confinement (Euronews, 2021); www.euronews.com/2020/04/02/coronavirus-in-europe-spain-s-death-toll-hits-10-000-after-record-950-new-deaths-in-24-hou.

4. G. Bonaccorsi, F. Pierri, M. Cinelli, A. Flori, A. Galeazzi, F. Porcelli, A. L. Schmidt, C. M. Valensise, A. Scala, W. Quattrociocchi, F. Pammolli, Economic and social consequences of human mobility restrictions under COVID-19. *Proc. Natl. Acad. Sci. U.S.A.* **117**, 15530–15535 (2020).

5. R. A. Teran, K. A. Walblay, E. L. Shane, S. Xydis, S. Gretsch, A. Gagner, U. Samala, H. Choi, C. Zelinski, S. R. Black, Postvaccination SARS-CoV-2 infections among skilled nursing facility residents and staff members - Chicago, Illinois, December 2020-March 2021. *MMWR Morb. Mortal. Wkly Rep.* **70**, 632–638 (2021).

6. C. M. Brown, J. Vostok, H. Johnson, M. Burns, R. Gharpure, S. Sami, R. T. Sabo, N. Hall, A. Foreman, P. L. Schubert, G. R. Gallagher, T. Fink, L. C. Madoff, S. B. Gabriel, B. MacInnis, D. J. Park, K. J. Siddle, V. Harik, D. Arvidson, T. Brock-Fisher, M. Dunn, A. Kearns, A. S. Laney, Outbreak of SARS-CoV-2 Infections, Including COVID-19 Vaccine Breakthrough Infections, Associated with Large Public Gatherings — Barnstable County, Massachusetts, July 2021 (US CDC, 2021); www.cdc.gov/mmwr/volumes/70/wr/mm7031e2.htm?s_cid=mm7031e2_w#contribAff.

7. Centers for Disease Control and Prevention, Requirement for Proof of Negative COVID-19 Test or Recovery from COVID-19 for All Air Passengers Arriving in the United States (2021); www.cdc.gov/coronavirus/2019-ncov/travelers/testing-international-air-travelers.html.

8. E. C. Kline, N. Panpradist, I. T. Hull, Q. Wang, A. K. Oreskovic, P. D. Han, L. M. Starita, B. R. Lutz, Multiplex target-redundant RT-LAMP for robust detection of SARS-CoV-2 using novel fluorescent universal displacement probes. medRxiv 2021.08.13.21261995 (2021).

9. V. L. Dao Thi, K. Herbst, K. Boerner, M. Meurer, L. P. M. Kremer, D. Kirrmaier, A. Freistaedter, D. Papagiannidis, C. Galmozzi, M. L. Stanifer, S. Boulant, S. Klein, P. Chlanda, D. Khalid, I. Barreto Miranda, P. Schnitzler, H. G. Kräusslich, M. Knop, S. Anders, A colorimetric RT-LAMP assay and LAMP-sequencing for detecting SARS-CoV-2 RNA in clinical samples. *Sci. Transl. Med.* **12**, eabc7075 (2020).

10. S. Wu, X. Liu, S. Ye, J. Liu, W. Zheng, X. Dong, X. Yin, Colorimetric isothermal nucleic acid detection of SARS-CoV-2 with dye combination. *Heliyon* **7**, e06886 (2021).

11. J. Xu, J. Wang, Z. Zhong, X. Su, K. Yang, Z. Chen, D. Zhang, T. Li, Y. Wang, S. Zhang, S. Ge, J. Zhang, N. Xia, Room-temperature-storable PCR mixes for SARS-CoV-2 detection. *Clin. Biochem.* **84**, 73–78 (2020).

12. N. Panpradist, I. A. Beck, J. Vrana, N. Higa, D. McIntyre, P. S. Ruth, I. So, E. C. Kline, R. Kanthula, A. Wong-On-Wing, J. Lim, D. Ko, R. Milne, T. Rossouw, U. D. Feucht, M. Chung, G. Jourdain, N. Ngo-Giang-Huong, L. Laomanit, J. Soria, J. Lai, E. D. Klavins, L. M. Frenkel, B. R. Lutz, OLA-Simple: A software-guided HIV-1 drug resistance test for low-resource laboratories. *EBioMedicine* **50**, 34–44 (2019).

13. N. Panpradist, I. A. Beck, P. S. Ruth, S. Ávila-Ríos, C. García-Morales, M. Soto-Nava, D. Tapia-Trejo, M. Matías-Florentino, H. E. Paz-Juarez, S. del Arenal-Sanchez, G. Reyes-Terán, B. R. Lutz, L. M. Frenkel, Near point-of-care, point-mutation test to detect drug resistance in HIV-1: A validation study in a Mexican cohort. *AIDS* **34**, 1331–1338 (2020).

14. J. D. Vrana, N. Panpradist, N. Higa, D. Ko, P. Ruth, R. Kanthula, J. J. Lai, Y. Yang, S. R. Sakr, B. Chohan, M. H. Chung, L. M. Frenkel, B. R. Lutz, E. Klavins, I. A. Beck, Implementation of an interactive mobile application to pilot a rapid assay to detect HIV drug resistance mutations in Kenya. *medRxiv* (2021); https://www.medrxiv.org/content/10.1101/2021.05.06.21256654v1.full.pdf+html.

15. B. Moon, M. Jones, J. Valdez, Lyophilized beads containing mannitol (Cepheid, 2021); https://patents.google.com/patent/US20050069898A1/en.

16. D.-C. Nyan, L. E. Ulitzky, N. Cehan, P. Williamson, V. Winkelman, M. Rios, D. R. Taylor, Rapid detection of hepatitis B virus in blood plasma by a specific and sensitive loop-mediated isothermal amplification assay. *Clin. Infect. Dis.* **59**, 16–23 (2014).

17. A. N. Spiess, N. Mueller, R. Ivell, Trehalose is a potent PCR enhancer: Lowering of DNA melting temperature and thermal stabilization of taq polymerase by the disaccharide trehalose. *Clin. Chem.* **50**, 1256–1259 (2004).

18. Y. Mori, K. Nagamine, N. Tomita, T. Notomi, Detection of loop-mediated isothermal amplification reaction by turbidity derived from magnesium pyrophosphate formation. *Biochem. Biophys. Res. Commun.* **289**, 150–154 (2001).

19. K. Tone, R. Fujisaki, T. Yamazaki, K. Makimura, Enhancing melting curve analysis for the discrimination of loop-mediated isothermal amplification products from four pathogenic molds: Use of inorganic pyrophosphatase and its effect in reducing the variance in melting temperature values. *J. Microbiol. Methods* **132**, 41–45 (2017).

20. S. Xie, Y. Yuan, Y. Chai, R. Yuan, Tracing phosphate ions generated during loop-mediated isothermal amplification for electrochemical detection of nosema bombycis genomic DNA PTP1. *Anal. Chem.* **87**, 10268–10274 (2015).

21. I. New England Biolabs, Thermostable Inorganic Pyrophosphatase (2021); www.neb.com/products/m0296-thermostable-inorganic-pyrophosphatase#Product%20Information.

22. T. Lennox, B. E. Slatko, L. E. Sears, Purified thermostable inorganic pyprophosphatase obtainable from thermococcus litoralis (New England Biolabs Inc., 2021); https://patents.google.com/patent/US5861296A/en.

23. U.S. Centers for Disease Control and Prevention, CDC 2019-Novel Coronavirus (2019-nCoV) Real-Time RT-PCR Diagnostic Panel For Emergency Use Only (U.S. Food and Drug Administration, 2021); www.fda.gov/media/134922/download.

24. Thermo Fisher Scientific, OpenArray Technology Overview (2021); www.thermofisher.com/us/en/home/life-science/pcr/real-time-pcr/real-time-openarray/open-array-technology.html.

25. C. B. F. Vogels, A. E. Watkins, C. A. Harden, D. E. Brackney, J. Shafer, J. Wang, C. Caraballo, C. C. Kalinich, I. M. Ott, J. R. Fauver, E. Kudo, P. Lu, A. Venkataraman, M. Tokuyama, A. J. Moore, M Catherine Muenker, A. Casanovas-Massana, J. Fournier, S. Bermejo, M. Campbell, R. Datta, A. Nelson; Yale IMPACT Research Team, C. S. Dela Cruz, A. I. Ko, A. Iwasaki, H. M. Krumholz, J D Matheus, P. Hui, C. Liu, S. F. Farhadian, R. Sikka, A. L. Wyllie, N. D. Grubaugh, SalivaDirect: A simplified and flexible platform to enhance SARS-CoV-2 testing capacity. *Med* **2**, 263–280.e6 (2021).

26. R. Wolfel, V. M. Corman, W. Guggemos, M. Seilmaier, S. Zange, M. A. Müller, D. Niemeyer, T. C. Jones, P. Vollmar, C. Rothe, M. Hoelscher, T. Bleicker, S. Brünink, J. Schneider, R. Ehmann, K. Zwirglmaier, C. Drosten, C. Wendtner, Virological assessment of hospitalized patients with COVID-2019. *Nature* **581**, 465–469 (2020).

27. J. Zhu, J. Guo, Y. Xu, X. Chen, Viral dynamics of SARS-CoV-2 in saliva from infected patients. *J. Infect.* **81**, e48–e50 (2020).

28. B. Pang, J. Xu, Y. Liu, H. Peng, W. Feng, Y. Cao, J. Wu, H. Xiao, K. Pabbaraju, G. Tipples, M. A. Joyce, H. A. Saffran, D. L. Tyrrell, H. Zhang, X. C. Le, Isothermal amplification and ambient visualization in a single tube for the detection of SARS-CoV-2 using loop-mediated amplification and CRISPR technology. *Anal. Chem.* **92**, 16204–16212 (2020).

29. P. Craw, W. Balachandran, Isothermal nucleic acid amplification technologies for point-of-care diagnostics: A critical review. *Lab Chip* **12**, 2469–2486 (2012).

30. E. Gonzalez-Gonzalez, I. Montserrat Lara-Mayorga, I. P. Rodríguez-Sánchez, Y. S. Zhang, S. O. Martínez-Chapa, G. Trujillo-de Santiago, M. M. Alvarez, Colorimetric loop-mediated isothermal amplification (LAMP) for cost-effective and quantitative detection of SARS-CoV-2: The change in color in LAMP-based assays quantitatively correlates with viral copy number. *Anal. Methods* **13**, 169–178 (2021).

31. J. Rodriguez-Manzano, K. Malpartida-Cardenas, N. Moser, I. Pennisi, M. Cavuto, L. Miglietta, A. Moniri, R. Penn, G. Satta, P. Randell, F. Davies, F. Bolt, W. Barclay, A. Holmes, P. Georgiou, Handheld point-of-care system for rapid detection of SARS-CoV-2 extracted RNA in under 20 min. *ACS Cent. Sci.* **7**, 307–317 (2021).

32. Chai Bio (Santa Clara, CA).

33. W. Putri, D. J. Muscatello, M. S. Stockwell, A. T. Newall, Economic burden of seasonal influenza in the United States. *Vaccine* **36**, 3960–3966 (2018).

34. Centers for Diseases Control and Prevention, Estimated influenza illnesses, medical visits, hospitalizations, and deaths in the United States — 2019–2020 influenza season. (1 October 2021); https://www.cdc.gov/flu/about/burden/2019-2020.html.

35. N. Panpradist, Q. Wang, P. S. Ruth, J. H. Kotnik, A. K. Oreskovic, A. Miller, S. W. A. Stewart, J. Vrana, P. D. Han, I. A. Beck, L. M. Starita, L. M. Frenkel, B. R. Lutz, Simpler and faster Covid-19 testing: Strategies to streamline SARS-CoV-2 molecular assays. *EBioMedicine* **64**, 103236 (2021).

36. G. K. Gulati, N. Panpradist, S. W. A. Stewart, I. A. Beck, C. Boyce, A. K. Oreskovic, C. García-Morales, S. Avila-Ríos, P. D. Han, G. Reyes-Terán, L. M. Starita, L. M. Frenkel, B. R. Lutz, J. J. Lai, Inexpensive workflow for simultaneous monitoring of HIV viral load and detection of SARS-CoV-2 infection. *medRxiv* 2021.08.18.21256786 (2021).

37. N. Morant, Novel thermostable DNA polymerases for isothermal DNA amplification. Thesis, University of Bath (2015).

38. Addgene, Ligation Independent Cloning (2021); www.addgene.org/protocols/lic/.

39. N. Panpradist, B. J. Toley, X. Zhang, S. Byrnes, J. R. Buser, J. A. Englund, B. R. Lutz, Swab sample transfer for point-of-care diagnostics: Characterization of swab types and manual agitation methods. *PLOS ONE* **9**, e105786 (2014).

40. R. Mack, J. B. Robinson, When novices elicit knowledge: Question asking in designing, evaluating, and learning to use software, in *The Psychology of Expertise: Cognitive Research and Empirical AI*, R. R. Hoffman, Ed. (Springer, 1992), pp. 245–268.

41. FPbase, Spectra viewer (2020); www.fpbase.org/spectra/.

42. Newport, Optical Filters (2021); www.newport.com/c/optical-filters.

43. Mousers Electronics, LED lighting (2021); www.mouser.com/Optoelectronics/LED-Lighting/_/N-74g9t.

44. A. Alekseenko, D. Barrett, Y. Pareja-Sanchez, R. J. Howard, E. Strandback, H. Ampah-Korsah, U. Rovšnik, S. Zuniga-Veliz, A. Klenov, J. Malloo, S. Ye, X. Liu, B. Reinius, S. J. Elsässer, T. Nyman, G. Sandh, X. Yin, V. Pelechano, Direct detection of SARS-CoV-2 using non-commercial RT-LAMP reagents on heat-inactivated samples. *Sci. Rep.* **11**, 1820 (2021).

45. B. A. Rabe, C. Cepko, SARS-CoV-2 detection using isothermal amplification and a rapid, inexpensive protocol for sample inactivation and purification. *Proc. Natl. Acad. Sci. U.S.A.* **117**, 24450–24458 (2020).

46. S. Wei, E. Kohl, A. Djandji, S. Morgan, S. Whittier, M. Mansukhani, E. Hod, M. D'Alton, Y. Suh, Z. Williams, Direct diagnostic testing of SARS-CoV-2 without the need for prior RNA extraction. *Sci. Rep.* **11**, 2402 (2021).

47. L. Bokelmann, O. Nickel, T. Maricic, S. Pääbo, M. Meyer, S. Borte, S. Riesenberg, Point-of-care bulk testing for SARS-CoV-2 by combining hybridization capture with improved colorimetric LAMP. *Nat. Commun.* **12**, 1467 (2021).

48. W. Yamazaki, Y. Matsumura, U. Thongchankaew-Seo, Y. Yamazaki, M. Nagao, Development of a point-of-care test to detect SARS-CoV-2 from saliva which combines a simple RNA extraction method with colorimetric reverse transcription loop-mediated isothermal amplification detection. *J. Clin. Virol.* **136**, 104760 (2021).

49. J. Qian, S. A. Boswell, C. Chidley, Z. X. Lu, M. E. Pettit, B. L. Gaudio, J. M. Fajnzylber, R. T. Ingram, R. H. Ward, J. Z. Li, M. Springer, An enhanced isothermal amplification assay for viral detection. *Nat. Commun.* **11**, 5920 (2020).

50. M. Patchsung, K. Jantarug, A. Pattama, K. Aphicho, S. Suraritdechachai, P. Meesawat, K. Sappakhaw, N. Leelahakorn, T. Ruenkam, T. Wongsatit, N. Athipanyasilp, B. Eiamthong, B. Lakkanasirorat, T. Phoodokmai, N. Niljianskul, D. Pakotiprapha, S. Chanarat, A. Homchan, R. Tinikul, P. Kamutira, K. Phiwkaow, S. Soithongcharoen, C. Kantiwiriyawanitch, V. Pongsupasa, D. Trisrivirat, J. Jaroensuk, T. Wongnate, S. Maenpuen, P. Chaiyen, S. Kamnerdnakta, J. Swangsri, S. Chuthapisith, Y. Sirivatanauksorn, C. Chaimayo, R. Sutthent, W. Kantakamalakul, J. Joung, A. Ladha, X. Jin, J. S. Gootenberg, O. O. Abudayyeh, F. Zhang, N. Horthongkham, C. Uttamapinant, Clinical validation of a Cas13-based assay for the detection of SARS-CoV-2 RNA. *Nat. Biomed. Eng.* **4**, 1140–1149 (2020).

51. J. Joung, A. Ladha, M. Saito, M. Segel, R. Bruneau, M.-L. W. Huang, N.-G. Kim, X. Yu, J. Li, B. D. Walker, A. L. Greninger, K. R. Jerome, J. S. Gootenberg, O. O. Abudayyeh, F. Zhang, Point-of-care testing for COVID-19 using SHERLOCK diagnostics. *medRxiv*, 2020.05.04.20091231 (2020).

52. J. P. Broughton, X. Deng, G. Yu, C. L. Fasching, V. Servellita, J. Singh, X. Miao, J. A. Streithorst, A. Granados, A. Sotomayor-Gonzalez, K. Zorn, A. Gopez, E. Hsu, W. Gu, S. Miller, C. Y. Pan, H. Guevara, D. A. Wadford, J. S. Chen, C. Y. Chiu, CRISPR-Cas12-based detection of SARS-CoV-2. *Nat. Biotechnol.* **38**, 870–874 (2020).

53. Mammoth Biosciences Inc., INSTRUCTIONS FOR USE SARS-CoV-2 DETECTRTM Reagent Kit (2021); www.fda.gov/media/141765/download.

54. UCSF Health Clinical Laboratories UCSF Clinical Labs at China Basin, SARS-CoV-2 RNA DETECTR Assay (2021); www.fda.gov/media/139937/download.

55. MobileDetect Bio. Inc., MobileDetect-BIO BCC19 Test Kit for SARS-CoV-2 Detection (2021); www.fda.gov/media/141791/download.

56. MobileDetect Bio. Inc. (2021), vol. 2021.

57. SEASUN BIOMATERIALS Inc., AQ-TOP™ COVID-19 Rapid Detection Kit PLUS (2021); www.fda.gov/media/142800/download.

58. Sherlock Biosciences Inc., INSTRUCTIONS FOR USE Sherlock™ CRISPR SARS-CoV-2 kit (2021); www.fda.gov/media/137746/download.

59. Color Health Inc., Color SARS-CoV-2 RT-LAMP Diagnostic Assay EUA Summary (2021); www.fda.gov/media/138249/download.

60. Lucira Health, Lucira™ CHECK-IT COVID-19 Test Kit (2021); www.fda.gov/media/147494/download.

61. D. MitraIvan, K. Dimov, J. R. Waldeisen, Colorimetric Detection of Nucleic Acid Amplification (2021); https://patents.google.com/patent/US20170044599A1/pt-pt.
